# Effect of tranexamic acid on thrombotic events and seizures in bleeding patients: a systematic review and meta-analysis

**DOI:** 10.1186/s13054-021-03799-9

**Published:** 2021-11-01

**Authors:** Shuhei Murao, Hidekazu Nakata, Ian Roberts, Kazuma Yamakawa

**Affiliations:** 1 Department of Traumatology and Acute Critical Medicine, Osaka University Graduate School of Medicine, Suita, Osaka 565-0871 Japan; 2 Division of Trauma and Surgical Critical Care, Osaka General Medical Center, 3-1-56 Bandai-Higashi, Sumiyoshi, Osaka 558-8558 Japan; 3 London School of Hygiene & Tropical Medicine, London, WC1E 7HT UK; 4 Department of Emergency Medicine, Osaka Medical and Pharmaceutical University, 2-7 Daigakumachi, Takatsuki, Osaka 569-8686 Japan

**Keywords:** Tranexamic acid, Bleeding, Surgery, Thrombotic events, Seizure, Meta-analysis

## Abstract

**Background:**

Tranexamic acid (TXA) reduces surgical bleeding and reduces death from bleeding after trauma and childbirth. However, its effects on thrombotic events and seizures are less clear. We conducted a systematic review and meta-analysis to examine the safety of TXA in bleeding patients.

**Methods:**

For this systematic review and meta-analysis, we searched MEDLINE, EMBASE and the Cochrane Central Register of Controlled trials from inception until June 1, 2020. We included randomized trials comparing intravenous tranexamic acid and placebo or no intervention in bleeding patients. The primary outcomes were thrombotic events, venous thromboembolism, acute coronary syndrome, stroke and seizures. A meta-analysis was performed using a random effects model and meta-regression analysis was performed to evaluate how effects vary by dose. We assessed the certainty of evidence using the grading of recommendations, assessment, development and evaluations (GRADE) approach.

**Results:**

A total of 234 studies with 102,681 patients were included in the meta-analysis. In bleeding patients, there was no evidence that TXA increased the risk of thrombotic events (RR = 1.00 [95% CI 0.93–1.08]), seizures (1.18 [0.91–1.53]), venous thromboembolism (1.04 [0.92–1.17]), acute coronary syndrome (0.88 [0.78–1.00]) or stroke (1.12 [0.98–1.27]). In a dose-by-dose sensitivity analysis, seizures were increased in patients receiving more than 2 g/day of TXA (3.05 [1.01–9.20]). Meta-regression showed an increased risk of seizures with increased dose of TXA (*p* = 0.011).

**Conclusion:**

Tranexamic acid did not appear to increase the risk of thrombotic events in bleeding patients. However, because there may be dose-dependent increase in the risk of seizures, very high doses should be avoided.

**Supplementary Information:**

The online version contains supplementary material available at 10.1186/s13054-021-03799-9.

## Background

Tranexamic acid (TXA) is a long-established antifibrinolytic agent that was developed in Japan in 1962 [1, 2]. TXA is a synthetic derivative of the amino acid lysine that inhibits fibrinolysis by blocking the lysine binding sites on plasminogen, which contribute to reduce bleeding [3]. For many years, TXA has been used in surgery to reduce blood loss and the need for transfusion [4]. Recent randomized trials have shown that timely TXA administration reduces death from bleeding in patients with trauma and post-partum hemorrhage [5, 6].

Although the benefits of TXA for reducing mortality and bleeding are well established in acute bleeding [7], its effects on adverse events are less clear. One of primary concerns with TXA administration relates to the potential for thrombotic events and seizures. By reducing fibrinolysis TXA might increase the risk of thrombosis [8]. TXA can cross the blood–brain barrier and might increase seizures by antagonizing inhibitory gamma-aminobutyric acid receptor type A (GABA_A_) in brain [9–11]. Despite the cumulative evidence, there remain uncertainties on the risk of thrombotic events and seizures with TXA use due to imprecision or methodological limitations. [4, 7, 12, 13].

The aim of this systematic review and meta-analysis is to evaluate the effects of TXA on thrombotic events and seizures by analyzing all the available RCTs in bleeding patients. We also evaluated how the effects of TXA vary by dose and underlying disease with subgroup analysis and meta-regression analysis.

## Methods

The protocol for this systematic review was registered on PROSPERO (Registration No. CRD42020160037). The systematic reviews and meta-analyses were performed in accordance with the Preferred Reporting Items for Systematic Reviews and Meta-Analyses (PRISMA) guidelines [14] and adhered pre-published protocol. [15].

### Study criteria

We included trials with the following characteristics (1) study types: randomized trials, (2) population: patients with traumatic, surgical, obstetric, intracranial or gastrointestinal bleeding, (3) intervention: intravenous tranexamic acid at any dose, (4) control: placebo or no intervention (without TXA administration) and (5) outcomes: any thrombotic events (venous thromboembolism, acute coronary syndrome and stroke) and seizures.

### Systematic search

We conducted a comprehensive search of MEDLINE (via PubMed), EMBASE and the Cochrane Central Register of Controlled Trials from inception until, June 1, 2020, for randomized trials comparing intravenous TXA and placebo or no intervention in bleeding patients (trauma, surgery, post-partum hemorrhage, spontaneous intracranial hemorrhage, gastrointestinal hemorrhage). We did not apply any language or time restrictions to the electronic searches. The search strategy in MEDLINE was as follows: (Tranexamic acid [mesh] OR tranexamic acid [tiab] OR TXA [tiab]) AND ((randomized controlled trial [pt] OR controlled clinical trial [pt] OR randomized [tiab] OR clinical trials as topic [mesh: noexp] OR randomly [tiab] OR trial [ti]) NOT (animals [mh] NOT humans [mh])). Our MEDLINE search strategy was adapted for searches in the other two databases (Additional file 1: Table S1). Reference lists of eligible studies were hand-searched to identify further relevant studies.

### Trial selection and data extraction

Two authors (HN, SM) independently screened articles for inclusion on the basis of title and abstracts and reviewed relevant articles as full text. Disagreements were resolved by discussion and referral to a third author (KY) if necessary. Two authors (HN, SM) extracted the study characteristics from each included study, including year of publications, study population, number of participants, name of comparators, dose of treatment, indication for treatment, name and types of adverse events and information assessing risk of bias in the studies.

### Quality assessment

Two authors (HN, SM) independently assessed the risk of systematic errors (bias) of the trials included in the meta-analysis according to the Cochrane Handbook, version 6.1 [16]. To evaluate the risk of bias in the individual RCTs, we will use the revised uniform criteria of the Cochrane risk-of-bias tool for randomized trials ver. 2 (RoB 2).

Risk of bias was rated according to the following domains (1) bias due to randomization, (2) bias due to deviations from indented interventions, (3) bias due to missing data, (4) bias due to outcome measurement, (5) bias due to selection of reported result. Trials adjudicated as having concerns or having high risk of bias for one or more domains were classified as having an overall high risk of bias.

### Outcomes

The primary outcome measure was the number of patients with any thrombotic events, including acute coronary syndrome (ACS), stroke/transient ischemic attack (TIA) and venous thromboembolism (VTE). The number of patients with ACS, stroke/TIA and VTE was also assessed separately. The number of patients with seizures was also measured as primary outcome.

### Statistical analysis

Statistical analyses were performed using Review Manager Software 5 (Review Manager [RevMan] Version 5.4. Copenhagen: The Nordic Cochrane Centre, The Cochrane Collaboration, 2020) and STATA software, V.15.0 (STATA Corporation, College Station, TX, USA). For each included trial, we calculated the relative risks with 95% confidence intervals for all outcome measures. Heterogeneity among the trials was explored by inspection of forest plots and calculation of *I*^2^ statistics [16]. We performed both fixed effects analysis using the Mantel–Haenszel method and random effects analyses using the DerSimonian and Laird estimator, reporting the most conservative summary estimate with the broadest confidence interval [17]. Pre-specified subgroup analyses were performed as standard using RevMan 5 software.

We conducted analysis to establish, first, the effects of TXA on any thrombotic events and on seizures in bleeding patients. We also assessed the effect of TXA on venous thromboembolism, acute coronary syndrome and stroke separately. We examined the following pre-planned sensitivity analyses: type of underlying disease (trauma, cardiac surgery, orthopedic surgery, obstetrics and gynecology, spontaneous intracranial hemorrhage, gastrointestinal bleeding), TXA dose (≤ 2 g or > 2 g per day), sample size (< 500 or ≥ 500), risk of bias (low risk of bias or high risk of bias). Additionally, we conducted subgroup analysis in children. Meta-regression analysis was carried out to investigate the association between the incidence of adverse events and the dose of TXA.

Statistical heterogeneity will be evaluated informally from forest plots of the study estimates and more formally using the chi-squared test (*P* value < 0.1 = significant heterogeneity) and *I*^2^ statistic (*I*^2^ > 50% = significant heterogeneity).

To investigate publication biases, we created funnel plot in which the log RRs were plotted against their standard errors and tested the symmetry of the funnel plots with Begg’s rank correlation test and Egger’s linear regression test.

We also assessed the overall certainty of evidence for each outcome using the grading of recommendations assessment, development and evaluation (GRADE) approach.

## Results

### Literature search

**Fig. 1:**
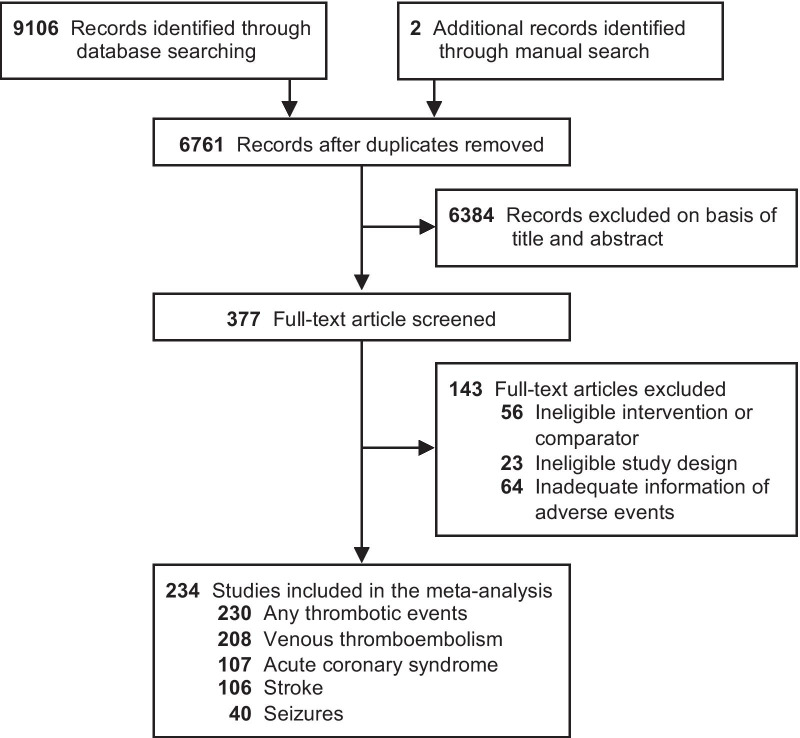
PRISMA flowchart

In total, we screened 6761 abstracts, of which 377 were eligible for full-text review. 234 trials with 102,681 participants were eligible for inclusion in the meta-analysis (Fig. 1).

### Description of included studies

Table 1 summarizes characteristics of included trials. 124 (53%) trials were in orthopedic surgery, 47 trials (20%) were in cardiac surgery, 22 trials (9%) were in obstetric and gynecological surgery, 7 trials (3%) were in trauma (including traumatic brain injury), 9 trials (4%) were in spontaneous (non-traumatic) intracranial hemorrhage, 3 trials (1%) were in gastrointestinal hemorrhage. 13 trials had sample sizes over 500. [5, 6, 12, 18–27] 9 trials involved children. Among 234 trials included in this meta-analysis, data were available on any thrombotic events for 230 trials, on venous thromboembolism for 208 trials, on acute coronary syndrome for 107 trials, on stroke for 106 trials and on seizures for 40 trials.

**Table 1:** Summary characteristics of included trials

	No. of trials *n* = 234	No. of participants *n* = 102,681
*Trial characteristics*		
Multicenter study	26	80,268
Single-center study	208	22,413
*Patient disease type*		
Trauma (including traumatic brain injury)	7	34,488
Obstetrics and gynecology	22	28,089
Cardiac surgery	47	10,528
Orthopedic surgery	124	11,353
Intracranial (non-traumatic) hemorrhage	9	4057
Gastrointestinal hemorrhage	3	12,312
Others	22	1854
*Patient characteristics*		
Mean age (year) of trial arms, median [IQR]	Control: 54 [43–66] Intervention: 50 [46–67]	
Percentage of female patients in trial arms, median [IQR]	Control: 54 [35–70] Intervention: 53 [31–73]	
Adults	225	101,917
Pediatrics	9	764
*TXA dose*		
≦ 2 g/day	180	78,268
2 g/day < TXA dose ≦ 4 g/day	14	13,887
> 4 g	20	7603
Composite	20	2923
*TXA regimen*		
Bolus administration	149	47,973
Continuous infusion	4	785
Bolus + infusion	80	53,848

IQR, interquartile range. TXA, tranexamic acid

### Risk of bias in individual trials

Overall, 109 trials were deemed to be at low risk of bias. 182 trials were judged at low risk of bias for randomization process, 158 trials for deviations from intended interventions, 224 trials for missing outcome data and 125 trials for measurement of outcome result (Additional file 2: Figure S1). Supplementary material is attached showing the characteristics and risk of bias of the included trials (Additional file 1: Table S2; Additional file 2: Figure S2).

### Primary outcome in all hemorrhagic patients

**Fig. 2:**
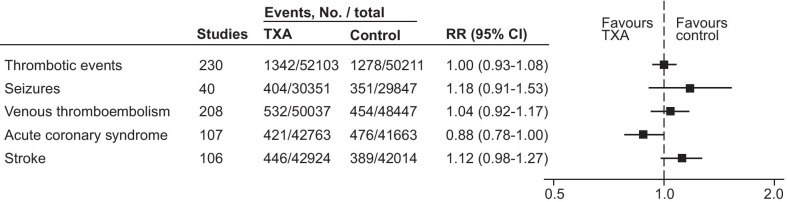
Forest plot comparing tranexamic acid and control for primary outcome. TXA, tranexamic acid. RR, risk ratio. CI, confidence interval

We found no evidence that TXA administration increased the risk of thrombotic events (RR = 1.00 [95% CI 0.93–1.08], *I*^2^ = 0%, high certainty of evidence), seizures (1.18 [0.91–1.53], *I*^2^ = 34%, moderate certainty), venous thromboembolism (1.04 [0.92–1.17], *I*^2^ = 0%), acute coronary syndrome (0.88 [0.78–1.00], *I*^2^ = 0%) or stroke (1.12 [0.98–1.27], *I*^*2*^ = 0%) (Fig. 2). These results were consistent for both random effects and fixed effect statistical model and we observed no evidence of publication bias, either when evaluating the funnel plot (Additional file 2: Figures S3 and S4) or statistically for thrombotic events (Begg's rank correlation test: *p* = 0.12 and Egger's linear regression test: 0.424) and seizures (Begg's rank correlation test: *p* = 0.63 and Egger's linear regression test: 0.199). The certainty of evidence was judged as high for thrombotic events but moderate for seizures due to imprecision (Table 2).

**Table 2:** GRADE evidence profile: TXA for bleeding patients

Certainty assessment							No. of patients		Effect		Certainty	Importance
No. of studies	Study design	Risk of bias	Inconsistency	Indirectness	Imprecision	Other considerations	TXA	Control	Relative (95% CI)	Absolute (95% CI)		
Thrombotic events												
230	Randomized trials	Not serious	Not serious	Not serious	Not serious	None	1342/52103 (2.5%)	1278/50211 (2.5%)	RR 1.00 (0.93 to 1.08)	0 fewer per 1000 (from 2 fewer to 2 more)	⨁⨁⨁⨁ HIGH	CRITICAL
Seizures												
40	Randomized trials	Not serious	Not serious^b^	Not serious	Serious^a^	None	404/30351 (1.3%)	351/29847 (1.1%)	RR 1.18 (0.91 to 1.53)	2 more per 1000 (from 1 fewer to 6 more)	⨁⨁⨁◯ MODERATE	CRITICAL
Seizures (dose > 2 g/day)												
7	Randomized trials	Not serious	Not serious	Not serious	Not serious	None	55/8642 (0.6%)	24/8666 (0.3%)	RR 3.05 (1.01 to 9.20)	6 more per 1000 (from 0 fewer to 23 more)	⨁⨁⨁⨁ HIGH	CRITICAL
Seizures (dose ≤ 2 g/day)												
33	Randomized trials	Not serious	Not serious	Not serious	Not serious	None	349/21709 (1.6%)	327/21181 (1.5%)	RR 1.02 (0.88 to 1.19)	0 more per 1000 (from 2 fewer to 3 more)	⨁⨁⨁⨁ HIGH	CRITICAL

a. Confidence interval includes effects suggesting benefit as well as no effect (downgraded by 1 level for imprecision)

b. Although there observed no inconsistency (*I*^*2*^ = 34%), clinical heterogeneity was suspected and further analysis was performed according to TXA dose

GRADE, grading of recommendations, assessment, development and evaluation. TXA, tranexamic acid. RR, risk ratio. CI, confidence interval

### Sensitivity and subgroup analysis with thrombotic events

**Fig. 3:**
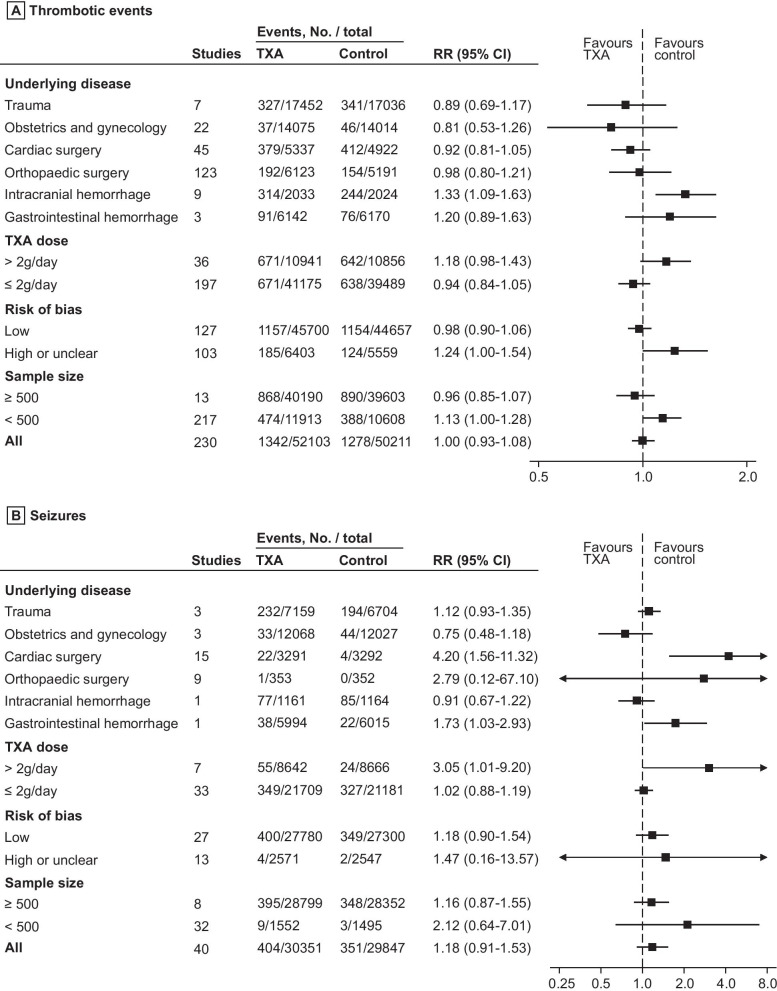
Forest plot of **A** thrombotic events and **B** seizures stratified subgroup analysis. TXA, tranexamic acid. RR, risk ratio. CI, confidence interval

**Fig. 4:**
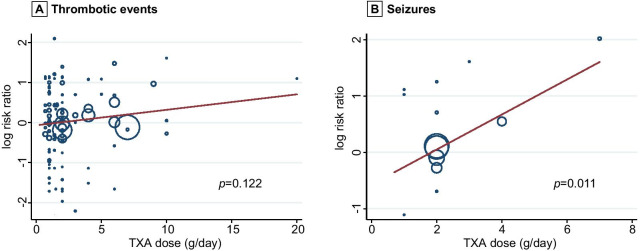
Meta-regression plot. Plots show the relation between the log risk ratio of **A** thrombotic events and **B** seizures with tranexamic acid and tranexamic acid dose (g/day). TXA, tranexamic acid

In sensitivity and subgroup analysis, we did not find any increase in thrombotic events in trauma (0.89 [0.69–1.17]), obstetrics and gynecology (0.81 [0.53–1.26]), cardiac surgery (0.92 [0.81–1.05]), orthopedic surgery (0.98 [0.80–1.21]) (Fig. 3A; Additional file 2: Figure S5). However, the risk of thrombotic events appeared higher in the TXA group than in the control group in spontaneous (non-traumatic) intracranial hemorrhage (1.33 [1.09–1.63]) (Fig. 3A). In a dose-by-dose sensitivity analysis, thrombotic events were not increased in patients receiving 2 g/day of TXA or less (≤ 2 g/day: 0.94 [0.84–1.05]) (Fig. 3A; Additional file 2: Figure S6). There was uncertainty on thrombotic events in patients receiving more than 2 g/day of TXA (> 2 g/day: 1.18 [0.98–1.43]). Meta-regression analysis showed there was no statistically significant association between higher dose of TXA and increased risk of thrombotic events (*p* = 0.122) (Fig. 4A). The results were similar to primary analysis, when the analyses were restricted to trials with low risk of bias (0.98 [0.90–1.06]) and sample size over 500 (0.96 [0.85–1.07]) (Fig. 3A). Of trials conducted in children, one patient had a thrombotic event in the TXA group, compared to none in the placebo group (Additional file 2: Figure S7).

### Sensitivity and subgroup analysis with seizures

In a per disease analysis, the risk of seizures was higher in the TXA group than in the control group in cardiac surgery (4.20 [1.56–11.32]) and gastrointestinal hemorrhage (1.73 [1.03–2.93]) (Fig. 3B; Additional file 2: Figure S8). In a dose-by-dose analysis, there was an observed increase in seizures in patients receiving more than 2 g/day of TXA (> 2 g/day: 3.05 [1.01–9.20]). Meanwhile, we found no increase in seizures in patients receiving 2 g/day of TXA or less (≤ 2 g/day: 1.02 [0.88–1.19]) (Fig. 3B; Additional file 2: Figure S9). Meta-regression analysis showed relative increase for seizures to be proportional to the magnitude of the TXA dose increase (*p* = 0.011) (Fig. 4B). There were no seizures reported in trials in children (Additional file 2: Figure S10).

### Venous thromboembolism, acute coronary syndrome and stroke stratified by underlying disease

**Fig. 5:**
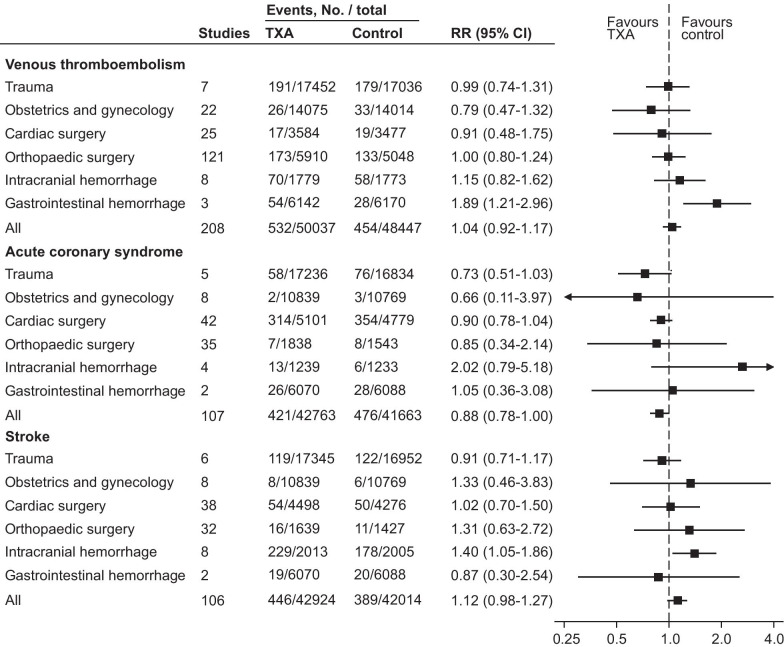
Forest plot of venous thromboembolism, acute coronary syndrome and stroke: subgroup analysis by underlying disease. TXA, tranexamic acid. RR, risk ratio. CI, confidence interval

We also performed sensitivity analysis for separately evaluating venous thromboembolism, acute coronary syndrome and stroke stratified by underlying disease (Fig. 5). The risk of venous thromboembolism was higher in TXA group than control group in gastrointestinal hemorrhage (1.89 [1.21–2.96]) (Fig. 5; Additional file 2: Figure S11). In other study populations, we found no increase in venous thromboembolism. We found no evidence TXA increased the risk of acute coronary syndrome in trauma, obstetrics and gynecology, cardiac surgery, orthopedic surgery and gastrointestinal hemorrhage (Fig. 5; Additional file 2: Figure S12). The risk of stroke appeared higher in the TXA group than in the control group in patients with intracranial hemorrhage (1.40 [1.05–1.86]) (Fig. 5; Additional file 2: Figure S13).

## Discussion

### Summary of main results

In this systematic review and meta-analysis, intravenous tranexamic acid administration did not appear to increase the risk of thrombotic events and seizures in bleeding patients. In per disease analysis, the risk of thrombotic events and stroke was higher in the TXA group than control in intracranial hemorrhage. With regard to seizures, its risk was increased in cardiac surgery and gastrointestinal hemorrhage, and those increase probably came from higher dose of TXA use. In view of the evidence, tranexamic can be safely used in bleeding patients, especially in trauma, obstetrics and gynecology and orthopedic surgery. There may be a dose-dependent increase in the risk of seizures.

### Mechanism of TXA on thrombotic events and seizures

Although theoretically, TXA might be expected to increase the risk of thrombotic events due to its antifibrinolytic activity, the present analysis found no evidence in support of this hypothesis. In the early phase of bleeding in trauma or surgery, early fibrinolysis is triggered by the release of tissue plasminogen activator and causes excessive bleeding [28–31]. Subsequently, plasminogen activator inhibitor-1 level increases and inhibit fibrinolysis [29, 32]. TXA is targeted to modulate early fibrinolysis, and thus it is rational we could not find any increase in thrombotic events in bleeding patients with hyperfibrinolysis even for TXA use.

TXA-associated seizures are caused by another mechanism. TXA is a competitive antagonist of GABA_A_ in brain. In in vitro and animal experiment, higher TXA level in the cerebral spinal fluid, correlated with serum concentration, was associated with the incidence of seizures [11, 33]. In fact, the results of this meta-analysis were consistent with the basic research reporting dose-dependent increase in the risk of seizures.

### Strength of the review

A major strength of this analysis is that the present meta-analysis included recently published all the available large-scale randomized controlled trials [5, 6, 12, 18–22]. Pooling data from 102,681 patients enrolled in 234 randomized controlled trials of tranexamic acid, our study included by far more information than any previous meta-analysis that have addressed this issue [4, 7, 13]. Therefore, this meta-analysis would provide greater evidence regarding safety of tranexamic acid in bleeding populations. In fact, the certainty of evidence evaluated by the GRADE approach was high in thrombotic events with narrow confidence intervals.

### Limitation of the review

However, this meta-analysis also has some limitations. First, all bleeding patients were included in this meta-analysis. Although this significantly increased the pooled information size, it also introduced clinical heterogeneity. However, we performed detailed pre-planned subgroup analysis according to disease type and dose of TXA use and some implication was attained with subgroup analysis. Second, although thrombotic events and seizures did not show any funnel plot asymmetry, there remains a possibility of reporting bias or publication bias due to inadequate information of adverse events in some trials. However, recently published large-scale randomized clinical trials that accounted for most of the weight in this meta-analysis provided detailed data of adverse events. Third, there might be heterogeneity in the diagnostic accuracy for detecting thrombosis adverse in the included trials and any inaccuracy may have biased toward the null of the TXA effects on the outcomes. Fourth, survivor bias may have influenced the results because patients who die are not ‘available’ to suffer a thrombotic adverse event. Although this possibility has not been quantified in individual randomized trials, we can consider the direction of any such bias. Because there were fewer deaths with TXA [5, 6], there would be less opportunity to have a thrombotic event among TXA treated patients. Therefore, our conclusions about safety would not be threatened by survivor bias. Fifth, since there were no available data regarding thrombotic events by anticoagulated status, we could not assess the use of antiplatelet/anticoagulant drugs, which are commonly used in perioperative period and ICU settings, and that could have affected the incidence of thrombosis.

### Agreements and disagreements with other studies or reviews

In the previous cumulative systematic review and meta-analysis including surgical bleeding, there was no observed increase in thromboembolic events such as stroke, ACS and VTE [4], although harmful effects were undeniable due to imprecision. Moreover, recent individual patient-data meta-analysis including two large-scale RCTs [5, 6] reported that vascular occlusive events did not increase in acute severe bleeding [7], considered as beneficial population for TXA treatment [34]. As well as the previous reports, the present study showed that TXA did not appear to increase thrombotic events in bleeding populations with high certainty of evidence. On the other hand, the risk of thrombotic events, especially stroke, were higher in TXA group than control group in intracranial (non-traumatic) hemorrhage, which was consistent with previous systematic review and meta-analysis [35, 36]. With regard to the thrombotic risk in females, tranexamic acid did not appear to increase the risk of thrombotic events in patients in obstetrics and gynecology trials.

As for seizures, administration of high dose TXA was associated with increased risk of seizures in cardiac surgery according to previous studies [37, 38]. A recently published large-scale randomized controlled trial (HALT-IT) comparing high dose of TXA (4 g/day) and placebo in gastrointestinal hemorrhage also showed increased risk of seizures. With totaling this evidence, the present study demonstrated dose-dependent increase in seizures with TXA use in bleeding patients. However, TXA did not appear to increase the risk of seizures when administered at a dose of 2 g/day or less.

## Conclusion

In conclusion, there is no evidence that tranexamic acid increases the risk of thrombotic events and seizures, and thus can be safely used in bleeding patients. However, because there may be dose-dependent increase in the risk of seizures, the administration of high doses should be avoided.

## Supplementary Information

**Additional file 1**. **Table S1** Search terms and results. **Table S2** Characteristics of included randomized controlled trials.

**Additional file 2**. **Figure S1** Risk of bias item presented as percentages. **Figure S2** Risk of bias summary and graph. ** Figure S3** Funnel plot of the thrombotic events. **Figure S4 **Funnel plot of the seizures. **Figure S5** Forest plot of the thrombotic events: subgroup analysis by underlying disease. **Figure S6** Forest plot of the thrombotic events: subgroup analysis by TXA dose. **Figure S7** Forest plot of the thrombotic events: subgroup analysis in children. **Figure S8** Forest plot of the seizures: subgroup analysis by underlying disease. **Figure S9** Forest plot of the seizures: subgroup analysis by TXA dose. **Figure S10** Forest plot of the seizures: subgroup analysis in children. **Figure S11** Forest plot of the venous thromboembolism: subgroup analysis by underlying disease. **Figure S12** Forest plot of the acute coronary syndrome: subgroup analysis by underlying disease. **Figure S13** Forest plot of the stroke: subgroup analysis by underlying disease severity.

## Data Availability

All data associated with this manuscript are included in the main text and supplementary materials.

## Abbreviations

TXA: Tranexamic acid
GRADE: Grading of recommendations, assessment, development and evaluations
GABA_A_: Gamma-aminobutyric acid receptor type A
PRISMA: Preferred Reporting Items for Systematic Reviews and Meta-Analyses
ACS: Acute coronary syndrome
TIA: Transient ischemic attack
VTE: Venous thromboembolism

## References

[1] Okamoto S, Okamoto U (1962). Amino-methyl-cyclohexane-carboxylic acid: AMCHA. A new potent inhibitor of the fibrinolysis. Keio J Med.

[2] Kobayashi T, Sugiura J (1966). The effect of a new potent antifibrinolytic agent, tranexamic acid. J Jpn Obstet Gynecol Soc.

[3] Okamoto S, Hijikata-Okunomiya A, Wanaka K, Okada Y, Okamoto U (1997). Enzyme-controlling medicines: introduction. Semin Thromb Hemost.

[4] Ker K, Edwards P, Perel P, Shakur H, Roberts I. Effect of tranexamic acid on surgical bleeding: systematic review and cumulative meta-analysis. BMJ. 2012;17;344:e3054. 10.1136/bmj.e3054 PMC3356857 22611164

[5] CRASH-2 trial collaborators, Shakur H, Roberts I, et al. Effects of tranexamic acid on death, vascular occlusive events, and blood transfusion in trauma patients with significant haemorrhage (CRASH-2): a randomised, placebo-controlled trial. Lancet. 2010;3;376(9734):23–32. 10.1016/S0140-6736(10)60835-5 20554319

[6] WOMAN Trial Collaborators (2017). Effect of early tranexamic acid administration on mortality, hysterectomy, and other morbidities in women with post-partum haemorrhage (WOMAN): an international, randomised, double-blind, placebo-controlled trial. Lancet.

[7] Gayet-Ageron A, Prieto-Merino D, Ker K (2018). Effect of treatment delay on the effectiveness and safety of antifibrinolytics in acute severe haemorrhage: a meta-analysis of individual patient-level data from 40138 bleeding patients. Lancet.

[8] Sperzel M, Huetter J (2007). Evaluation of aprotinin and tranexamic acid in different in vitro and in vivo models of fibrinolysis, coagulation and thrombus formation. J Thromb Haemost.

[9] McCormack PL (2012). Tranexamic acid: a review of its use in the treatment of hyperfibrinolysis. Drugs.

[10] Manji RA, Grocott HP, Leake J (2012). Seizures following cardiac surgery: the impact of tranexamic acid and other risk factors. Can J Anaesth.

[11] Lecker I, Wang DS, Whissell PD, Avramescu S, Mazer CD, Orser BA (2016). Tranexamic acid-associated seizures: causes and treatment. Ann Neurol.

[12] HALT-IT Trial Collaborators (2020). Effects of a high-dose 24-h infusion of tranexamic acid on death and thromboembolic events in patients with acute gastrointestinal bleeding (HALT-IT): an international randomised, double-blind, placebo-controlled trial. Lancet.

[13] Lin Z, Xiaoyi Z (2016). Tranexamic acid-associated seizures: a meta-analysis. Seizure.

[14] Liberati A, Altman DG, Tetzlaff J, et al. The PRISMA statement for reporting systematic reviews and meta-analyses of studies that evaluate healthcare interventions: explanation and elaboration. BMJ (Clin Res Ed). 2009;339:b2700. 10.1136/bmj.b2700 PMC2714672 19622552

[15] Murao S, Nakata H, Yamakawa K (2020). Safety of tranexamic acid in thrombotic adverse events and seizure in patients with haemorrhage: a protocol for a systematic review and meta-analysis. BMJ Open.

[16] Higgins JPT, Thomas J, Chandler J, et al. Cochrane Handbook for Systematic Reviews of Interventions version 6.1 (updated September 2020). Cochrane, 2020. Available from www.training.cochrane.org/handbook.

[17] Jakobsen JC, Wetterslev J, Winkel P (2014). Thresholds for statistical and clinical significance in systematic reviews with meta-analytic methods. BMC Med Res Methodol.

[18] Myles PS, Smith JA, Forbes A (2017). Tranexamic acid in patients undergoing coronary-artery surgery. N Engl J Med.

[19] Sprigg N, Flaherty K, Appleton JP (2018). Tranexamic acid for hyperacute primary IntraCerebral Haemorrhage (TICH-2): an international randomised, placebo-controlled, phase 3 superiority trial. Lancet.

[20] CRASH-3 trial collaborators. Effects of tranexamic acid on death, disability, vascular occlusive events and other morbidities in patients with acute traumatic brain injury (CRASH-3): a randomised, placebo-controlled trial. Lancet. 2019;394(10210):1713–1723. 10.1016/S0140-6736(19)32233-0 PMC6853170 31623894

[21] Rowell SE, Meier EN, McKnight B (2020). Effect of out-of-hospital tranexamic acid vs placebo on 6-month functional neurologic outcomes in patients with moderate or severe traumatic brain injury. JAMA.

[22] Sentilhes L, Winer N, Azria E (2018). Tranexamic acid for the prevention of blood loss after vaginal delivery. N Engl J Med.

[23] Abdel-Aleem H, Alhusaini TK, Abdel-Aleem MA (2013). Effectiveness of tranexamic acid on blood loss in patients undergoing elective cesarean section: randomized clinical trial. J Matern Fetal Neonatal Med.

[24] Gungorduk K, Yıldırım G, Asıcıoğlu O, Gungorduk OC, Sudolmus S, Ark C (2011). Efficacy of intravenous tranexamic acid in reducing blood loss after elective cesarean section: a prospective, randomized, double-blind, placebo-controlled study. Am J Perinatol.

[25] Shi J, Ji H, Ren F (2013). Protective effects of tranexamic acid on clopidogrel before coronary artery bypass grafting: a multicenter randomized trial. JAMA Surg.

[26] Casati V, Bellotti F, Gerli C (2001). Tranexamic acid administration after cardiac surgery: A prospective, randomized, double-blind, placebo-controlled study. Anesthesiology.

[27] Hillman J, Fridriksson S, Nilsson O, Yu Z, Saveland H, Jakobsson KE (2002). Immediate administration of tranexamic acid and reduced incidence of early rebleeding after aneurysmal subarachnoid hemorrhage: a prospective randomized study. J Neurosurg.

[28] Chapman MP, Moore EE, Moore HB (2016). Overwhelming tPA release, not PAI-1 degradation, is responsible for hyperfibrinolysis in severely injured trauma patients. J Trauma Acute Care Surg.

[29] Wu X, Darlington DN, Cap AP (2016). Procoagulant and fibrinolytic activity after polytrauma in rat. Am J Physiol Regul Integr Comp Physiol.

[30] Kojima T, Gando S, Morimoto Y (2001). Systematic elucidation of effects of tranexamic acid on fibrinolysis and bleeding during and after cardiopulmonary bypass surgery. Thromb Res.

[31] Roberts I, Edwards P, Prieto D (2017). Tranexamic acid in bleeding trauma patients: an exploration of benefits and harms. Trials.

[32] Moore EE, Moore HB, Gonzalez E (2015). Postinjury fibrinolysis shutdown: rationale for selective tranexamic acid. J Trauma Acute Care Surg.

[33] Schlag MG, Hopf R, Zifko U (2002). Epileptic seizures following cortical application of fibrin sealants containing tranexamic acid in rats. Acta Neurochir (Wien).

[34] Coats TJ, Morsy M (2020). Biological mechanisms and individual variation in fibrinolysis after major trauma. Emerg Med J.

[35] Anker-Møller T, Troldborg A, Sunde N, Hvas AM (2017). Evidence for the use of tranexamic acid in subarachnoid and subdural hemorrhage: a systematic review. Semin Thromb Hemost.

[36] Baharoglu MI, Germans MR, Rinkel GJ, et al. Antifibrinolytic therapy for aneurysmal subarachnoid haemorrhage. Cochrane Database Syst Rev. 2013;(8):CD001245. 10.1002/14651858.CD001245.pub2 PMC8407182 23990381

[37] Kalavrouziotis D, Voisine P, Mohammadi S (2012). High-dose tranexamic acid is an independent predictor of early seizure after cardiopulmonary bypass. Ann Thorac Surg.

[38] Murkin JM, Falter F, Granton J (2010). High-dose tranexamic acid is associated with nonischemic clinical seizures in cardiac surgical patients. Anesth Analg.

