# Supplementary material for: Effect of tranexamic acid on thrombotic events and seizures in bleeding patients: a systematic review and meta-analysis
Source: Crit Care. 2021 Nov 1;25:380. doi: 10.1186/s13054-021-03799-9 (PMC8561958; doi:10.1186/s13054-021-03799-9)
Supplement: Supplementary file 1 — Additional file 1. Table S1 Search terms and results. Table S2 Characteristics of included randomized controlled trials. [file 13054_2021_3799_MOESM1_ESM.pdf]

Online Supplementary Table

Table of Contents

Table S1. Search terms and results. 2

Table S2. Characteristics of included randomized controlled trials 3

References 22

Table S1. Search terms and results.

Database	Dates searched	Search terms	Results found
MEDLINE (Pubmed)	Jun 1st 2020	#1: tranexamic acid[MeSH] OR tranexamic acid[tiab] OR TXA[tiab]	6177
		#2: randomized controlled	762960
		#3: controlled clinical trial [pt]	604335
		#4: placebo [tiab]	213661
		#5: drug therapy [sh]	2238246
		#6: randomly [tiab]	335230
		#7: trial [tiab]	598168
		#8: groups [tiab]	2081567
		#9: #2 OR #3 OR #4 OR #5 OR #6 OR #7 OR #8	4864342
		#10: animals [mh] NOT humans [mh]	4743556
		#11: #9 NOT #10	4230945
		#12: #1 AND #9	3039
Cochrane Central Register of Controlled Trials	Jun 1st 2020	#1: mh Tranexamic acid	1005
		#2: Tranexamic acid:ti,ab,kw	2709
		#3: TXA:ti,ab,kw	853
		#4: #1OR #2 OR #3	2657
		#5: #4, Limits; Trials:	2616
Embase	Jun 1st 2020	#1: EMB.EXACT.EXPLODE("tranexamic acid")	12781
		#2: TI,AB(tranexamic acid)	6496
		#3: TI,AB(TXA)	5919
		#4: #1 OR #2 OR #3	17658
		#5: 'crossover procedure':de OR 'double-blind procedure':de OR 'randomized controlled trial':de OR 'single-blind procedure':de OR (random* OR factorial* OR crossover* OR cross NEXT/1 over* OR placebo* OR doubl* NEAR/1 blind* OR singl* NEAR/1 blind* OR assign* OR allocat* OR volunteer*):de,ab,ti	2540998
		#6: (#4 and #5)	3451

Table S2. Characteristics of included randomized controlled trials

Source	Study characteristics	Population	No. of patients	Mean age (years) TXA/Control	Percentage of female TXA/Control	Percentage of anticoagulant/ antiplatelet TXA/Control	Intervention	
							TXA	Control
Abdel-Aleem 2013 [1]	Single center	Obstetrics and gynecology	740	26/16	100/100	NA	1g TXA bolus	No treatment
Abdul 2019 [2]	Single center	Obstetrics and gynecology	80	NA/35	100/100	NA	NA	Placebo
Aguilera 2013 [3]	Single center	Orthopaedic surgery	83	72/75	91/84	NA	1g TXA bolus followed by 1g bolus	No treatment
Aguilera 2015 [4]	Multicenter	Orthopaedic surgery	100	72/74	76/64	NA	1g TXA bolus followed by 1g bolus	No treatment
Ahmed 2014 [5]	Single center	Obstetrics and gynecology	124	29/27	100/100	NA	10mg/kg TXA bolus	No treatment
Ahn 2012 [6]	Single center	Cardiac surgery	76	69/67	40/53	100/100	1g TXA bolus followed 200mg/hr maintenance	Placebo
Akgul 2016 [7]	Single center	Orthopaedic surgery	70	NA	NA	NA	20mg/kg TXA bolus	No treatment
Alimian 2011[8]	Single center	Head and neck surgery	84	33/35	40/43	NA	10mg/kg TXA bolus	Placebo
Alizadeh 2015 [9]	Single center	Cardiac surgery	200	58/59	70/65	NA	10mg/kg TXA bolus followed by 0.5-2mg/kg/hr maintenance	Placebo
Altun 2017 [10]	Single center	Cardiac surgery	54	65/62	14/10	100/100	10mg/kg TXA bolus followed by 1mg/kg/hr maintenance	No treatment
Alvarez 2008 [11]	Single center	Orthopaedic surgery	95	71/72	85/80	NA	10mg/kg TXA bolus followed by 1mg/kg/hr maintenance	Placebo
Alvarez 2019 [12]	Single center	Orthopaedic surgery	44	69/71	73/73	NA	10mg/kg TXA bolus	Placebo

Andreasen 2004 [13]	Single center	Cardiac surgery	44	62/64	14/17	NA	1g TXA bolus followed by 200mg/hr maintenance until additional 1.5g	Placebo
Apipan 2018 [14]	Single center	Oral and maxillofacial surgery	80	26,25,26/25	80,75,60/60	NA	10mg/kg TXA bolus/ 15mg/kg TXA bolus/ 20mg/kg TXA bolus	Placebo
Armellin 2001 [15]	Single center	Cardiac surgery	283	66/66	50/36	13/7	2.5g TXA bolus followed by 2.5g bolus	Placebo
Auvinen 1987 [16]	Single center	Otorhinolaryngology	76	50/51	90/95	NA	0.5g TXA bolus followed by 0.5 g bolus during each 8 h until 24h	Placebo
Avvisati 1989 [17]	Multicenter	Hematologic	12	35/40	50/67	NA	1g TXA bolus	Placebo
Barrachina 2016 [18]	Multicenter	Orthopaedic surgery	108	69,62/68	54,44/43	NA	10mg/kg TXA bolus / 15mg/kg TXA bolus	Placebo
Baruah 2016 [19]	Single center	Orthopaedic surgery	60	58/55	20/17	NA	15mg/kg TXA bolus	Placebo
Batibay 2018 [20]	Single center	Orthopaedic surgery	70	36/35	NA	NA	10mg/kg TXA bolus	Placebo
Benoni 1996 [21]	Single center	Orthopaedic surgery	86	76/74	70/77	NA	10mg/kg TXA bolus followed by 10mg/kg bolus	Placebo
Benoni 2000 [22]	Single center	Orthopaedic surgery	40	70/68	70/42	NA	20mg/kg TXA bolus	Placebo
Benoni 2001 [23]	Single center	Orthopaedic surgery	38	66/68	50/50	NA	10mg/kg TXA bolus	Placebo
Bidolegui 2014 [24]	Single center	Orthopaedic surgery	50	72/72	60/80	NA	15mg/kg TXA bolus	Placebo
Boylan 1996 [25]	Single center	Hepatic surgery	45	50/49	NA	NA	40mg/kg/hr TXA continuous infusion (maximum 20g)	Placemo

Brown 1997 [26]	Single center	Cardiac surgery	90	62,62/59	20,17/20	100/100	15mg/kg TXA bolus followed by 1mg/kg/hr maintenance	Placebo
Bulutcu 2005 [27]	Single center	Cardiac surgery	50	4/4	40/50	NA	100mg/kg TXA bolus followed by 100mg/kg bolus and 100mg/kg bolus	No treatment
Caglar 2008 [28]	Single center	Obstetrics and gynecology	100	34/35	100/100	NA	10mg/kg TXA bolus followed by 1mg/kg/hr maintenance	Placebo
Cansancao 2018 [29]	Single center	Plastic surgery	20	40/36	NA	NA	20mg/kg TXA bolus	Placebo
Carabini 2017 [30]	Single center	Orthopaedic surgery	61	65/68	68/70	NA	10mg/kg TXA bolus followed by 1mg/kg/hr maintenance	Placebo
Casati 2001 [31]	Single center	Cardiac surgery	510	64,61/64	40,31/36	32/32	1mg/kg/hr TXA continuous infusion / 2mg/kg/hr TXA continuous infusion for 12hr	Placebo
Casati 2002 [32]	Single enter	Cardiac surgery	60	59/63	21/34	41/38	400mg/hr TXA continuous infusion followed by 500mg bolus	Placebo
Casati 2004 [33]	Single enter	Cardiac surgery	102	64/61	15/16	38/44	1g TXA bolus followed 400mg/hr maintenance	Placebo
Chakravarthy 2012 [34]	Single center	Cardiac surgery	50	58/60	20/14	NA	10mg/kg TXA bolus followed by 1mg/kg/hr maintenance	Placebo
Chakroun-Walha 2019 [35]	Single center	Trauma	180	44/39	11/8	NA	1g TXA bolus followed by 1g maintenance over 8hr	Placebo
Chandra 1978 [36]	Single center	Intracranial hemorrhage	39	NA	45/47	NA	1g TXA bolus followed by 1g bolus every 4hr	Placebo

Chareancholvanich 2012[37]	Single center	Orthopaedic surgery	240	69/68	86/86	NA	10mg/kg TXA bolus followed by 10mg/kg bolus	Placebo
Charoencholvanich 2011[38]	Single center	Orthopaedic surgery	100	69/69	86/84	NA	10mg/kg TXA bolus followed by 10mg/kg bolus and orally for 5 days	Placebo
Chauhan 2003[39]	Single center	Cardiac surgery	120	4/4	16/26	NA	10mg/kg TXA bolus followed by 10mg/kg bolus and 10mg/kg bolus	No treatment
Chauhan 2004[40]	Single center	Cardiac surgery	100	4/4	28/30	NA	10mg/kg TXA bolus followed by 10mg/kg bolus and 10mg/kg bolus	No treatment
Chen 2016[41]	Single center	Orthopaedic surgery	120	67/64	73/77	NA	1g TXA bolus	Placebo
Chen 2019[42]	Single center	Orthopaedic surgery	176	77/77	56/58	NA	15mg/kg TXA bolus followed by 15mg/kg bolus and 15mg/kg bolus	Placebo
Chin 2020[43]	Single center	Orthopaedic surgery	81	NA	NA	NA	1g TXA bolus	Placebo
Choi 2009[44]	Single center	Oral and maxillofacial surgery	61	24/23	31/38	NA	20mg/kg TXA bolus	Placebo
Christabel 2014[45]	Single center	Oral and maxillofacial surgery	49	24/23	NA/62	NA	10mg/kg TXA bolus	Placebo
Claeys 2007[46]	Single center	Orthopaedic surgery	40	73/68	75/65	7/4	15mg/kg TXA bolus	Placebo
Clave 2019[47]	Multicenter	Orthopaedic surgery	229	65,67/64	60,56/56	NA	1g TXA bolus followed by 1g bolus /1g TXA bolus followed by 1g bolus at postoperative hour 3, 7, 11	Placebo
Cohen 2020[48]	Single center	Plastic surgery	44	59/62	100/100	NA	1g TXA bolus by 1g bolus	Placebo

Colomina 2016 [49]	Multicenter	Orthopaedic surgery	95	59/61	66/73	NA	10mg/kg TXA bolus followed by 2mg/kg/hr maintenance	Placebo
Crescenti 2011 [50]	Single center	Urological	200	64/64	0/0	17/10	500mg TXA bolus followed by 200mghr maintenance	Placebo
Cvetanovich 2018 [51]	Single center	Orthopaedic surgery	108	68/65	48/50	NA	1g TXA bolus	Placebo
Dadure 2011 [52]	Single center	Oral and maxillofacial surgery	39	0.5/0.5	11/15	NA	10mg/kg TXA bolus followed by 1mg/kg/hr maintenance	Placebo
Diprose 2005 [53]	Single center	Cardiac surgery	123	65/65	21/16	NA	5g TXA bolus	Placebo
Ducloy-Bouthors 2011 [54]	Multicenter	Obstetrics and gynecology	144	29/28	100/100	NA	4g TXA infusion over an hour followed by 1g/hr maintenance	Placebo
Ebrahimi 2019 [55]	Single center	Trauma	80	32/32	18/13	NA	1g TXA bolus followed by 1g maintenance over 8hr	Placebo
Ekback 2000 [56]	Single center	Orthopaedic surgery	40	66/66	55/45	NA	10mg/kg TXA bolus followed by 10mg/kg bolus and 1mg/kg/hr maintenance	Placebo
Eldaba 2013 [57]	Single center	Otorhinolaryngology	100	7/7	59/66	NA	25mg/kg TXA bolus	Placebo
Elwatidy 2008 [58]	Single center	Orthopaedic surgery	64	52/50	34/44	NA	2g TXA bolus followed by 100mg/hr maintenance	Placebo
Emara 2014 [59]	Single center	Orthopaedic surgery	40	57/56	40/30	NA	10mg/kg TXA bolus followed by 5mg/kg/hr maintenance	Placebo
Engel 2001 [60]	Single center	Orthopaedic surgery	24	71/66	67/67	NA	15mg/kg TXA bolus followed by 10mg/kg maintenance	No treatment
Engqvist 1979 [61]	Single center	Gastrointestinal	149	59/56	27/16	NA	1g TXA bolus followed by	Placebo

		hemorrhage					1g bolus every 4hr	
Esfandiari 2012 [62]	Single center	Cardiac surgery	150	54/55	NA	NA	10mg/kg TXA bolus followed by 1mg/kg bolus	Placebo
Ezhevskaya 2018 [63]	Single center	Orthopaedic surgery	160	15/16	88/93	NA	15mg/kg TXA bolus followed by 2mg/kg/hr maintenance	Placebo
Faraoni 2014 [64]	Single center	Cardiac surgery	36	71,61/62	NA	NA	30mg/kg TXA bolus followed by 16mg/kg/hr maintenance/ 5mg/kg TXA bolus followed by 5mg/kg/hr maintenance	Placebo
Farrokhi 2011 [65]	Single center	Orthopaedic surgery	76	46/51	82/71	NA	10mg/kg TXA bolus followed by 1mg/kg/hr maintenance	Placebo
Felli 2019 [66]	Single center	Orthopaedic surgery	80	31/32	33/10	NA	15mg/kg TXA bolus	Placebo
Fenger-Eriksen 2019 [67]	Single center	Oral and maxillofacial surgery	30	8/7	NA	NA	10ml/kg TXA bolus followed by 3ml/kg/hr maintenance	Placebo
Fodstad 1981 [68]	Single center	Intracranial hemorrhage	59	50/53	57/59	NA	1g TXA bolus followed by 1g bolus every 4hr during the 1st and 2nd week.	Placebo
Fraval 2016 [69]	Single center	Orthopaedic surgery	101	60/60	36/45	NA	15mg/kg TXA bolus followed by 15mg/kg bolus and 15mg/kg bolus	Placebo
Gai 2004 [70]	Multicenter	Obstetrics and gynecology	180	30/30	100/100	NA	1g TXA bolus	No treatment
Garg 2012 [71]	Single center	Orthopaedic surgery	52	NA	NA	NA	15mg/kg TXA bolus	Placebo
Garneti 2004 [72]	Single center	Orthopaedic surgery	50	70/68	NA	NA	10mg/kg TXA bolus	Placebo

Goobie 2011 [73]	Single center	Neurosurgery	43	23/25	35/45	NA	50mg/kg TXA bolus followed by 5mg/kg/hr maintenance	Placebo
Goobie 2018 [74]	Single center	Orthopaedic surgery	111	15/15	18/24	NA	50mg/kg TXA bolus followed by 10mg/kg/hr maintenance	Placebo
Good 2003 [75]	Single center	Orthopaedic surgery	51	72/72	67/75	NA	10mg/kg TXA bolus	Placebo
Goswami 2013 [76]	Single center	Obstetrics and gynecology	90	23/24	100/100	NA	10mg/kg TXA bolus/ 15mg/kg TXA bolus	Placebo
Greiff 2012 [77]	Single center	Cardiac surgery	63	77/77	40/42	100/100	10mg/kg TXA bolus followed by 1mg/kg/hr maintenance	Placebo
Gungorduk 2011 [78]	Single center	Obstetrics and gynecology	660	27/26	100/100	NA	1g TXA bolus	Placebo
Gungorduk 2013 [79]	Single center	Obstetrics and gynecology	439	28/28	100/100	NA	1g TXA bolus	Placebo
Gupta 2012 [80]	Single center	Obstetrics and gynecology	60	46/48	100/100	NA	1g TXA bolus	Placebo
Hardy 1998 [81]	Single center	Cardiac surgery	88	NA	37/16	NA	10g TXA bolus	Placebo
Helito 2019 [82]	Single center	Orthopaedic surgery	60	NA	NA	NA	10mg/kg TXA bolus followed by 10mg/kg bolus	No treatment
Hiippala 1995 [83]	Single center	Orthopaedic surgery	28	70/70	87/77	NA	15mg/kg TXA bolus	Placebo
Hiippala 1997 [84]	Single center	Orthopaedic surgery	77	70/69	90/79	NA	15mg/kg TXA bolus followed by 10mg/kg bolus and 10mg/kg bolus	Placebo
Hillman 2002 [85]	Single center	Intracranial hemorrhage	504	NA	65/65	NA	1g TXA bolus followed by 1g bolus every 6hr	No treatment
Hooda 2015 [86]	Single center	Neurosurgery	60	39/42	53/77	NA	20mg/kg TXA bolus followed by 1mg/kg/hr maintenance	Placebo

Horrow 1991 [87]	Single center	Cardiac surgery	81	65/64	NA	100/100	10mg/kg TXA bolus followed by 1mg/kg/hr maintenance	Placebo
Horrow 1995 [88]	Single center	Cardiac surgery	148	67,61,66,63, 65/63	25,14,14,19, 22/15	100/100	6 groups ranged from quarter 2.5mg/kg TXA bolus followed by 0.25mg/kg/hr maintenance to twice 20mg/kg TXA bolus followed by 2mg/kg/hr maintenance	Placebo
Hsu 2015 [89]	Single center	Orthopaedic surgery	60	57/60	63/50	NA	1g TXA bolus followed by 1g bolus	Placebo
Husted 2003 [90]	Single center	Orthopaedic surgery	40	65/67	65/70	NA	10mg/kg TXA bolus followed by 1mg/kg/hr maintenance	Placebo
Jansen 1999 [91]	Single center	Orthopaedic surgery	42	71/71	76/86	NA	15mg/kg TXA bolus followed by 15mg/kg bolus every 8hr	Placebo
Jares 2003 [92]	Single center	Cardiac surgery	47	NA	31/20	100/100	1g TXA bolus followed by 200mg/hr maintenance	No treatment
Jaszczyk 2015 [93]	Single center	Orthopaedic surgery	124	65/66	51/49	NA	15mg/kg TXA bolus	Placebo
Johansson 2005 [94]	Multicenter	Orthopaedic surgery	100	69/68	47/47	NA	15mg/kg TXA bolus	Placebo
Jokar 2017 [95]	Single center	Trauma	80	35/36	10/15	NA	1g TXA bolus followed by 1g maintenance over 8hr	Placebo
Kakar 2009 [96]	Single center	Orthopaedic surgery	50	63/67	72/72	NA	10mg/kg TXA bolus followed by 1mg/kg/hr maintenance	Placebo
Karaaslan 2015 [97]	Single center	Orthopaedic surgery	105	28/28	4/6	NA	15mg/kg TXA bolus followed by 10mg/kg/hr maintenance	Placebo

Karski 1995 [98]	Single center	Cardiac surgery	147	59,63/58	NA	100/100	10g TXA bolus/ 10g TXA bolus followed by another 10g maintenance over 5hr	Placebo
Karski 2005 [99]	Single center	Cardiac surgery	312	60/60	13/11	100/100	100mg/kg TXA bolus	Placebo
Kaste 1979 [100]	Single center	Intracranial hemorrhage	64	NA	50/56	NA	1g TXA bolus followed by 1g bolus every 6hr	Placebo
Katsaros 1996 [101]	Single center	Cardiac surgery	210	65/63	35/25	NA	10g TXA bolus	Placebo
Kazemi 2010 [102]	Single center	Orthopaedic surgery	64	47/45	28/38	NA	15mg/kg TXA bolus	Placebo
Keyhani 2015 [103]	Single center	Orthopaedic surgery	80	68/64	35/53	NA	0.5g TXA bolus	Placebo
Kim 2014 [104]	Single center	Orthopaedic surgery	180	74/72	88/87	NA	10mg/kg TXA bolus followed by 10mg/kg bolus	No treatment
Kim 2017 [105]	Single center	Orthopaedic surgery	72	63,61/65	33,50/63	NA	5mg/kg TXA bolus followed by 1mg/kg/hr maintenance/ 10mg/kg TXA bolus followed by 2mg/kg/hr maintenance	Placebo
Kimura 2019 [106]	Single center	Orthopaedic surgery	256	55/55	46/48	NA	15mg/kg TXA bolus	Placebo
Kuitunen 2005 [107]	Single center	Cardiac surgery	40	63/65	NA	100/100	15mg/kg TXA bolus followed by 15mg/kg bolus and 15mg/kg bolus	Placebo
Kulkarni 2016 [108]	Single center	Head and neck surgery	240	51/51	25/21	NA	10mg/kg TXA bolus	Placebo
Kundu 2015 [109]	Single center	Orthopaedic surgery	60	60/60	73/77	NA	20mg/kg TXA bolus	Placebo
Lack 2017 [110]	Multicenter	Orthopaedic surgery	88	42/40	16/16	NA	10mg/kg TXA bolus followed by 10mg/kg bolus	Placebo

Later 2009 [111]	Single center	Cardiac surgery	202	64/65	26/35	74/67	1g TXA bolus followed by 0.5g bolus and 400mg/hr maintenance	Placebo
Lee 2012 [112]	Single center	Orthopaedic surgery	72	70/69	86/86	NA	10mg/kg TXA bolus followed by 10mg/kg bolus	Placebo
Lee 2013 [113]	Single center	Orthopaedic surgery	68	53/51	41/35	NA	15mg/kg TXA bolus followed by 15mg/kg continuous infusion	Placebo
Lei 2020 [114]	Single center	Orthopaedic surgery	200	66,67,66/65	86,82,80/80	NA	20mg/kg TXA bolus followed by 3 doses of 1g bolus/ followed by 4 doses of 1g/ followed by 5 doses of 1g	No treatment
Lemay 2004 [115]	Single center	Orthopaedic surgery	39	60/54	40/32	NA	10mg/kg TXA bolus followed by 1mg/kg/hr maintenance	Placebo
Lin 2012 [116]	Single center	Orthopaedic surgery	151	71,70/70	41,43/43	NA	10mg/kg TXA bolus/ 20mg/kg TXA bolus	Placebo
Liu 2017 [117]	Single center	Orthopaedic surgery	224	64,63/62	55,60/58	NA	10mg/kg TXA bolus/ 15mg/kg TXA bolus	Placebo
Liu 2019 [118]	Single center	Orthopaedic surgery	72	59/60	49/51	NA	1g TXA bolus	Placebo
Lundin 2014 [119]	Multicenter	Obstetrics and gynecology	100	63/65	NA	14/10	15mg/kg TXA bolus	Placebo
Luo 2019 [120]	Single center	Orthopaedic surgery	90	75/76	48/57	NA	15mg/kg TXA bolus followed by 15mg/kg bolus	Placebo
Ma 2019 [121]	Single center	Orthopaedic surgery	124	61/61	58/48	NA	15mg/kg TXA bolus	No treatment
MacGillivray 2011 [122]	Single center	Orthopaedic surgery	60	62,65/66	65,60/75	NA	10mg/kg TXA bolus/ 15mg/kg TXA bolus	Placebo

Maddali 2007 [123]	Single center	Cardiac surgery	222	57/58	28/35	100/100	10mg/kg TXA bolus followed by 1mg/kg/hr maintenance	Placebo
Maged 2015 [124]	Single center	Obstetrics and gynecology	200	25/25	100/100	NA	1g TXA bolus	Placebo
Mahmood 2017 [125]	Single center	Orthopaedic surgery	173	66/65	49/63	NA	1g TXA bolus	No treatment
Maniar 2012 [126]	Single center	Orthopaedic surgery	200	67,68,67,67/ 66	75,72,80,82/ 90	NA	10mg/kg TXA bolus/ 10mg/kg TXA bolus followed by 10mg/kg bolus/ 10mg/kg TXA bolus followed by 10mg/kg bolus and 10mg/kg bolus	No treatment
Mansouri 2012 [127]	Single center	Cardiac surgery	60	48/48	NA	100/100	30mg/kg TXA bolus followed by 1mg/kg maintenance	Placebo
Mehr-Aein 2007 [128]	Single center	Cardiac surgery	66	44/45	8/6	90/94	15mg/kg TXA bolus followed by 15mg/kg bolus	Placebo
Meng 2018 [129]	Single center	Urology	60	71/71	NA	NA	1g TXA bolus	Placebo
Misfeld 1998 [130]	Single center	Cardiac surgery	28	56/59	21/0	100/100	10mg/kg TXA bolus followed by 1mg/kg/hr maintenance	No treatment
Molloy 2005 [131]	Single center	Orthopaedic surgery	100	NA	NA	NA	0.5g TXA bolus followed by 0.5g bolus	No treatment
Monaco 2019 [132]	Single center	Cardiac surgery	100	69/71	14/0	62/70	0.5 TXA bolus followed by 250mg/hr maintenance	Placebo
Motififard 2015 [133]	Single center	Orthopaedic surgery	90	67/66	78/71	NA	0.5g TXA bolus followed by 0.5g bolus	Placebo
Mu 2018 [134]	Single center	Orthopaedic surgery	87	54/53	40/45	NA	15mg/kg TXA bolus followed by 1mg/kg maintenance	Placebo
Myles 2016 [135]	Multi center	Cardiac surgery	4631	67/67	16/17	83/84	100mg TXA bolus /50mg TXA bolus after 1392 had been enrolled	Placebo

Na 2015 [136]	Single center	Orthopaedic surgery	55	50/53	31/38	NA	10mg/kg TXA bolus	Placebo
Nagabhushan 2018 [137]	Single center	Orthopaedic surgery	50	50/52	36/44	NA	15mg/kg TXA bolus followed by 1mg/kg maintenance	Placebo
Neilipovitz 2011 [138]	Single center	Orthopaedic surgery	40	14/14	45/72	NA	10mg/kg TXA bolus followed by 1mg/kg maintenance	Placebo
Nejad 2011 [139]	Single center	Cardiac surgery	100	55/54	18/30	100/100	1g TXA bolus followed by 1g bolus	Placebo
Niskanen 2005 [140]	Single center	Orthopaedic surgery	39	66/65	68/65	NA	10mg/kg TXA bolus	Placebo
Nugent 2017 [141]	Single center	Orthopaedic surgery	41	48/53	33/22	NA	1g TXA bolus	Placebo
Oremus 2013 [142]	Single center	Orthopaedic surgery	98	69/69	16/24	14/10	1g TXA bolus followed by 1g bolus	Placebo
Orpen 2006 [143]	Single center	Orthopaedic surgery	29	73/69	47/79	NA	15mg/kg TXA bolus	Placebo
Painter 2018 [144]	Multicenter	Orthopaedic surgery	140	69/68	61/48	9/12	15mg/kg TXA bolus followed by 15mg/kg bolus	Placebo
Palija 2020 [145]	Single center	Orthopaedic surgery	80	63/61	52/60	NA	1g TXA bolus followed by 1g bolus	Placebo
Peters 2015 [146]	Single center	Orthopaedic surgery	32	60/43	NA	NA	10mg/kg TXA bolus followed by 1mg/kg maintenance	Placebo
Pleym 2003 [147]	Single center	Cardiac surgery	80	64/62	15/18	100/100	30mg/kg TXA bolus	Placebo
Prakash 2017 [148]	Single center	Orthopaedic surgery	100	70/68	NA	NA	30mg/kg TXA bolus	Placebo
Prasad 2020 [149]	Single center	Abdominal surgery	60	46,47/49	75.60/75	NA	10mg/kg TXA bolus/ 10mg/kg TXA bolus followed by 1mg/kg maintenance	Placebo

Raksakietisak 2015 [150]	Single center	Orthopaedic surgery	78	53/53	59/69	NA	10mg/kg TXA bolus followed by 10mg/kg bolus	Placebo
Ramezani 2005 [151]	Single center	Ophthalmology	62	57/50	41/47	NA	10mg/kg TXA bolus followed by 10mg/kg bolus	No treatment
Ray 2016 [152]	Single center	Obstetrics and gynecology	100	25/26	100/100	NA	1g TXA bolus	Placebo
Reid 1997 [153]	Single center	Cardiac surgery	41	3/3	NA	100/100	100mg/kg TXA bolus followed by 10mg/kg maintenance	Placebo
Roberts 2019 [154]	Multicenter	Trauma	12737	42/42	19/20	NA	1g TXA bolus followed by 1g infusion over 8hr	Placebo
Roberts 2020 [155]	Multicenter	Gastrointestinal hemorrhage	12009	58/58	36/35	NA	1g TXA bolus followed by 3g infusion over 8hr	Placebo
Ross 2000 [156]	Multicenter	Intracranial hemorrhage	462	55/56	61/69	NA	1g TXA bolus every 4hr in the first week	placebo
Rowell 2020 [157]	Multicenter	Trauma	966	39,40/36	27,26/25	NA	2g TXA bolus/ 1g TXA bolus followed by 1g infusion over 8hr	Placebo
Sahu 2019 [158]	Single center	Obstetrics and gynecology	100	24/24	100/100	NA	1g TXA bolus	No treatment
Sallam 2018 [159]	Single center	Obstetrics and gynecology	86	48/47	100/100	NA	1g TXA infusion over 100 min	Placebo
Sankar 2012 [160]	Single center	Orthopaedic surgery	50	23/24	32/36	NA	10mg/kg TXA bolus followed by 1mg/kg maintenance	Placebo
Santos 2006 [161]	Single center	Cardiac surgery	60	62/59	38/21	28/26	10mg/kg TXA bolus followed by 1mg/kg maintenance	Placebo

Seddighi 2020 [162]	Single center	Orthopaedic surgery	40	50/44	65/75	NA	10mg/kg TXA bolus followed by 0.5mg/kg maintenance	Placebo
Sentilhes 2018 [163]	Multicenter	Obstetrics and gynecology	3891	30/30	100/100	NA	1g TXA bolus	placebo
Senturk 2013 [164]	Single center	Obstetrics and gynecology	223	30/29	100/100	NA	1g TXA bolus	placebo
Seo 2012 [165]	Single center	Orthopaedic surgery	100	67/68	88/90	NA	1.5g TXA bolus	placebo
Seol 2016 [166]	Single center	Orthopaedic surgery	100	70/68	86/96	NA	10mg/kg TXA bolus followed by 10mg/kg bolus	Placebo
Seviciu 2016 [167]	Single center	Orthopaedic surgery	61	66/63	48/56	NA	20mg/kg TXA bolus	Placebo
Shaaban 2015 [168]	Single center	Obstetrics and gynecology	132	35/35	100/100	NA	10mg/kg TXA bolus followed by 1mg/kg maintenance	No treatment
Shahid 2013 [169]	Single center	Obstetrics and gynecology	74	24/25	100/100	NA	1g TXA bolus	placebo
Shakur 2010 [170]	Multicenter	Trauma	20207	35/35	16/16	NA	1g TXA bolus followed by 1g infusion over 8hr	Placebo
Shakur 2017 [171]	Multicenter	Obstetrics and gynecology	20060	NA	100/100	NA	1g TXA bolus followed by 1g bolus	Placebo
Shen 2015 [172]	Sigle center	Orthopaedic surgery	92	66/65	80/73	NA	15mg/kg TXA bolus	Placebo
Shi 2013 [173]	Multicenter	Cardiac surgery	552	60/60	23/23	NA	10mg/kg TXA bolus followed by 10mg/kg maintenance	Placebo
Shi 2013 [174]	Single center	Cardiac surgery	117	60/60	20/19	NA	15mg/kg TXA bolus	Placebo
Shi 2016 [175]	Single center	Orthopaedic surgery	96	54/56	50/52	NA	30mg/kg TXA bolus followed by 2mg/kg maintenance	Placebo
Shimizu 2011 [176]	Single center	Cardiac surgery	160	2.5/2.5	48/47	38/41	50mg/kg TXA bolus followed by 15mg/kg maintenance	Placebo

Shore-Lesserson 1996 [177]	Single center	Cardiac surgery	30	68/63	41/23	24/46	20mg/kg TXA bolus followed by 2mg/kg maintenance	Placebo
Spitler 2019 [178]	Single center	Orthopaedic surgery	94	47/43	23/30	NA	15mg/kg TXA bolus followed by 15mg/kg bolus	No treatment
Sprigg 2013 [179]	Single center	Intracranial hemorrhage	24	68/69	37/50	25/13	1g TXA bolus followed by 1g infusion over 8hr	Placebo
Sprigg 2018 [180]	Multicenter	Intracranial hemorrhage	2325	69/69	45/43	27/25	1g TXA bolus followed by 1g infusion over 8hr	Placebo
Stowers 2017 [181]	Muticenter	Orthopaedic surgery	74	71/70	47/83	NA	1.5g TXA bolus	placebo
Sun 2016 [182]	Single center	Orthopaedic surgery	180	67,67,68/68	38,62,78/82	NA	30mg/kg TXA bolus/ 15mg/kg TXA bolus followed by 15mg/kg bolus/ 10mg/kg TXA bolus followed by 10mg/kg bolus and 10mg/kg bolus	Placebo
Taghaddomi 2009 [183]	Single center	Cardiac surgery	100	55/60	24/32	88/92	1g TXA bolus followed by 400mg/hr maintenance	Placebo
Tengberg 2016 [184]	Single center	Orthopaedic surgery	75	80/75	79/64	NA	1g TXA bolus followed by 3g infusion over 24hr	Placebo
Thipparampall 2020 [185]	Single center	Orthopaedic surgery	59	48,45/55	29,11/20	NA	10mg/kg TXA bolus/ 10mg/kg TXA bolus followed by 1mg/kg maintenance	Placebo
Tian 2018 [186]	Single center	Orthopaedic surgery	100	78/80	62/72	NA	20mg/kg TXA bolus	No treatment
Topsoee 2016 [187]	Multicenter	Obstetrics and gynecology	332	48/49	NA	NA	1g TXA bolus	placebo
Tsementzis 1990 [188]	Single center	Intracranial hemorrhage	100	NA	60/48	NA	1.5g TXA bolus every 4hr	Placebo

							in the first week	
Tsutsumimoto 2011 [189]	Single center	Orthopaedic surgery	40	68/66	25/20	NA	15mg/kg TXA bolus	Placebo
Vanek 2005 [190]	Single center	Cardiac surgery	62	68/69	50/27	NA	1g TXA bolus followed by 200mg/hr maintenance	Placebo
Vara 2017 [191]	Single center	Orthopaedic surgery	102	67/66	62/55	NA	10mg/kg TXA bolus followed by 10mg/kg bolus	Placebo
Veien 2002 [192]	Single center	Orthopaedic surgery	30	71/70	73/94	NA	10mg/kg TXA bolus followed by 10mg/kg bolus	Placebo
Vel 2015 [193]	Single center	Neurosurgery	100	40/40	40/46	NA	10mg/kg TXA bolus followed by 1mg/kg/hr maintenance	Placebo
Vela 2012 [194]	Single center	Orthopaedic surgery	44	NA	NA	NA	10mg/kg TXA bolus followed by 10mg/kg bolus	Placebo
Verma 2014 [195]	Single center	Orthopaedic surgery	83	15/15	89/66	NA	10mg/kg TXA bolus followed by 1mg/kg/hr maintenance	Placebo
Vermeulen 1984 [196]	Multicenter	Intracranial hemorrhage	479	50/50	61/61	NA	6g TXA per day during the first week, 4g/day during the second week.	Placebo
Vijay 2013 [197]	Single center	Orthopaedic surgery	90	49/49	78/78	NA	10mg/kg TXA bolus	Placebo
Volquind 2014 [198]	Single center	Orthopaedic surgery	62	68/64	69/70	NA	2.5g TXA bolus	Placebo
von Holstein 1987 [199]	Single center	Gastrointestinal hemorrhage	154	62/65	31/29	NA	1g TXA bolus every 6hr For 3 days	Placebo
Wang, C 2016 [200]	Single center	Orthopaedic surgery	119	58,59/64	54,64/63	NA	10mg/kg TXA bolus/ 20mg/kg TXA bolus	Placebo

Wang, G 2012 [201]	Single center	Cardiac surgery	231	61/60	20/11	NA	1g TXA bolus followed by 400mg/hr maintenance	Placebo
Wang, J 2016 [202]	Single center	Orthopaedic surgery	198	68/70	74/72	NA	1g TXA bolus	Placebo
Wang, Q 2013 [203]	Single center	Orthopaedic surgery	60	63/62	53/69	NA	15mg/kg TXA bolus	Placebo
Wang, W 2017 [204]	Single center	Orthopaedic surgery	80	41/43	46/47	NA	10mg/kg TXA bolus followed by 1mg/kg/hr maintenance	Placebo
Watts, C 2017 [205]	Single center	Orthopaedic surgery	138	81/82	70/68	51/54	30mg/kg TXA bolus	Placebo
Wei 2006 [206]	Single center	Cardiac surgery	76	63/61	22/20	NA	0.75g TXA bolus followed by 250mg/hr maintenance	Placebo
Wong 2008 [207]	Multicenter	Orthopaedic surgery	147	57/50	29/35	NA	10mg/kg TXA bolus followed by 1mg/kg/hr maintenance	Placebo
Wu 2006 [208]	Single center	Abdominal surgery	214	57/62	26/27	NA	0.5g TXA bolus followed by 250mg bolus every 6hr	Placebo
Xie 2015 [209]	Single center	Orthopaedic surgery	90	43/43	93/96	NA	15mg/kg TXA bolus	Placebo
Xu 2012 [210]	Singe center	Orthopaedic surgery	40	19/20	40/65	NA	10mg/kg TXA bolus followed by 10mg/kg/hr maintenance	Placebo
Xu 2013 [211]	Single center	Obstetrics and gynecology	174	27/27	100/100	NA	10mg/kg TXA bolus	Placebo
Xu 2019 [212]	Single center	Orthopaedic surgery	135	63/63	53/48	NA	20mg/kg TXA bolus	Placebo
Yamasaki 2004 [213]	Single center	Orthopaedic surgery	40	56/61	5/10	NA	1g TXA bolus	No treatment
Yamasaki 2005 [214]	Single center	Orthopaedic surgery	42	NA	NA	NA	1g TXA bolus	No treatment
Yanartas 2015 [215]	Single center	Cardiac surgery	66	60/63	56/78	NA	20mg/kg TXA bolus	Placebo
Ye 2019 [216]	Single center	Orthopaedic surgery	200	65,65,66,65/ 64	NA	NA	10mg/kg TXA bolus/ 15mg/kg TXA bolus/	Placebo

							20mg/kg TXA bolus/ 30mg/kg TXA bolus	
Yen 2017 [217]	Single center	Orthopaedic surgery	65	69/70	80/87	NA	1g TXA bolus	placebo
Yi 2016 [218]	Single center	Orthopaedic surgery	100	54/57	52/42	NA	15mg/kg TXA bolus	Placebo
Yuan 2017 [219]	Single center	Orthopaedic surgery	280	64/65	56/54	NA	20mg/kg TXA bolus followed by 20mg/kg bolus	Placebo
Yutthakasemsunt 2013 [220]	Single center	Trauma	238	35/34	14/9	NA	1g TXA bolus followed by 1g infusion over 8hr	Placebo
Zabeeda 2002 [221]	Single center	Cardiac surgery	50	66/65	20/28	NA	10mg/kg TXA bolus followed by 1mg/kg/hr maintenance	Placebo
Zaman 2019 [222]	Single center	Otorhinolaryngology	176	NA	33/48	NA	10mg/kg TXA bolus	Placebo
Zekcer 2016 [223]	Single center	Orthopaedic surgery	60	NA	NA	NA	20mg/kg TXA bolus	Placebo
Zhang S 2021 [224]	Single center	Orthopaedic surgery	175	65,66/65	83,86/81	NA	20mg/kg TXA bolus/ 20mg/kg TXA bolus followed by 1g bolus at 3, 7, 12, 18, 24hr	Placebo
Zhang S 2020 [225]	Single center	Orthopaedic surgery	64	67/65	88/87	NA	20mg/kg TXA bolus followed by 1g bolus at 3, 7, 12, 18, 24hr	Placebo
Zhang S 2020 [226]	Single center	Orthopaedic surgery	122	79/76	54/44	NA	2g TXA bolus	Placebo
Zhang Y 2016 [227]	Single center	Orthopaedic surgery	50	45/43	44/54	NA	1g TXA bolus	No treatment
Zhang Y 2018 [228]	Single center	Cardiac surgery	210	59/58	21/18	NA	50mg/kg TXA bolus	Placebo
Zhao 2018 [229]	Single center	Orthopaedic surgery	80	60/60	57/50	NA	15mg/kg TXA bolus	Placebo

Zhou 2018 [230]	Single center	Orthopaedic surgery	116	63/65	79/75	NA	10mg/kg TXA bolus followed by 10mg/kg bolus	Placebo
Zhou 2019 [231]	Multicenter	Orthopaedic surgery	100	75/78	50/56	NA	1g TXA bolus	No treatment
Zhu 2020 [232]	Single center	Orthopaedic surgery	150	55,56/56	58,62/56	NA	15mg/kg TXA bolus/ 15mg/kg TXA bolus followed by 15mg/kg bolus	Placebo
Zohar 2004 [233]	Single center	Orthopaedic surgery	60	73,69/73	30,20/35	NA	15mg/kg TXA bolus followed by 10mg/kg/hr maintenance until 12hr after deflation / 15mg/kg TXA bolus followed by 10mg/kg/hr maintenance until 2hr after deflation	Placebo
Zufferey 2009 [234]	Single center	Orthopaedic surgery	110	81/82	47/49	18/21	15mg/kg TXA bolus	Placebo

TXA, tranexamic acid.

REFERENCES

1. Abdel-Aleem H, Alhusaini TK, Abdel-Aleem MA, Menoufy M, Gulmezoglu AM. Effectiveness of tranexamic acid on blood loss in patients undergoing elective cesarean section: randomized clinical trial. *J Matern Fetal Neonatal Med*. 2013;26(17):1705-9.

2. Abdul IF, Amadu MB, Adesina KT, Olarinoye AO, Omokanye LO. Adjunctive use of tranexamic acid to tourniquet in reducing haemorrhage during abdominal myomectomy - A randomized controlled trial. *Eur J Obstet Gynecol Reprod Biol*. 2019;242:150-158.

3. Aguilera X, Martinez-Zapata MJ, Bosch A, et al. Efficacy and safety of fibrin glue and tranexamic acid to prevent postoperative blood loss in total knee arthroplasty: a randomized controlled clinical trial. *J Bone Joint Surg Am*. 2013;95(22):2001-7.

4. Aguilera X, Martinez-Zapata MJ, Hinarejos P, et al. Topical and intravenous tranexamic acid reduce blood loss compared to routine hemostasis in total knee arthroplasty: a multicenter, randomized, controlled trial. *Arch Orthop Trauma Surg*. 2015; 135(7):1017-25.

5. Ahmed MR, Sayed Ahmed WA, Madny EH, Arafa AM, Said MM. Efficacy of tranexamic acid in decreasing blood loss in elective caesarean delivery. *J Matern Fetal Neonatal Med*. 2015;28(9):1014-8.

6. Ahn SW, Shim JK, Youn YN, et al. Effect of tranexamic acid on transfusion requirement in dual antiplatelet-treated anemic patients undergoing off-pump coronary artery bypass graft surgery. *Circ J*. 2012;76(1):96-101.

7. Akgul T, Buget M, Salduz A, et al. Efficacy of preoperative administration of single high dose intravenous tranexamic acid in reducing blood loss in total knee arthroplasty: A prospective clinical study. *Acta Orthop Traumatol Turc*. 2016;50(4):429-31.

8. Alimian M, Mohseni M. The effect of intravenous tranexamic acid on blood loss and surgical field quality during endoscopic sinus surgery: a placebo-controlled clinical trial. *J Clin Anesth*. 2011;23(8):611-5.

9. Alizadeh Ghavidel A, Totonchi Z, Chitsazan M, et al. Safety and efficacy of caproamin fides and tranexamic acid versus placebo in patients undergoing coronary artery revascularization. *Journal of cardiovascular and thoracic research*. 2014;6(3):197-202.

10. Altun G, Hemsinli D, Pulathan Z, Civelek A. Emergency coronary bypass surgery in patients under the influence of dualantiplatelet therapy: effects of tranexamic acid and desmopressin acetate. *Turk J Med Sci*. 2017;47(6)

11. Alvarez JC, Santiveri FX, Ramos I, Vela E, Puig L, Escolano F. Tranexamic acid reduces blood transfusion in total knee arthroplasty even when a blood conservation program is applied. *Transfusion*. 2008;48(3):519-25.
12. Alvarez J, Santiveri FJ, Ramos MI, Gallart L, Aguilera L, Puig-Verdie L. Clinical trial on the effect of tranexamic acid on bleeding and fibrinolysis in primary hip and knee replacement. *Rev Esp Anestesiol Reanim*. 2019;66(6):299-306.
13. Andreasen JJ, Nielsen C. Prophylactic tranexamic acid in elective, primary coronary artery bypass surgery using cardiopulmonary bypass. *Eur J Cardiothorac Surg*. 2004;26(2):311-7.
14. Apipan B, Rummasak D, Narainthonsaenee T. The effect of different dosage regimens of tranexamic acid on blood loss in bimaxillary osteotomy: a randomized, double-blind, placebo-controlled study. *Int J Oral Maxillofac Surg*. 2018;47(5):608-612.
15. Armellin G, Casella S, Guzzinati S, Pasini L, Marcassa A, Giron G. Tranexamic acid in aortic valve replacement. *J Cardiothorac Vasc Anesth*. Jun 2011;(3):331-5.
16. Auvinen O, Baer GA, Nordback I, Saaristo J. Antifibrinolytic therapy for prevention of hemorrhage during surgery of the thyroid gland. *Klin Wochenschr*. 1987;65(6):253-5.
17. Avvisati G, Buller HR, Wouter Ten Cate J, elli F. Tranexamic acid for control of haemorrhage in acute promyelocytic leukaemia. *Lancet*. 1989;2(8655):122-124.
18. Barrachina B, Lopez-Picado A, Remon M, et al. Tranexamic Acid Compared with Placebo for Reducing Total Blood Loss in Hip Replacement Surgery: a Randomized Clinical Trial. *Anesthesia and analgesia*. 2016;122(4):986-995.
19. Baruah RK, Borah PJ, Haque R. Use of tranexamic acid in dynamic hip screw plate fixation for trochanteric fractures. *J Orthop Surg (Hong Kong)*. 2016;24(3):379-382.
20. Batibay SG, Turkmen I, Duman S, Camur S, Saglam N, Batibay S. Is tranexamic acid safe and reliable during tibial intramedullary nailing? *Ulus Travma Acil Cerrahi Derg*. 2018;24(6):575-580.
21. Benoni G, Fredin H. Fibrinolytic inhibition with tranexamic acid reduces blood loss and blood transfusion after knee arthroplasty: a prospective, randomised, double-blind study of 86 patients. *J Bone Joint Surg Br*. 1996;78(3):434-40.
22. Benoni G, Lethagen S, Nilsson P, Fredin H. Tranexamic acid, given at the end of the operation, does not reduce postoperative blood loss in hip arthroplasty. *Acta Orthop Scand*. 2000;71(3):250-4.
23. Benoni G, Fredin H, Knebel R, Nilsson P. Blood conservation with tranexamic acid in total hip arthroplasty: a randomized, double-blind study in 40 primary operations. *Acta Orthop Scand*. 2001;72(5):442-8.

24. Bidolegui F, Arce G, Lugones A, Pereira S, Vindver G. Tranexamic Acid Reduces Blood Loss and Transfusion in Patients Undergoing Total Knee Arthroplasty without Tourniquet: A Prospective Randomized Controlled Trial. *Open Orthop J*. 2014;8:250-4.
25. Boylan JF, Klinck JR, ler AN, et al. Tranexamic acid reduces blood loss, transfusion requirements, and coagulation factor use in primary orthotopic liver transplantation. *Anesthesiology*. 1996;85(5):1043-8; discussion 30A-31A.
26. Brown RS, Thwaites BK, Mongan PD. Tranexamic acid is effective in decreasing postoperative bleeding and transfusions in primary coronary artery bypass operations: a double-blind, randomized, placebo-controlled trial. *Anesth Analg*. 1997;85(5):963-70.
27. Bulutcu FS, Ozbek U, Polat B, Yalcin Y, Karaci AR, Bayindir O. Which may be effective to reduce blood loss after cardiac operations in cyanotic children: tranexamic acid, aprotinin or a combination? *Paediatr Anaesth*. 2005;15(1):41-6.
28. Caglar GS, Tasci Y, Kayikcioglu F, Haberal A. Intravenous tranexamic acid use in myomectomy: a prospective randomized double-blind placebo controlled study. *Eur J Obstet Gynecol Reprod Biol*. 2008;137(2):227-31.
29. Cansancao AL, Conde-Green A, David JA, Cansancao B, Vidigal RA. Use of Tranexamic Acid to Reduce Blood Loss in Liposuction. *Plast Reconstr Surg*. 2018;141(5):1132-1135.
30. Carabini LM, Morel, NC, et al. A Randomized Controlled Trial of Low-Dose Tranexamic Acid versus Placebo to Reduce Red Blood Cell Transfusion During Complex Multilevel Spine Fusion Surgery. *World Neurosurg*. 2018;110:e572-e579.
31. Casati V, Bellotti F, Gerli C, et al. Tranexamic acid administration after cardiac surgery: a prospective, randomized, double-blind, placebo-controlled study. *Anesthesiology*. 2001; 94(1):8-14.
32. Casati V, relli L, Speziali G, Calori G, Grasso MA, Spagnolo S. Hemostatic effects of tranexamic acid in elective thoracic aortic surgery: a prospective, randomized, double-blind, placebo-controlled study. *J Thorac Cardiovasc Surg*. 2002;123(6):1084-91.
33. Casati V, Della Valle P, Benussi S, et al. Effects of tranexamic acid on postoperative bleeding and related hematochemical variables in coronary surgery: Comparison between on-pump and off-pump techniques. *J Thorac Cardiovasc Surg*. 2004;128(1):83-91.
34. Chakravarthy M, Muniraj G, Patil S, Suryaprakash S, Mitra S, Shivalingappa B. A randomized prospective analysis of alteration of hemostatic function in patients receiving tranexamic acid and hydroxyethyl starch (130/0.4) undergoing off pump coronary artery bypass surgery. *Ann Card Anaesth*. 2012;15(2):105-10.

35. Chakroun-Walha O, Samet A, Jerbi M, et al. Benefits of the tranexamic acid in head trauma with no extracranial bleeding: a prospective follow-up of 180 patients. *Eur J Trauma Emerg Surg*. 2019;45(4):719-726.
36. Chandra B. Treatment of subarachnoid hemorrhage from ruptured intracranial aneurysm with tranexamic acid: a double-blind clinical trial. *Ann Neurol*. 1978;3(6):502-4.
37. Chareancholvanich K, Siriwattanasakul P, Narkbunnam R, Pornrattanamaneewong C. Temporary clamping of drain combined with tranexamic acid reduce blood loss after total knee arthroplasty: a prospective randomized controlled trial. *BMC Musculoskelet Disord*. 2012;13:124.
38. Charoencholvanich K, Siriwattanasakul P. Tranexamic acid reduces blood loss and blood transfusion after TKA: a prospective randomized controlled trial. *Clin Orthop Relat Res*. 2011;469(10):2874-80.
39. Chauhan S, Bisoi A, Modi R, Gharde P, Rajesh MR. Tranexamic acid in paediatric cardiac surgery. *Indian J Med Res*. 2003;118:86-9.
40. Chauhan S, Das SN, Bisoi A, Kale S, Kiran U. Comparison of epsilon aminocaproic acid and tranexamic acid in pediatric cardiac surgery. *J Cardiothorac Vasc Anesth*. 2004;18(2):141-3.
41. Chen X, Cao X, Yang C, Guo K, Zhu Q, Zhu J. Effectiveness and Safety of Fixed-Dose Tranexamic Acid in Simultaneous Bilateral Total Knee Arthroplasty: A Randomized Double-Blind Controlled Trial. *J Arthroplasty*. 2016;31(11):2471-2475.
42. Chen F, Jiang Z, Li M, Zhu X. Efficacy and safety of perioperative tranexamic acid in elderly patients undergoing trochanteric fracture surgery: a randomised controlled trial. *Hong Kong Med J*. 2019;25(2):120-126.
43. Chin J, Blackett J, Kieser DC, Frampton C, Hooper G. The Value of Routine Intravenous Tranexamic Acid in Total Hip Arthroplasty: A Preliminary Study. Adv Orthop. 2020;2943827.
44. Choi WS, Irwin MG, Samman N. The effect of tranexamic acid on blood loss during orthognathic surgery: a randomized controlled trial. *J Oral Maxillofac Surg*. 2009;67(1):125-33.
45. Christabel A, Muthusekhar MR, Narayanan V, et al. Effectiveness of tranexamic acid on intraoperative blood loss in isolated le Fort i osteotomies - A prospective, triple blinded randomized clinical trial. *Journal of Cranio-Maxillofacial Surgery*. 2014;42(7):1221-1224.
46. Claeys MA, Vermeersch N, Haentjens P. Reduction of blood loss with tranexamic acid in primary total hip replacement surgery. *Acta Chir Belg*. 2007;107(4):397-401.

47. Clave A, Gerard R, Lacroix J, et al. A randomized, double-blind, placebo-controlled trial on the efficacy of tranexamic acid combined with rivaroxaban thromboprophylaxis in reducing blood loss after primary cementless total hip arthroplasty. *Bone Joint J*. 2019;101(2):207-212.
48. Cohen JC, Glasgold RA, Alloju LM, Glasgold MJ. Effects of Intravenous Tranexamic Acid During Rhytidectomy: A Randomized, Controlled, Double-Blind Pilot Study. *Aesthet Surg J*. 2021;41(2):155-160.
49. Colomina MJ, Koo M, Basora M, Pizones J, Mora L, Bago J. Intraoperative tranexamic acid use in major spine surgery in adults: a multicentre, randomized, placebo-controlled trialdagger. *Br J Anaesth*. 2016;118(3):380-390.
50. Crescenti A, Borghi G, Bignami E, et al. Intraoperative use of tranexamic acid to reduce transfusion rate in patients undergoing radical retropubic prostatectomy: double blind, randomised, placebo controlled trial. *Bmj*. 2011;343:d5701.
51. Cvetanovich GL, Fillingham YA, O'Brien M, et al. Tranexamic acid reduces blood loss after primary shoulder arthroplasty: a double-blind, placebo-controlled, prospective, randomized controlled trial. *JSES Open Access*. 2018;2(1):23-27.
52. Dadure C, Sauter M, Bringuier S, et al. Intraoperative tranexamic acid reduces blood transfusion in children undergoing craniosynostosis surgery: a randomized double-blind study. *Anesthesiology*. 2011;114(4):856-61.
53. Diprose P, Herbertson MJ, O'Shaughnessy D, Deakin CD, Gill RS. Reducing allogeneic transfusion in cardiac surgery: a randomized double-blind placebo-controlled trial of antifibrinolytic therapies used in addition to intra-operative cell salvage. *Br J Anaesth*. 2005;94(3):271-8.
54. Ducloy-Bouthors AS, Jude B, Duhamel A, et al. High-dose tranexamic acid reduces blood loss in postpartum haemorrhage. *Crit Care*. 2011 2011;15(2):R117.
55. Ebrahimi P, Mozafari J, Ilkhchi RB, Hanafi MG, Mousavinejad M. Intravenous Tranexamic Acid for Subdural and Epidural Intracranial Hemorrhage: Randomized, Double-Blind, Placebo-Controlled Trial. *Rev Recent Clin Trials*. 2019;14(4):286-291.
56. Ekback G, Axelsson K, Ryttberg L, et al. Tranexamic acid reduces blood loss in total hip replacement surgery. *Anesth Analg*. 2000;91(5):1124-30.
57. Eldaba AA, Amr YM, Albirmawy OA. Effects of tranexamic acid during endoscopic sinsus surgery in children. *Saudi J Anaesth*. 2013;7(3):229-33.
58. Elwatidy S, Jamjoom Z, Elgamal E, Zakaria A, Turkistani A, El-Dawlatly A. Efficacy and safety of prophylactic large dose of tranexamic acid in spine surgery: a prospective, randomized, double-blind, placebo-controlled study. *Spine (Phila Pa 1976)*. 2008;33(24):2577-80.

59. Emara WM, Moez KK, Elkhouly AH. Topical versus intravenous tranexamic acid as a blood conservation intervention for reduction of post-operative bleeding in hemiarthroplasty. *Anesth Essays Res*. 2014;8(1):48-53.
60. Engel JM, Hohaus T, Ruwoldt R, Menges T, Jurgensen I, Hempelmann G. Regional hemostatic status and blood requirements after total knee arthroplasty with and without tranexamic acid or aprotinin. *Anesth Analg*. 2001;92(3):775-80.
61. Engqvist A, Brostrom O, von Feilitzen F, et al. Tranexamic acid in massive haemorrhage from the upper gastrointestinal tract: a double-blind study. *Scand J Gastroenterol*. 1979;14(7):839-44.
62. Esfandiari BR, Bistgani MM, Kabiri M. Low dose tranexamic acid effect on post-coronary artery bypass grafting bleeding. *Asian Cardiovasc Thorac Ann*. 2012;21(6):669-74.
63. Ezhevskaya AA, Prusakova ZB, Zagrekov VI, Sosnin AV, Milenovic M. Efficacy assessment of epidural blockade and tranexamic acid application in idiopathic scoliosis surgery. *Sovremennye Tehnologii v Medicine*. 2018;10(4):164-172.
64. Faraoni D, Cacheux C, Van Aelbrouck C, Ickx BE, Barvais L, Levy JH. Effect of two doses of tranexamic acid on fibrinolysis evaluated by thromboelastography during cardiac surgery: a randomised, controlled study. *Eur J Anaesthesiol*. 2014;31(9):491-8.
65. Farrokhi MR, Kazemi AP, Eftekharian HR, Akbari K. Efficacy of prophylactic low dose of tranexamic acid in spinal fixation surgery: a randomized clinical trial. *J Neurosurg Anesthesiol*. 2011;23(4):290-6.
66. Felli L, Revello S, Burastero G, et al. Single Intravenous Administration of Tranexamic Acid in Anterior Cruciate Ligament Reconstruction to Reduce Postoperative Hemarthrosis and Increase Functional Outcomes in the Early Phase of Postoperative Rehabilitation: A Randomized Controlled Trial. *Arthroscopy*. 2019;35(1):149-157.
67. Fenger-Eriksen C, D'Amore Lindholm A, Norholt SE, et al. Reduced perioperative blood loss in children undergoing craniosynostosis surgery using prolonged tranexamic acid infusion: a randomised trial. *Br J Anaesth*. 2019;122(6):760-766.
68. Fodstad H, Forssell A, Liliequist B, Schannong M. Antifibrinolysis with tranexamic acid in aneurysmal subarachnoid hemorrhage: a consecutive controlled clinical trial. *Neurosurgery*. 1981;8(2):158-65.
69. Fraval A, Effeney P, Fiddelaers L, Smith B, Towell B, Tran P. OBTAIN A: Outcome Benefits of Tranexamic Acid in Hip Arthroplasty. A Randomized Double-Blinded Controlled Trial. *J Arthroplasty*. 2017;32(5):1516-1519.

70. Gai MY, Wu LF, Su QF, Tatsumoto K. Clinical observation of blood loss reduced by tranexamic acid during and after caesarian section: a multi-center, randomized trial. *Eur J Obstet Gynecol Reprod Biol*. 2004;112(2):154-7.
71. Garg B, Dhatt S, Chakraborty S. Use of single-dose tranexamic acid to reduce blood loss in operative thoracolumbar trauma: A comparative study. *Spine Journal*. 2012;12(9):93S.
72. Garneti N, Field J. Bone bleeding during total hip arthroplasty after administration of tranexamic acid. *J Arthroplasty*. 2004;19(4):488-92.
73. Goobie SM, Meier PM, Pereira LM, et al. Efficacy of tranexamic acid in pediatric craniosynostosis surgery: a double-blind, placebo-controlled trial. *Anesthesiology*. 2011;114(4):862-71.
74. Goobie SM, Zurakowski D, Glotzbecker MP, et al. Tranexamic acid is efficacious at decreasing the rate of blood loss in adolescent scoliosis surgery: a randomized placebo-controlled trial. *Journal of bone and joint surgery - american volume*. 2018;100(23):2024-2032.
75. Good L, Peterson E, Lis, er B. Tranexamic acid decreases external blood loss but not hidden blood loss in total knee replacement. *Br J Anaesth*. 2003;90(5):596-9.
76. Goswami U, Sarangi S, Gupta S, Babbar S. Comparative evaluation of two doses of tranexamic acid used prophylactically in anemic parturients for lower segment cesarean section: A double-blind randomized case control prospective trial. *Saudi J Anaesth*. 2013;7(4):427-31.
77. Greiff G, Stenseth R, Wahba A, et al. Tranexamic acid reduces blood transfusions in elderly patients undergoing combined aortic valve and coronary artery bypass graft surgery: a randomized controlled trial. *J Cardiothorac Vasc Anesth*. 2012;26(2):232-8.
78. Gungorduk K, Yildirim G, Asicioglu O, Gungorduk OC, Sudolmus S, Ark C. Efficacy of intravenous tranexamic acid in reducing blood loss after elective cesarean section: a prospective, randomized, double-blind, placebo-controlled study. *Am J Perinatol*. 2011;28(3):233-40.
79. Gungorduk K, Asicioğlu O, Yildirim G, Ark C, Tekirdağ A, Besimoglu B. Can intravenous injection of tranexamic acid be used in routine practice with active management of the third stage of labor in vaginal delivery? A randomized controlled study. *American Journal of Perinatology*. 2013;30(5):407-413.
80. Gupta K, Rastogi B, Krishan A, Gupta A, Singh VP, Agarwal S. The prophylactic role of tranexamic acid to reduce blood loss during radical surgery: A prospective study. *Anesth Essays Res*. 2012;6(1):70-3.

81. Hardy JF, Bélisle S, Dupont C, et al. Prophylactic tranexamic acid and ε-aminocaproic acid for primary myocardial revascularization. *Annals of Thoracic Surgery*. 1998;65(2):371-376.
82. Helito CP, Bonadio MB, Sobrado MF, et al. Comparison of Floseal® and Tranexamic Acid for Bleeding Control after Total Knee Arthroplasty: a Prospective Randomized Study. *Clinics (Sao Paulo)*. 2019;74:e1186.
83. Hiippala S, Strid L, Wennerstr, et al. Tranexamic acid (Cyklokapron) reduces perioperative blood loss associated with total knee arthroplasty. *Br J Anaesth*. 1995;74(5):534-7.
84. Hiippala ST, Strid LJ, Wennerstr, et al. Tranexamic acid radically decreases blood loss and transfusions associated with total knee arthroplasty. *Anesthesia and Analgesia*. 1997;84(4):839-844.
85. Hillman J, Fridriksson S, Nilsson O, et al. Immediate administration of tranexamic acid and reduced incidence of early rebleeding after aneurysmal subarachnoid hemorrhage: a prospective randomized study. *J Neurosurg*. 2002;97(4):771-8.
86. Hooda B, Chouhan RS, Rath GP. Effect of tranexamic acid on intra-operative blood loss and transfusion requirements in patients undergoing excision of intracranial meningioma. *Journal of Neurosurgical Anesthesiology*. 2015;27(4):384-385.
87. Horrow JC, Van Riper DF, Strong MD, Grunewald KE, Parmet JL. The dose-response relationship of tranexamic acid. *Anesthesiology*. 1991;82(2):383-92.
88. Horrow JC, Van Riper DF, Strong MD, Brodsky I, Parmet JL. Hemostatic effects of tranexamic acid and desmopressin during cardiac surgery. *Circulation*. 1995;84(5):2063-70.
89. Hsu CH, Lin PC, Kuo FC, Wang JW. A regime of two intravenous injections of tranexamic acid reduces blood loss in minimally invasive total hip arthroplasty: a prospective randomised double-blind study. *Bone Joint J*. 2015;97(7):905-10.
90. Husted H, Blond L, Sonne-Holm S, Holm G, Jacobsen TW, Gebuhr P. Tranexamic acid reduces blood loss and blood transfusions in primary total hip arthroplasty: a prospective randomized double-blind study in 40 patients. *Acta Orthop Scand*. 2003;74(6):665-9.
91. Jansen AJ, Andreica S, Claeys M, D'Haese J, Camu F, Jochmans K. Use of tranexamic acid for an effective blood conservation strategy after total knee arthroplasty. *Br J Anaesth*. 1999;83(4):596-601.
92. Jares M, Vanek T, Straka Z, Brucek P. Tranexamic acid reduces bleeding after off-pump coronary artery bypass grafting. *J Cardiovasc Surg (Torino)*. 2003;44(2):205-8.

93. Jaszczyk M, Kozerawski D, Kolodziej L, Kazimierczak A, Sarnecki P, Sieczka L. Effect of Single Preoperative Dose of Tranexamic Acid on Blood Loss and Transfusion in Hip Arthroplasty. *Ortop Traumatol Rehabil*. 2015;17(3):265-73.
94. Johansson T, Pettersson LG, Lis, er B. Tranexamic acid in total hip arthroplasty saves blood and money: a randomized, double-blind study in 100 patients. *Acta Orthop*. 2005;76(3):314-9.
95. Jokar A, Ahmadi K, Salehi T, Sharif-Alhoseini M, Rahimi-Movaghar V. The effect of tranexamic acid in traumatic brain injury: A randomized controlled trial. *Chin J Traumatol*. 2017;20(1):49-51.
96. Kakar PN, Gupta N, Govil P, Shah V. Efficacy and Safety of Tranexamic Acid in Control of Bleeding Following TKR: A Randomized Clinical Trial. *Indian J Anaesth*. 2009;53(6):667-71.
97. Karaaslan F, Karaoglu S, Yurdakul E. Reducing Intra-articular Hemarthrosis After Arthroscopic Anterior Cruciate Ligament Reconstruction by the Administration of Intravenous Tranexamic Acid: A Prospective, Randomized Controlled Trial. *Am J Sports Med*. 2015;43(11):2720-6.
98. Karski JM, Teasdale SJ, Norman P, et al. Prevention of bleeding after cardiopulmonary bypass with high-dose tranexamic acid: Double-blind, randomized clinical trial. *Journal of Thoracic and Cardiovascular Surgery*. 1995;110(3):835-842.
99. Karski J, Djaiani G, Carroll J, et al. Tranexamic acid and early saphenous vein graft patency in conventional coronary artery bypass graft surgery: a prospective randomized controlled clinical trial. *J Thorac Cardiovasc Surg*. 2005;130(2):309-14.
100. Kaste M, Ramsay M. Tranexamic acid in subarachnoid hemorrhage. A double-blind study. *Stroke*. 1979;10(5):519-22.
101. Katsaros D, Petricevic M, Snow NJ, Woodhall DD, Van Bergen R. Tranexamic acid reduces postbypass blood use: a double-blinded, prospective, randomized study of 210 patients. *Ann Thorac Surg*. 1996;61(4):1131-5.
102. Kazemi SM, Mosaffa F, Eajazi A, et al. The effect of tranexamic acid on reducing blood loss in cementless total hip arthroplasty under epidural anesthesia. *Orthopedics*. 2010;33(1):17.
103. Keyhani S, Esmailiejah AA, Abbasian MR, Safdari F. Which Route of Tranexamic Acid Administration is More Effective to Reduce Blood Loss Following Total Knee Arthroplasty? *Arch Bone Jt Surg*. 2016;4(1):65-9.
104. Kim TK, Chang CB, Kang YG, et al. Clinical value of tranexamic acid in unilateral and simultaneous bilateral TKAs under a contemporary blood-saving

protocol: a randomized controlled trial. *Knee Surg Sports Traumatol Arthrosc*. 2014;22(8):1870-8.
105. Kim KT, Kim CK, Kim YC, et al. The effectiveness of low-dose and high-dose tranexamic acid in posterior lumbar interbody fusion: a double-blinded, placebo-controlled randomized study. *Eur Spine J*. 2017;26(11):2851-2857.
106. Kimura OS, Freitas EH, Duarte ME, Cavalcanti AS, Fern, es MB. Tranexamic acid use in high-risk blood transfusion patients undergoing total hip replacement: a randomised controlled trial. *Hip Int*. 2019;1120700019889947.
107. Kuitunen A, Hiippala S, Vahtera E, Rasi V, Salmenpera M. The effects of aprotinin and tranexamic acid on thrombin generation and fibrinolytic response after cardiac surgery. *Acta Anaesthesiol Scand*. 2005;49(9):1272-9.
108. Kulkarni AP, Chaukar DA, Patil VP, Metgudmath RB, Hawaldar RW, Divatia JV. Does tranexamic acid reduce blood loss during head and neck cancer surgery? *Indian J Anaesth*. 2016;60(1):19-24.
109. Kundu R, Das A, Basunia SR, Bhattacharyya T, Chattopadhyay S, Mukherjee A. Does a single loading dose of tranexamic acid reduce perioperative blood loss and transfusion requirements after total knee replacement surgery? A randomized, controlled trial. *J Nat Sci Biol Med*. 2015;6(1):94-9.
110. Lack WD, Crist BD, Seymour RB, Harvin W, Karunakar MA. Effect of Tranexamic Acid on Transfusion: A Randomized Clinical Trial in Acetabular Fracture Surgery. *J Orthop Trauma*. 2017;31(10):526-530.
111. Later AFL, Maas JJ, Engbers FHM, et al. Tranexamic acid and aprotinin in low- and intermediate-risk cardiac surgery: a non-sponsored, double-blind, randomised, placebo-controlled trial. *European Journal of Cardio-thoracic Surgery*. 2009;36(2):322-329.
112. Lee SH, Cho KY, Khurana S, Kim KI. Less blood loss under concomitant administration of tranexamic acid and indirect factor Xa inhibitor following total knee arthroplasty: a prospective randomized controlled trial. *Knee Surg Sports Traumatol Arthrosc*. 2013;21(11):2611-7.
113. Lee YC, Park SJ, Kim JS, Cho CH. Effect of tranexamic acid on reducing postoperative blood loss in combined hypotensive epidural anesthesia and general anesthesia for total hip replacement. *J Clin Anesth*. 2013;25(5):393-398.
114. Lei Y, Xie J, Huang Q, Huang W, Pei F. Additional benefits of multiple-dose tranexamic acid to anti-fibrinolysis and anti-inflammation in total knee arthroplasty: a randomized controlled trial. *Arch Orthop Trauma Surg*. 2020;140(8):1087-1095.

115. Lemay E, Guay J, Cote C, Roy A. Tranexamic acid reduces the need for allogenic red blood cell transfusions in patients undergoing total hip replacement. *Can J Anaesth*. 2004;51(1):31-7.
116. Lin PC, Hsu CH, Huang CC, Chen WS, Wang JW. The blood-saving effect of tranexamic acid in minimally invasive total knee replacement: is an additional pre-operative injection effective? *J Bone Joint Surg Br*. 2012;94(7):932-6.
117. Liu W, Yang C, Huang X, Liu R. Tranexamic Acid Reduces Occult Blood Loss, Blood Transfusion, and Improves Recovery of Knee Function after Total Knee Arthroplasty: a Comparative Study. *Journal of knee surgery*. 2018;31(3):239-246
118. Liu YF, Hong CK, Hsu KL, et al. Intravenous Administration of Tranexamic Acid Significantly Improved Clarity of the Visual Field in Arthroscopic Shoulder Surgery. A Prospective, Double-Blind, and Randomized Controlled Trial. *Arthroscopy*. 2020;36(3):640-647
119. Lundin ES, Johansson T, Zachrisson H, et al. Single-dose tranexamic acid in advanced ovarian cancer surgery reduces blood loss and transfusions: double-blind placebo-controlled randomized multicenter study. *Acta Obstet Gynecol Scand*. 2014;93(4):335-44.
120. Luo X, He S, Lin Z, Li Z, Huang C, Li Q. Efficacy and Safety of Tranexamic Acid for Controlling Bleeding During Surgical Treatment of Intertrochanteric Fragility Fracture with Proximal Femoral Nail Anti-rotation: A Randomized Controlled Trial. *Indian J Orthop*. 2019;53(2):263-269.
121. Ma K, Cao C, Wang Q, Luan F, Li Q. The reduction in blood loss with an intravenous drip of tranexamic acid in decompression and fusion surgery for degenerative lumbar spinal stenosis: A randomized controlled trial. *International Journal of Clinical and Experimental Medicine*. 2019;12(5):6116-6121.
122. MacGillivray RG, Tarabichi SB, Hawari MF, Raoof NT. Tranexamic acid to reduce blood loss after bilateral total knee arthroplasty: a prospective, randomized double blind study. *J Arthroplasty*. 2011;26(1):24-8.
123. Maddali MM, Rajakumar MC. Tranexamic acid and primary coronary artery bypass surgery: a prospective study. *Asian Cardiovasc Thorac Ann*. 2007;15(4):313-9.
124. Maged AM, Helal OM, Elsherbini MM, et al. A randomized placebo-controlled trial of preoperative tranexamic acid among women undergoing elective cesarean delivery. *Int J Gynaecol Obstet*. 2015;131(3):265-8.
125. Mahmood A, Sawalha S, Borbora A, Kumar G, Peter V. Tranexamic acid versus fibrin sealant in primary total hip replacement: a comparative study. *Eur J Orthop Surg Traumatol*. 2017;27(7):923-928.

126. Maniar RN, Kumar G, Singhi T, Nayak RM, Maniar PR. Most effective regimen of tranexamic acid in knee arthroplasty: a prospective randomized controlled study in 240 patients. *Clin Orthop Relat Res*. 2012;470(9):2605-12.
127. Mansouri M, Attary M, Bagheri K, Massoumi G, Ghavami B. Comparative evaluation of the effects of tranexamic acid and low-dose aprotinin on post-valvular heart surgery bleeding and allogenic transfusion. *Interact Cardiovasc Thorac Surg*. 2012;15(1):23-7.
128. Mehr-Aein A, Sadeghi M, Madani-civi M. Does tranexamic acid reduce blood loss in off-pump coronary artery bypass? *Asian Cardiovasc Thorac Ann*. 2007;15(4):285-9.
129. Meng QQ, Pan N, Xiong JY, Liu N. Tranexamic acid is beneficial for reducing perioperative blood loss in transurethral resection of the prostate. *Exp Ther Med*. 2019;17(1):943-947.
130. Misfeld M, Dubbert S, Eleftheriadis S, Siemens HJ, Wagner T, Sievers HH. Fibrinolysis-adjusted perioperative low-dose aprotinin reduces blood loss in bypass operations. *Ann Thorac Surg*. 1998;66(3):792-9.
131. Molloy DO, Archbold HA, Ogonda L, et al. Comparison of topical fibrin spray and tranexamic acid on blood loss after total knee replacement: a prospective, randomised controlled trial. *J Bone Joint Surg Br*. 2007;89(3):306-9.
132. Monaco F, Nardelli P, Pasin L, et al. Tranexamic acid in open aortic aneurysm surgery: a randomised clinical trial. *Br J Anaesth*. 2020;124(1):35-43.
133. Motififard M, Tahririan MA, Saneie M, Badiei S, Nemati A. Low Dose Perioperative Intravenous Tranexamic Acid in Patients Undergoing Total Knee Arthroplasty: A Double-Blind Randomized Placebo Controlled Clinical Trial. *J Blood Transfus*. 2015;2015:948304.
134. Mu X, Wei J, Wang C, et al. Intravenous Administration of Tranexamic Acid Significantly Reduces Visible and Hidden Blood Loss Compared with Its Topical Administration for Double-Segment Posterior Lumbar Interbody Fusion: a Single-Center, Placebo-Controlled, Randomized Trial. *World neurosurgery*. 2019;122:e821-e827
135. Myles PS, Smith JA, Forbes A, et al. Tranexamic acid in patients undergoing coronary-artery surgery. *New England Journal of Medicine*. 2017;376(2):136-148.
136. Na HS, Shin HJ, Lee YJ, Kim JH, Koo KH, Do SH. The effect of tranexamic acid on blood coagulation in total hip replacement arthroplasty: rotational thromboelastographic (ROTEM(R)) analysis. *Anaesthesia*. 2016;71(1):67-75.
137. Nagabhushan RM, Shetty AP, Dumpa SR, Subramanian B, Kanna RM, Shanmuganathan R. Effectiveness and Safety of Batroxobin, Tranexamic Acid and a

Combination in Reduction of Blood Loss in Lumbar Spinal Fusion Surgery. *Spine (Phila Pa 1976)*. 2018;43(5):E267-E273.
138. Neilipovitz DT, Murto K, Hall L, Barrowman NJ, Splinter WM. A randomized trial of tranexamic acid to reduce blood transfusion for scoliosis surgery. *Anesth Analg*. 2001;93(1):82-7.
139. Nejad MHG, Baharestani B, Esf, iari R, Hashemi J, Panahipoor A. Evaluation and comparison of using low-dose aprotinin and tranexamic acid in CABG: a double blind randomized clinical trial. *Journal of tehran university heart center*. 2012;7(1):15-18.
140. Niskanen RO, Korkala OL. Tranexamic acid reduces blood loss in cemented hip arthroplasty: a randomized, double-blind study of 39 patients with osteoarthritis. *Acta Orthop*. 2005;76(6):829-32.
141. Nugent M, May JH, Parker JD, et al. Does Tranexamic Acid Reduce Knee Swelling and Improve Early Function Following Arthroscopic Meniscectomy? A Double-Blind Randomized Controlled Trial. *Orthop J Sports Med*. 2019;7(8):2325967119866122.
142. Oremus K, Sostaric S, Trkulja V, Haspl M. Influence of tranexamic acid on postoperative autologous blood retransfusion in primary total hip and knee arthroplasty: a randomized controlled trial. *Transfusion*. 2014;54(1):31-41.
143. Orpen NM, Little C, Walker G, Crawfurd EJ. Tranexamic acid reduces early post-operative blood loss after total knee arthroplasty: a prospective randomised controlled trial of 29 patients. *Knee*. 2006;13(2):106-10.
144. Painter TW, Daly DJ, Kluger R, et al. Intravenous tranexamic acid and lower limb arthroplasty-a randomised controlled feasibility study. *Anaesth Intensive Care*. 2018;46(4):386-395.
145. Palija S, Bijeljac S, Manojlovic S, et al. Effectiveness of different doses and routes of administration of tranexamic acid for total hip replacement. *Int Orthop*. 2020: https://doi.org/10.1007/s00264-020-04585-y
146. Peters A, Verma K, Slobodyanyuk K, et al. Antifibrinolytics reduce blood loss in adult spinal deformity surgery: a prospective, randomized controlled trial. *Spine (Phila Pa 1976)*. 2015;40(8):E443-9.
147. Pleym H, Stenseth R, Wahba A, Bjella L, Karevold A, Dale O. Single-dose tranexamic acid reduces postoperative bleeding after coronary surgery in patients treated with aspirin until surgery. *Anesth Analg*. 2003;96(4):923-8, table of contents.
148. Prakash J, Seon JK, Park YJ, Jin C, Song EK. A randomized control trial to evaluate the effectiveness of intravenous, intra-articular and topical wash regimes of

tranexamic acid in primary total knee arthroplasty. *J Orthop Surg (Hong Kong)*. 2017;25(1):2309499017693529.
149. Prasad R, Patki A, Padhy S, Ramch, ran G. Single intravenous bolus versus perioperative continuous infusion of tranexamic acid to reduce blood loss in abdominal oncosurgical procedures: A prospective randomized double-blind clinical study. *J Anaesthesiol Clin Pharmacol*. 2018;34(4):529-534.
150. Raksakietisak M, Sathitkarnmanee B, Srisaen P, et al. Two Doses of Tranexamic Acid Reduce Blood Transfusion in Complex Spine Surgery: A Prospective Randomized Study. *Spine (Phila Pa 1976)*. 2015;40(24):E1257-63.
151. Ramezani AR, Ahmadieh H, Ghaseminejad AK, Yazdani S, Golestan B. Effect of tranexamic acid on early postvitrectomy diabetic haemorrhage; a randomised clinical trial. *Br J Ophthalmol*. 2005;89(8):1041-4.
152. Ray I, Bhattacharya R, Chakraborty S, Bagchi C, Mukhopadhyay S. Role of Intravenous Tranexamic Acid on Caesarean Blood Loss: A Prospective Randomised Study. *J Obstet Gynaecol India*. 2016;66:347-52.
153. Reid RW, Zimmerman AA, Laussen PC, Mayer JE, Gorlin JB, Burrows FA. The efficacy of tranexamic acid versus placebo in decreasing blood loss in pediatric patients undergoing repeat cardiac surgery. *Anesth Analg*. 1997;84(5):990-6.
154. CRASH-3 trial collaborators. Effects of tranexamic acid on death, disability, vascular occlusive events and other morbidities in patients with acute traumatic brain injury (CRASH-3): a randomised, placebo-controlled trial. *Lancet*. 2019;394(10210):1713-1723.
155. HALT-IT Trial Collaborators. Effects of a high-dose 24-h infusion of tranexamic acid on death and thromboembolic events in patients with acute gastrointestinal bleeding (HALT-IT): an international randomised, double-blind, placebo-controlled trial. *Lancet*. 2020;395(10241):1927-1936.
156. Roos Y. Antifibrinolytic treatment in subarachnoid hemorrhage: a randomized placebo-controlled trial. *Neurology*. 2000;54(1):77-82.
157. Rowell SE, Meier EN, McKnight B, Kannas D, et al. Effect of Out-of-Hospital Tranexamic Acid vs Placebo on 6-Month Functional Neurologic Outcomes in Patients With Moderate or Severe Traumatic Brain Injury. *JAMA*. 2020;324(10):961-974.¥
158. Sahu J, Mishra N. Role of intravenous tranexamic acid in reducing blood loss during caesarean section: Study at tribal-dominated area hospital in Chhattisgarh, India. *J Obstet Gynaecol Res*. 2019;45(4):841-848.

159. Sallam HF, Shady NW. Reducing Blood Loss During Abdominal Hysterectomy with Intravenous Versus Topical Tranexamic Acid: A Double-Blind Randomized Controlled Trial. *J Obstet Gynaecol India*. 2019;69(2):173-179.
160. Sankar D, Krishnan R, Veerabahu M, Vikraman B. Evaluation of the efficacy of tranexamic acid on blood loss in orthognathic surgery. A prospective, randomized clinical study. *Int J Oral Maxillofac Surg*. 2012;41(6):713-7.
161. Santos ATL, Kalil RAK, Bauemann C, Pereira JB, Nesralla IA. A randomized, double-blind, and placebo-controlled study with tranexamic acid of bleeding and fibrinolytic activity after primary coronary artery bypass grafting. *Brazilian Journal of Medical and Biological Research*. 2006;39(1):63-69.
162. Seddighi A, Nikouei A, Seddighi AS, et al. The role of tranexamic acid in prevention of hemorrhage in major spinal surgeries. *Asian J Neurosurg*. 2017;12(3):501-505.
163. Sentilhes L, Winer N, Azria E, et al. Tranexamic acid for the prevention of blood loss after vaginal delivery. *New England Journal of Medicine*. 2018;379(8):731-742.
164. Senturk MB, Cakmak Y, Yildiz G, Yildiz P. Tranexamic acid for cesarean section: a double-blind, placebo-controlled, randomized clinical trial. *Arch Gynecol Obstet*. 2013;287(4):641-5.
165. Seo JG, Moon YW, Park SH, Kim SM, Ko KR. The comparative efficacies of intra-articular and IV tranexamic acid for reducing blood loss during total knee arthroplasty. *Knee Surg Sports Traumatol Arthrosc*. 2013;21(8):1869-74.
166. Seol YJ, Seon JK, Lee SH, et al. Effect of Tranexamic Acid on Blood Loss and Blood Transfusion Reduction after Total Knee Arthroplasty. *Knee Surg Relat Res*. 2016;Sep 28(3):188-93.
167. Seviciu A, Gross I, Fathima S, Walsh SM. Effects of tranexamic acid and bipolar sealer alone or in combination in primary total knee arthroplasty: a prospective, randomized, controlled trial. *Arthroplast Today*. 2016;2(2):77-82.
168. Shaaban MM, Ahmed MR, Farhan RE, Dardeer HH. Efficacy of Tranexamic Acid on Myomectomy-Associated Blood Loss in Patients with Multiple Myomas. *Reproductive Sciences*. 2016;23(7):908-912.
169. Shahid A, Khan A. Tranexamic acid in decreasing blood loss during and after caesarean section. *J Coll Physicians Surg Pak*. 2013;23(7):459-62.
170. Shakur H, Roberts I, Bautista R, et al. Effects of tranexamic acid on death, vascular occlusive events, and blood transfusion in trauma patients with significant haemorrhage (CRASH-2): a randomised, placebo-controlled trial. *Lancet*. 2010;376(9734):23-32.

171. WOMAN Trial Collaborators. Effect of early tranexamic acid administration on mortality, hysterectomy, and other morbidities in women with post-partum haemorrhage (WOMAN): an international, randomised, double-blind, placebo-controlled trial. *Lancet*. 2017;389(10084):2105-2116.
172. Shen PF, Hou WL, Chen JB, Wang B, Qu YX. Effectiveness and safety of tranexamic acid for total knee arthroplasty: a prospective randomized controlled trial. *Med Sci Monit*. 2015;21:576-81.
173. Shi J, Ji H, Ren F, et al. Protective effects of tranexamic acid on clopidogrel before coronary artery bypass grafting: a multicenter randomized trial. *JAMA Surg*. 2013;148(6):538-47.
174. Shi J, Wang G, Lv H, et al. Tranexamic Acid in on-pump coronary artery bypass grafting without clopidogrel and aspirin cessation: randomized trial and 1-year follow-up. *Ann Thorac Surg*. 2013;95(3):795-802.
175. Shi H, Ou Y, Jiang D, Quan Z, Zhao Z, Zhu Y. Tranexamic acid reduces perioperative blood loss of posterior lumbar surgery for stenosis or spondylolisthesis: A randomized trial. *Medicine (Baltimore)*. 2017;96(1):e5718.
176. Shimizu K, Toda Y, Iwasaki T, et al. Effect of tranexamic acid on blood loss in pediatric cardiac surgery: a randomized trial. *J Anesth*. 2011;25(6):823-30.
177. Shore-Lesserson L, Reich DL, Vela-Cantos F, Ammar T, Arisan Ergin M. Tranexamic acid reduces transfusions and mediastinal drainage in repeat cardiac surgery. *Anesthesia and Analgesia*. 1996;83(1):18-26.
178. Spitler CA, Row ER, Gardner WE, 2nd, et al. Tranexamic Acid Use in Open Reduction and Internal Fixation of Fractures of the Pelvis, Acetabulum, and Proximal Femur: A Randomized Controlled Trial. *J Orthop Trauma*. 2019;33(8):371-376.
179. Sprigg N, Renton CJ, Dineen RA, Kwong Y, Bath PM. Tranexamic acid for spontaneous intracerebral hemorrhage: a randomized controlled pilot trial (ISRCTN50867461). *J Stroke Cerebrovasc Dis*. 2014;23(6):1312-8.
180. Sprigg N, Flaherty K, Appleton JP, et al. Tranexamic acid for hyperacute primary IntraCerebral Haemorrhage (TICH-2): an international randomised, placebo-controlled, phase 3 superiority trial. *The Lancet*. 2018;391(10135):2107-2115.
181. Stowers MDJ, Aoina J, Vane A, Poutawera V, Hill AG, Munro JT. Tranexamic Acid in Knee Surgery Study—A Multicentered, Randomized, Controlled Trial. *Journal of Arthroplasty*. 2017;32(11):3379-3384.
182. Sun Q, Yu X, Wu J, Ge W, Cai M, Li S. Efficacy of a Single Dose and an Additional Dose of Tranexamic Acid in Reduction of Blood Loss in Total Knee Arthroplasty. *J Arthroplasty*. 2017;32(7):2108-2112.

183. Taghaddomi RJ, Mirzaee A, Attar AS, Shirdel A. Tranexamic acid reduces blood loss in off-pump coronary artery bypass surgery. *J Cardiothorac Vasc Anesth*. 2009;23(3):312-5.
184. Tengberg PT, Foss NB, Palm H, Kallemose T, Troelsen A. Tranexamic acid reduces blood loss in patients with extracapsular fractures of the hip: results of a randomised controlled trial. *Bone Joint J*. 2016;98(6):747-53.
185. Thipparampall AK, Gurajala I, Gopinath R. The effect of different dose regimens of tranexamic acid in reducing blood loss during hip surgery. *Indian J Anaesth*. 2017;61(3):235-239.
186. Tian S, Shen Z, Liu Y, Zhang Y, Peng A. The effect of tranexamic acid on hidden bleeding in older intertrochanteric fracture patients treated with PFNA. *Injury*. 2018;49(3):680-684.
187. Topsoee MF, Bergholt T, Ravn P, et al. Anti-hemorrhagic effect of prophylactic tranexamic acid in benign hysterectomy-a double-blinded randomized placebo-controlled trial. *Am J Obstet Gynecol*. 2016;215(1):72.e1-8.
188. Tsementzis SA, Hitchcock ER, Meyer CH. Benefits and risks of antifibrinolytic therapy in the management of ruptured intracranial aneurysms. A double-blind placebo-controlled study. *Acta Neurochir (Wien)*. 1990;102(1):1-10.
189. Tsutsumimoto T, Shimogata M, Ohta H, Yui M, Yoda I, Misawa H. Tranexamic acid reduces perioperative blood loss in cervical laminoplasty: a prospective randomized study. *Spine (Phila Pa 1976)*. 2011;36(23):1913-8.
190. Vanek T, Jares M, Fajt R, et al. Fibrinolytic inhibitors in off-pump coronary surgery: a prospective, randomized, double-blind TAP study (tranexamic acid, aprotinin, placebo). *Eur J Cardiothorac Surg*. 2005;28(4):563-8.
191. Vara AD, Koueiter DM, Pinkas DE, Gowda A, Wiater BP, Wiater JM. Intravenous tranexamic acid reduces total blood loss in reverse total shoulder arthroplasty: a prospective, double-blinded, randomized, controlled trial. *J Shoulder Elbow Surg*. 2017;26(8):1383-1389.
192. Veien M, Sorensen JV, Madsen F, Juelsgaard P. Tranexamic acid given intraoperatively reduces blood loss after total knee replacement: a randomized, controlled study. *Acta Anaesthesiol Scand*. 2002;46(10):1206-11.
193. Vel R, Udupi BP, Prakash MVSS, Adinarayanan S, Mishra S, Babu L. Effect of low dose tranexamic acid on intra-operative blood loss in neurosurgical patients. *Saudi Journal of Anaesthesia*. 2015;9(1):42-48.

194. Vela E, Alvarez JC, Vollmer I, Maiques J, Puig L, Santiveri FX. Tranexamic acid reduces blood transfusion without increases in thrombotic complications in joint replacement surgery. *European Journal of Anaesthesiology*. 2012;29:96.
195. Verma K, Errico T, Diefenbach C, et al. The relative efficacy of antifibrinolytics in adolescent idiopathic scoliosis: a prospective randomized trial. *J Bone Joint Surg Am*. 2014;96(10):e80.
196. Vermeulen M, Lindsay KW, Murray GD, et al. Antifibrinolytic treatment in subarachnoid hemorrhage. *N Engl J Med*. 1984;311(7):432-7.
197. Vijay BS, Bedi V, Mitra S, Das B. Role of tranexamic acid in reducing postoperative blood loss and transfusion requirement in patients undergoing hip and femoral surgeries. *Saudi J Anaesth*. 2013;7(1):29-32.
198. Volquind D, Zardo RA, Winkler BC, Londero BB, Zanelatto N, Leichtweis GP. Use of tranexamic acid in primary total knee replacement: effects on perioperative blood loss. *Braz J Anesthesiol*. 2016;66(3):254-8.
199. von Holstein CC, Eriksson SB, Kallen R. Tranexamic acid as an aid to reducing blood transfusion requirements in gastric and duodenal bleeding. *Br Med J (Clin Res Ed)*. 1987;294(6563):7-10.
200. Wang C, Kang P, Ma J, Yue C, Xie J, Pei F. Single-dose tranexamic acid for reducing bleeding and transfusions in total hip arthroplasty: A double-blind, randomized controlled trial of different doses. *Thromb Res*. 2016;141:119-23.
201. Wang G, Xie G, Jiang T, et al. Tranexamic acid reduces blood loss after off-pump coronary surgery: a prospective, randomized, double-blind, placebo-controlled study. *Anesth Analg*. 2012;115(2):239-43.
202. Wang JW, Chen B, Lin PC, Yen SH, Huang CC, Kuo FC. The Efficacy of Combined Use of Rivaroxaban and Tranexamic Acid on Blood Conservation in Minimally Invasive Total Knee Arthroplasty a Double-Blind Randomized, Controlled Trial. *J Arthroplasty*. 2017;32(3):801-806.
203. Wang Q, Liu J, Fan R, et al. Tranexamic acid reduces postoperative blood loss of degenerative lumbar instability with stenosis in posterior approach lumbar surgery: a randomized controlled trial. *Eur Spine J*. 2013;22(9):2035-8.
204. Wang W, Duan K, Ma M, et al. Tranexamic Acid Decreases Visible and Hidden Blood Loss Without Affecting Prethrombotic State Molecular Markers in Transforaminal Thoracic Interbody Fusion for Treatment of Thoracolumbar Fracture-Dislocation. *Spine (Phila Pa 1976)*. 2018;43(13):E734-e739.

205. Watts CD, Houdek MT, Sems SA, Cross WW, Pagnano MW. Tranexamic Acid Safely Reduced Blood Loss in Hemi- and Total Hip Arthroplasty for Acute Femoral Neck Fracture: A Randomized Clinical Trial. *J Orthop Trauma*. 2017;31(7):345-351.
206. Wei M, Jian K, Guo Z, et al. Tranexamic acid reduces postoperative bleeding in off-pump coronary artery bypass grafting. *Scand Cardiovasc J*. 2006;40(2):105-9.
207. Wong J, El Beheiry H, Rampersaud YR, et al. Tranexamic Acid reduces perioperative blood loss in adult patients having spinal fusion surgery. *Anesth Analg*. 2008;107(5):1479-86.
208. Wu CC, Ho WM, Cheng SB, et al. Perioperative parenteral tranexamic acid in liver tumor resection: A prospective randomized trial toward "blood transfusion"-free hepatectomy. *Annals of Surgery*. 2006;243(2):173-180.
209. Xie B, Tian J, Zhou DP. Administration of Tranexamic Acid Reduces Postoperative Blood Loss in Calcaneal Fractures: A Randomized Controlled Trial. *J Foot Ankle Surg*. 2015;54(6):1106-10.
210. Xu C, Wu A, Yue Y. Which is more effective in adolescent idiopathic scoliosis surgery: batroxobin, tranexamic acid or a combination? *Arch Orthop Trauma Surg*. 2012;132(1):25-31.
211. Xu J, Gao W, Ju Y. Tranexamic acid for the prevention of postpartum hemorrhage after cesarean section: a double-blind randomization trial. *Arch Gynecol Obstet*. 2013;287(3):463-8.
212. Xu X, Jiang J, Liu W, Li X, Lu H. Application of thromboelastography to evaluate the effect of different routes administration of tranexamic acid on coagulation function in total hip arthroplasty. *J Orthop Surg Res*. 2019;14(1):430.
213. Yamasaki S, Masuhara K, Fuji T. Tranexamic acid reduces blood loss after cementless total hip arthroplasty-prospective randomized study in 40 cases. *Int Orthop*. 2004;28(2):69-73.
214. Yamasaki S, Masuhara K, Fuji T. Tranexamic acid reduces postoperative blood loss in cementless total hip arthroplasty. *J Bone Joint Surg Am*. 2005;87(4):766-70.
215. Yanartas M, Baysal A, Aydin C, et al. The effects of tranexamic acid and 6% hydroxyethyl starch (HES) solution (130/0.4) on postoperative bleeding in coronary artery bypass graft (CABG) surgery. *Int J Clin Exp Med*. 2015;8(4):5959-71.
216. Ye X, Chen Y, Lin K, Zheng Y, Jiang C. Clinical study of the optimal dose of intravenous tranexamic acid guided by thrombelastogram during the perioperative period of total knee arthroplasty. *International Journal of Clinical and Experimental Medicine*. 2019;12(6):6989-6998.

217. Yen SH, Lin PC, Chen B, Huang CC, Wang JW. Topical Tranexamic Acid Reduces Blood Loss in Minimally Invasive Total Knee Arthroplasty Receiving Rivaroxaban. *Biomed Res Int*. 2017;2017:9105645.
218. Yi Z, Bin S, Jing Y, Zongke Z, Pengde K, Fuxing P. Tranexamic Acid Administration in Primary Total Hip Arthroplasty: A Randomized Controlled Trial of Intravenous Combined with Topical Versus Single-Dose Intravenous Administration. *J Bone Joint Surg Am*. 2016;98(12):983-91.
219. Yuan X, Li B, Wang Q, Zhang X. Comparison of 3 Routes of Administration of Tranexamic Acid on Primary Unilateral Total Knee Arthroplasty: A Prospective, Randomized, Controlled Study. *J Arthroplasty*. 2017;32(9):2738-2743.
220. Yutthakasemsunt S, Kittiwatanagul W, Piyavechvirat P, Thinkamrop B, Phuenpathom N, Lumbiganon P. Tranexamic acid for patients with traumatic brain injury: a randomized, double-blinded, placebo-controlled trial. *BMC Emerg Med*. 2013;13:20.
221. Zabeeda D, Medalion B, Sverdlov M, et al. Tranexamic acid reduces bleeding and the need for blood transfusion in primary myocardial revascularization. *Ann Thorac Surg*. 2002;74(3):733-8.
222. Zaman SU, Zakir I, Faraz Q, Akhtar S, Nawaz A, Adeel M. Effect of single-dose intravenous tranexamic acid on postoperative nasal bleed in septoplasty. *Eur Ann Otorhinolaryngol Head Neck Dis*. 2019;136(6):435-438.
223. Zekcer A, Del Priori R, Tieppo C, da Silva RS, Severino NR. Topical vs. intravenous administration of tranexamic acid in knee arthroplasty and prevalence of deep venous thrombosis: a randomized clinical trial. *J Vasc Bras*. 2016;15(2):120-125.
224. Zhang S, Xie J, Cao G, Lei Y, Huang Q, Pei F. Six-Dose Intravenous Tranexamic Acid Regimen Further Inhibits Postoperative Fibrinolysis and Reduces Hidden Blood Loss following Total Knee Arthroplasty. *J Knee Surg*. 2021;34(2):224-232.
225. Zhang S, Xu H, Xie J, Cao G, Lei Y, Pei F. Tranexamic acid attenuates inflammatory effect and modulates immune response in primary total knee arthroplasty: a randomized, placebo-controlled, pilot trial. *Inflammopharmacology*. 2020;28(4):839-849.
226. Zhang S, Xiao C, Yu W, Long N, He F, Cai P, Luo K, Jiang Y. Tranexamic acid safely reduces hidden blood loss in patients undergoing intertrochanteric fracture surgery: a randomized controlled trial. Eur J Trauma Emerg Surg. 2020 May 15. doi: 10.1007/s00068-020-01387-0.

227. Zhang Y, Zhang L, Ma X, et al. What is the optimal approach for tranexamic acid application in patients with unilateral total hip arthroplasty? *Orthopade*. 2016;45(7):616-21.
228. Zhang Y, Gao X, Yuan S, et al. Effects of tranexamic acid on short-term and long-term outcomes of on-pump coronary artery bypass grafting: Randomized trial and 7-year follow-up. *Cardiovasc Ther*. 2018;36(6):e12472.
229. Zhao HY, Xiang MY, Xia YY, Shi X, Pei FX, Kang PD. Efficacy of oral tranexamic acid on blood loss in primary total hip arthroplasty using a direct anterior approach: a prospective randomized controlled trial. *International Orthopaedics*. 2018;42(11):2535-2542.
230. Zhou KD, Wang HY, Wang Y, Liu ZH, He C, Feng JM. Is topical or intravenous tranexamic acid preferred in total hip arthroplasty? A randomized, controlled, noninferiority clinical trial. *PLoS One*. 2018;13(10):e0204551.
231. Zhou XD, Zhang Y, Jiang LF, et al. Efficacy and Safety of Tranexamic Acid in Intertrochanteric Fractures: A Single-Blind Randomized Controlled Trial. *Orthop Surg*. 2019;11(4):635-642.
232. Zhu X, Shi Q, Li D, Wu J, Guo K, Zheng X, Li H. Two Doses of Tranexamic Acid Reduce Blood Loss in Primary Posterior Lumbar Fusion Surgery: A Randomized-controlled Trial. Clin Spine Surg. 2020;33(10):E593-E597.
233. Zohar E, Ellis M, Ifrach N, Stern A, Sapir O, Fredman B. The postoperative blood-sparing efficacy of oral versus intravenous tranexamic acid after total knee replacement. *Anesth Analg*. 2004;99(6):1679-83, table of contents.
234. Zufferey PJ, Miquet M, Quenet S, et al. Tranexamic acid in hip fracture surgery: a randomized controlled trial. *Br J Anaesth*. 2010;104(1):23-30.
