# Supplementary material for: Effect of tranexamic acid on thrombotic events and seizures in bleeding patients: a systematic review and meta-analysis
Source: Crit Care. 2021 Nov 1;25:380. doi: 10.1186/s13054-021-03799-9 (PMC8561958; doi:10.1186/s13054-021-03799-9)

Online Supplementary Figure

Table of Contents

Figure S1. Risk of bias item presented as percentages 2

Figure S2. Risk of bias summary and graph 3

Figure S3. Funnel plot of the thrombotic events 5

Figure S4. Funnel plot of the seizures 6

Figure S5. Forest plot of the thrombotic events: subgroup analysis by underlying disease 7

Figure S6. Forest plot of the thrombotic events: subgroup analysis by TXA dose 11

Figure S7. Forest plot of the thrombotic events: subgroup analysis in children. 14

Figure S8. Forest plot of the seizures: subgroup analysis by underlying disease 15

Figure S9. Forest plot of the seizures: subgroup analysis by TXA dose 17

Figure S10. Forest plot of the seizures: subgroup analysis in children 18

Figure S11. Forest plot of the venous thromboembolism: subgroup analysis by underlying disease 19

Figure S12. Forest plot of the acute coronary syndrome: subgroup analysis by underlying disease 23

Figure S13. Forest plot of the stroke: subgroup analysis by underlying disease severity 25

Figure S1. Risk of bias item presented as percentages across all included studies.

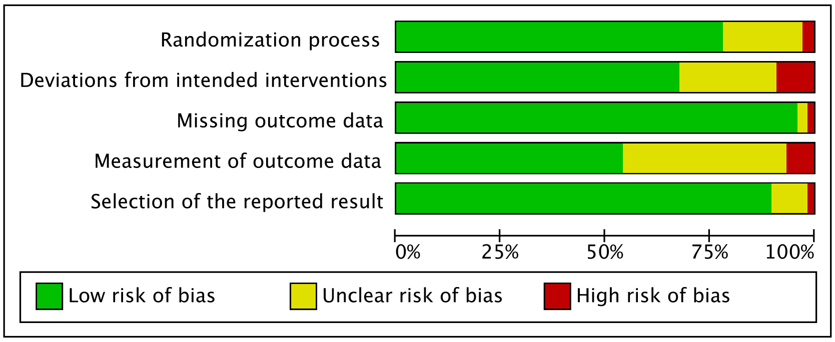

Figure S2. Risk of bias summary and graph.

Study	Randomization process	Deviations from intended interventions	Missing outcome data	Measurement of outcome data	Selection of the reported result
Abdel-Aleem 2013	−	−	+	−	−
Abdul 2019	+	+	+	+	+
Aguilera 2013	+	−	+	+	+
Aguilera 2015	+	+	?	−	+
Ahmed 2015	+	−	+	−	−
Ahn 2012	+	+	+	+	+
Akgul 2016	?	?	+	?	?
Alimian 2011	+	+	+	+	+
Alizadeh Ghavidel 2015	+	+	+	?	+
Altun 2017	?	?	+	?	+
Alvarez 2008	+	+	+	+	+
Alvarez 2019	+	+	+	?	+
Andreasen 2004	+	?	?	?	?
Apipan 2018	+	+	+	?	+
Armellin 2001	?	?	−	?	−
Auvinen 1987	?	?	+	?	+
Avvisati 1989	?	?	+	?	+
Barrachina 2016	+	+	+	+	+
Baruah 2016	?	−	+	−	+
Batibay 2018	?	?	?	?	?
Benoni 1996	+	+	+	+	+
Benoni 2000	+	+	+	+	+
Benoni 2001	+	+	+	?	+
Bidolegui 2014	+	−	+	−	?
Boylan 1996	+	+	+	+	+
Brown 1997	+	?	+	?	+
Bulutcu 2005	?	?	+	+	+
Caglar 2008	+	+	+	+	+
Cansancao 2018	−	?	+	?	+
Carabini 2017	+	+	+	+	+
Casati 2001	+	+	+	?	+
Casati 2002	?	+	+	?	+
Casati 2004	+	+	+	+	+
Chakravarthy 2012	+	?	+	?	+
Chakroun-Walha 2019	+	−	+	−	+
Chandra 1978	?	+	+	?	+
Chareancholvanich 2012	+	+	+	+	+
Charoencholvanich 2011	+	+	+	?	+
Chauhan 2003	+	?	+	+	+
Chauhan 2004	+	?	+	?	+
Chen 2016	+	−	+	+	+
Chen 2019	+	+	+	+	+
Chin 2020	+	+	+	?	+
Choi 2009	+	+	?	+	+
Christabel 2014	+	+	+	+	+
Claeys 2007	?	?	+	+	+
Clave 2019	+	+	+	+	+
Cohen 2021	+	+	+	+	+
Colomina 2016	+	+	+	+	+
Crescenti 2011	+	+	+	+	+
Cvetanovich 2018	+	+	+	+	+
Dadure 2011	+	+	+	+	+
Diprose 2005	+	+	+	+	+
Ducloy-Bouthors 2011	+	−	+	+	+
Ebrahimi 2019	+	+	+	?	+
Ekback 2000	?	?	+	?	?
Eldaba 2013	+	+	+	+	+
Elwatidy 2008	+	+	+	+	+
Emara 2014	?	?	+	?	+
Engel 2001	?	?	+	?	?
Engqvist 1979	?	?	+	?	+
Esfandiari 2012	?	+	+	+	+
Ezhevskaya 2018	+	+	+	?	+
Faraoni 2014	?	?	+	?	+
Farrokhi 2011	+	+	+	+	+
Felli 2019	+	+	+	+	+
Fenger-Eriksen 2019	+	+	+	+	+
Fodstad 1981	?	−	+	−	+
Fraval 2017	+	+	+	+	+
Gai 2004	+	+	+	+	+
Garg 2012	?	?	?	?	?
Garneti 2004	+	+	+	?	+
Goobie 2011	+	+	+	+	+
Goobie 2018	+	+	+	+	+
Good 2003	+	?	+	?	+
Goswami 2013	+	+	+	?	+
Greiff 2012	?	?	+	?	+
Gungorduk 2011	+	?	+	?	+
Gungorduk 2013	+	?	+	?	+
Gupta 2012	+	+	+	?	+
Hardy 1998	+	?	?	?	+
Helito 2019	+	+	+	+	+
Hiippala 1995	+	+	+	+	+
Hiippala 1997	+	+	+	?	+
Hillman 2002	?	−	+	?	?
Hooda 2015	+	+	+	+	+
Horrow 1991	+	+	+	+	+
Horrow 1995	+	+	+	?	+
Hsu 2015	+	+	+	?	+
Husted 2003	+	+	+	+	+
Jansen 1999	+	+	+	?	+
Jares 2003	?	?	+	?	+
Jaszczyk 2015	+	?	+	?	+
Johansson 2005	+	+	+	?	+
Jokar 2017	+	?	+	+	+
Kakar 2009	+	+	+	+	+
Karaaslan 2015	+	+	+	+	+
Karski 1995	?	?	+	?	+
Karski 2005	+	?	+	?	+
Kaste 1979	+	+	+	+	+
Katsaros 1996	?	?	+	?	+
Kazemi 2010	?	+	+	?	+
Keyhani 2016	?	?	+	?	?
Kim 2014	+	+	+	+	+
Kim 2017	+	+	+	+	+
Kimura 2019	+	+	+	+	+
Kuitunen 2005	+	?	+	?	+
Kulkarni 2016	+	+	+	+	+
Kundu 2015	+	+	+	+	+
Lack 2017	?	+	+	?	+
Later 2009	+	+	+	+	+
Lee 2012	+	+	+	+	+
Lee 2013	+	+	+	+	+
Lei 2020	?	+	+	+	+
Lemay 2004	+	+	+	+	+
Lin 2012	+	+	+	+	+
Liu 2018	?	?	+	?	?
Liu 2020	+	+	+	?	+
Lundin 2014	+	+	+	+	+
Luo 2019	+	+	+	+	+
Ma 2019	+	+	+	+	+
MacGillivray 2011	+	+	+	?	+
Maddali 2007	+	+	+	+	+
Maged 2015	+	−	+	−	+
Mahmood 2017	−	−	+	−	−
Maniar 2012	+	+	+	+	+
Mansouri 2012	?	?	+	?	+
Mehr-Aein 2007	?	+	+	?	+
Meng 2019	+	+	+	+	+
Misfeld 1998	?	?	+	?	+
Molloy 2005	+	+	+	+	+
Monaco 2020	+	+	+	+	+
Motififard 2015	+	+	+	+	+
Mu 2019	+	−	−	−	?
Myles 2016	+	+	+	+	+
Na 2016	+	+	+	+	+
Nagabhushan 2018	+	+	+	+	+
Neilipovitz 2001	?	+	+	+	+
Nejad 2011	?	+	+	?	+
Niskanen 2005	+	+	+	?	+
Nugent 2019	+	+	+	+	+
Oremus 2014	+	+	+	+	+
Orpen 2006	+	+	+	?	+
Painter 2018	+	+	+	?	+
Palija 2020	+	+	+	?	+
Peters 2015	+	+	+	?	+
Pleym 2003	+	?	+	?	+

Prakash 2017	+	+	+	+	+
Prasad 2018	+	+	+	+	+
Raksakietisak 2015	+	+	+	+	+
Ramezani 2005	?	+	+	+	+
Ray 2016	+	?	+	?	+
Reid 1997	+	+	+	+	+
Roberts 2019	+	+	+	+	+
Roberts 2020	+	+	+	+	+
Ross 2000	+	+	+	+	+
Rowell 2020	+	+	−	+	+
Sahu 2019	−	−	+	−	+
Sallam 2019	+	+	+	+	+
Sankar 2012	+	+	+	+	+
Santos 2006	+	+	+	?	+
Seddighi 2019	?	?	+	?	+
Sentilhes 2018	+	+	+	+	+
Senturk 2013	−	−	+	?	?
Seo 2013	+	?	+	?	+
Seol 2016	+	?	+	?	+
Seviciu 2016	+	+	+	?	+
Shaaban 2016	+	−	+	?	?
Shahid 2013	+	+	+	+	+
Shakur 2010	+	+	+	+	+
Shakur 2017	+	+	+	+	+
Shen 2015	+	+	+	+	+
Shi, J 2013	+	+	+	+	+
Shi 2013	+	+	+	+	+
Shi 2017	+	+	+	+	+
Shimizu 2011	+	+	+	+	+
Shore-Lesserson 1996	+	?	+	?	+
Spitler 2019	+	?	+	?	+
Sprigg 2014	+	?	+	?	+
Sprigg 2018	+	+	+	+	+
Stowers 2017	+	+	+	+	+
Sun 2017	+	?	+	?	+
Taghaddomi 2009	+	+	+	+	+
Tengberg 2016	+	+	+	+	+
Thipparampall 2020	+	+	+	−	+
Tian 2018	+	?	+	?	+
Topsoee 2016	+	+	+	+	+
Tsementzis 1990	?	+	+	?	+
Tsutsumimoto 2011	−	−	+	−	+
Vanek 2005	+	+	+	?	+
Vara 2017	?	+	−	+	+
Veien 2002	+	−	+	−	?
Vel 2015	+	+	+	+	+
Vela 2012	?	?	+	?	?
Verma 2014	+	+	+	+	+
Vermeulen 1984	+	+	+	+	+
Vijay 2013	?	+	+	?	+
Volquind 2014	+	+	+	+	+
von Holstein 1987	+	+	+	+	+
Wang, J 2016	+	+	+	+	+
Wang 2012	+	+	+	+	+
Wang 2013	+	?	+	?	+
Wang 2016	+	+	+	+	+
Wang 2018	+	+	+	+	+
Watts 2017	+	+	+	+	+
Wei 2006	?	?	+	?	+
Wong 2008	+	+	+	+	+
Wu 2006	+	+	+	?	+
Xie 2015	+	+	+	+	+
Xu 2012	?	?	+	?	?
Xu 2013	+	?	+	?	+
Xu 2019	+	+	+	+	+
Yamasaki 2004	+	+	+	?	+
Yamasaki 2005	?	?	+	?	?
Yanartas 2015	+	−	+	+	+
Ye 2019	?	?	+	?	?
Yen 2017	+	+	+	+	+
Yi 2016	+	+	+	+	+
Yuan 2017	+	+	+	+	+
Yutthakasemsunt 2013	+	+	+	+	+
Zabeeda 2002	?	?	+	?	+
Zaman 2019	+	+	+	?	+
Zekcer 2016	+	−	+	−	?
Zhang 2016	+	?	+	?	+
Zhang 2018	+	+	+	+	+
Zhang 2020	+	+	+	+	+
Zhang 2021	+	+	+	+	+
Zhang S 2020	+	+	+	+	+
Zhao 2018	+	+	+	+	+
Zhou 2018	+	+	+	+	+
Zhou 2019	−	+	+	+	+
Zhu 2020	+	+	+	+	+
Zohar 2004	+	−	+	−	?
Zufferey 2010	+	+	+	+	+

Figure S3. Funnel plot of the thrombotic events.

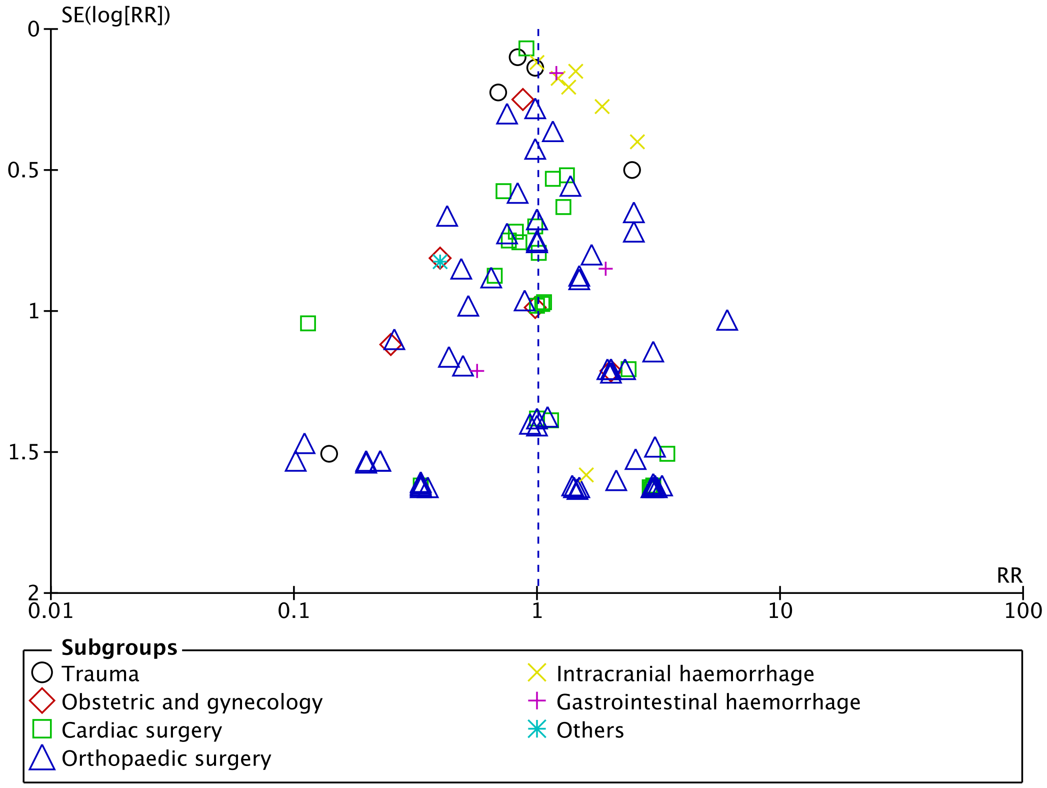

Figure S4. Funnel plot of the seizures.

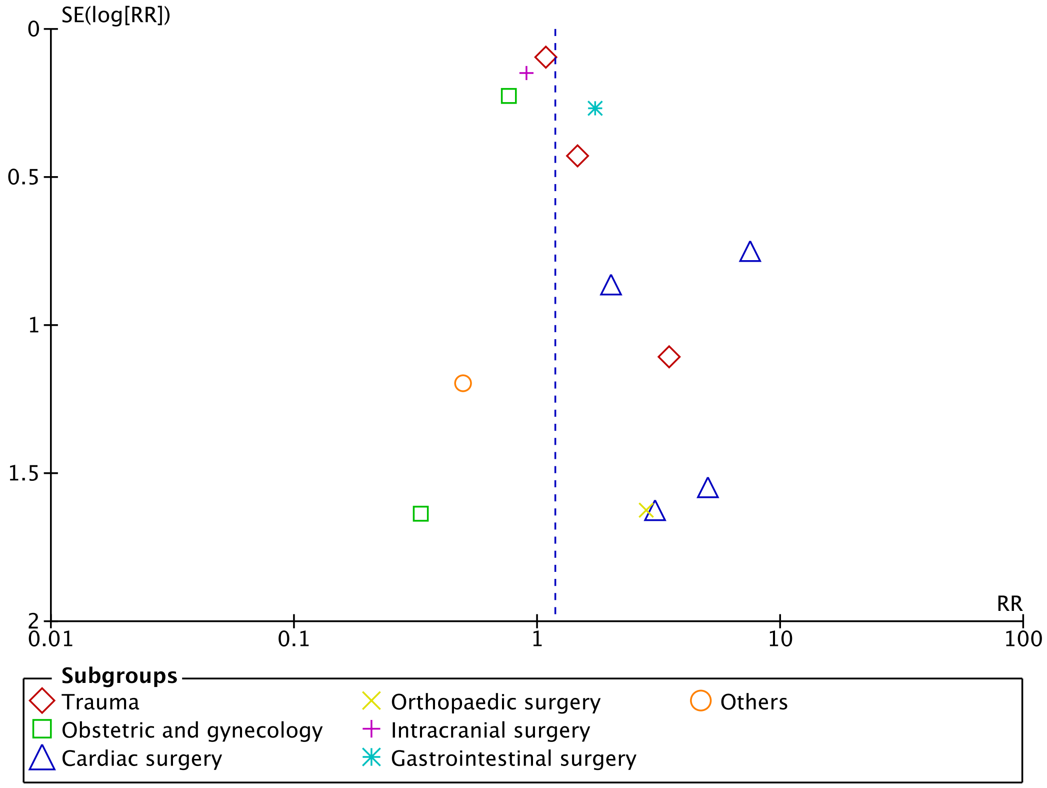

Figure S5. Forest plot of the thrombotic events: subgroup analysis by underlying disease.

Study or Subgroup	Experimental Events	Experimental Total	Control Events	Control Total	Weight	Risk Ratio M-H, Random, 95% CI
**1.1.1 Trauma**						
Chakroun-Walha 2019	14	96	5	84	0.6%	2.45 [0.92, 6.52]
Ebrahimi 2019	0	40	0	40		Not estimable
Jokar 2017	0	40	0	40		Not estimable
Roberts 2019	101	6406	102	6331	7.1%	0.98 [0.74, 1.29]
Rowell 2020	44	657	30	309	2.7%	0.69 [0.44, 1.08]
Shakur 2010	168	10093	201	10114	12.8%	0.84 [0.68, 1.03]
Yutthakasemsunt 2013	0	120	3	118	0.1%	0.14 [0.01, 2.69]
**Subtotal (95% CI)**		**17452**		**17036**	**23.1%**	**0.89 [0.69, 1.17]**
Total events	327		341			
Heterogeneity: Tau² = 0.04; Chi² = 7.66, df = 4 (P = 0.11); I² = 48%						
Test for overall effect: Z = 0.82 (P = 0.41)						
**1.1.2 Obstetric and gynecology**						
Abdel-Aleem 2013	0	373	0	367		Not estimable
Abdul 2019	0	40	0	40		Not estimable
Ahmed 2015	0	62	0	62		Not estimable
Caglar 2008	0	50	0	50		Not estimable
Ducloy-Bouthors 2011	2	72	1	72	0.1%	2.00 [0.19, 21.57]
Gai 2004	0	91	0	89		Not estimable
Goswami 2013	0	60	0	30		Not estimable
Gungorduk 2011	0	330	0	330		Not estimable
Gungorduk 2013	0	220	0	219		Not estimable
Gupta 2012	0	30	0	30		Not estimable
Lundin 2014	2	50	5	50	0.2%	0.40 [0.08, 1.97]
Maged 2015	0	100	0	100		Not estimable
Ray 2016	0	50	0	50		Not estimable
Sahu 2019	0	50	0	50		Not estimable
Sallam 2019	0	43	0	43		Not estimable
Sentilhes 2018	1	1945	4	1946	0.1%	0.25 [0.03, 2.24]
Senturk 2013	0	101	0	122		Not estimable
Shaaban 2016	0	66	0	66		Not estimable
Shahid 2013	0	38	0	36		Not estimable
Shakur 2017	30	10051	34	10009	2.2%	0.88 [0.54, 1.43]
Topsoee 2016	0	165	0	167		Not estimable
Xu 2013	2	88	2	86	0.1%	0.98 [0.14, 6.78]
**Subtotal (95% CI)**		**14075**		**14014**	**2.7%**	**0.81 [0.53, 1.26]**
Total events	37		46			
Heterogeneity: Tau² = 0.00; Chi² = 2.56, df = 4 (P = 0.63); I² = 0%						
Test for overall effect: Z = 0.92 (P = 0.36)						
**1.1.3 Cardiac surgery**						
Ahn 2012	1	38	0	38	0.1%	3.00 [0.13, 71.40]
Alizadeh Ghavidel 2015	8	100	6	100	0.5%	1.33 [0.48, 3.70]
Altun 2017	0	34	0	20		Not estimable
Andreasen 2004	0	21	0	23		Not estimable
Armellin 2001	1	143	0	140	0.1%	2.94 [0.12, 71.50]
Brown 1997	0	60	0	30		Not estimable
Bulutcu 2005	0	25	0	25		Not estimable
Casati 2001	6	341	3	169	0.3%	0.99 [0.25, 3.91]
Casati 2002	2	30	3	30	0.2%	0.67 [0.12, 3.71]
Casati 2004	0	52	0	50		Not estimable
Chakravarthy 2012	0	25	0	25		Not estimable
Chauhan 2003	0	96	0	24		Not estimable
Diprose 2005	0	62	0	61		Not estimable
Esfandiari 2012	7	75	6	75	0.5%	1.17 [0.41, 3.31]
Faraoni 2014	0	24	0	12		Not estimable
Greiff 2012	3	30	4	33	0.3%	0.82 [0.20, 3.39]
Hardy 1998	2	43	2	45	0.1%	1.05 [0.15, 7.10]
Horrow 1991	2	37	1	44	0.1%	2.38 [0.22, 25.20]
Horrow 1995	0	121	0	27		Not estimable
Jares 2003	1	22	1	25	0.1%	1.14 [0.08, 17.11]
Karski 1995	3	99	0	48	0.1%	3.43 [0.18, 65.10]
Karski 2005	3	147	4	165	0.2%	0.84 [0.19, 3.70]
Katsaros 1996	3	104	4	106	0.2%	0.76 [0.18, 3.33]
Kuitunen 2005	1	20	1	20	0.1%	1.00 [0.07, 14.90]
Later 2009	1	99	9	103	0.1%	0.12 [0.01, 0.90]
Maddali 2007	0	111	0	111		Not estimable
Mansouri 2012	0	30	0	30		Not estimable
Mehr-Aein 2007	0	33	0	33		Not estimable
Misfeld 1998	0	14	0	14		Not estimable
Monaco 2020	2	50	2	50	0.1%	1.00 [0.15, 6.82]
Myles 2016	316	2311	350	2320	26.6%	0.91 [0.79, 1.04]
Nejad 2011	1	50	0	50	0.1%	3.00 [0.13, 71.92]
Pleym 2003	0	40	1	40	0.1%	0.33 [0.01, 7.95]
Reid 1997	0	20	0	21		Not estimable
Santos 2006	2	29	2	31	0.1%	1.07 [0.16, 7.10]
Shi 2013	5	274	7	278	0.4%	0.72 [0.23, 2.26]
Shi, J 2013	3	58	3	59	0.2%	1.02 [0.21, 4.83]

Risk Ratio
M-H, Random, 95% CI

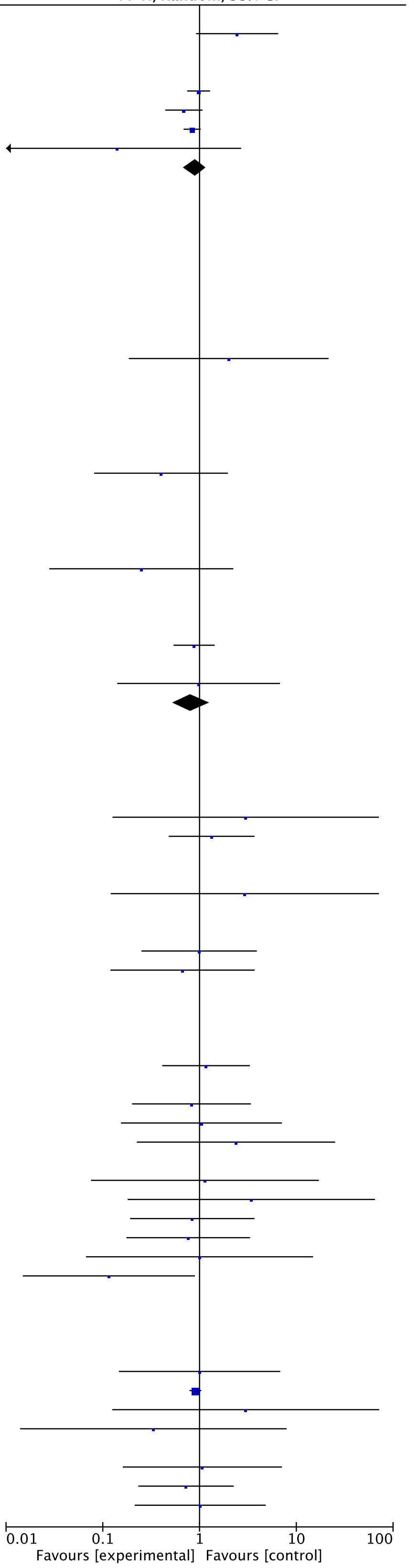

Shimizu 2011	1	81	0	79	0.1%	2.93 [0.12, 70.79]
Shore-Lesserson 1996	5	17	3	13	0.3%	1.27 [0.37, 4.39]
Taghaddomi 2009	0	50	0	50		Not estimable
Vanek 2005	0	39	0	20		Not estimable
Wang 2012	0	116	0	115		Not estimable
Wei 2006	0	36	0	40		Not estimable
Zabeeda 2002	0	25	0	25		Not estimable
Zhang 2018	0	105	0	105		Not estimable
**Subtotal (95% CI)**		**5337**		**4922**	**30.9%**	**0.92 [0.81, 1.05]**
Total events	379		412			
Heterogeneity: $Tau^2 = 0.00$; $Chi^2 = 9.34$, df = 23 (P = 0.99); $I^2 = 0\%$						
Test for overall effect: Z = 1.26 (P = 0.21)						
**1.1.4 Orthopaedic surgery**						
Aguilera 2013	1	41	0	42	0.1%	3.07 [0.13, 73.29]
Aguilera 2015	0	50	0	50		Not estimable
Akgul 2016	0	35	1	35	0.1%	0.33 [0.01, 7.91]
Alvarez 2008	0	46	0	49		Not estimable
Alvarez 2019	3	22	3	22	0.2%	1.00 [0.23, 4.42]
Barrachina 2016	2	71	2	37	0.1%	0.52 [0.08, 3.55]
Baruah 2016	0	30	0	30		Not estimable
Batibay 2018	0	35	0	35		Not estimable
Benoni 1996	4	43	4	43	0.3%	1.00 [0.27, 3.74]
Benoni 2000	3	20	3	20	0.2%	1.00 [0.23, 4.37]
Benoni 2001	1	18	1	20	0.1%	1.11 [0.07, 16.49]
Bidolegui 2014	0	25	0	25		Not estimable
Chareancholvanich 2012	0	120	0	120		Not estimable
Charoencholvanich 2011	0	50	0	50		Not estimable
Chen 2016	0	60	0	60		Not estimable
Chen 2019	14	88	12	88	1.0%	1.17 [0.57, 2.38]
Chin 2020	1	42	1	39	0.1%	0.93 [0.06, 14.34]
Claeys 2007	0	20	0	20		Not estimable
Clave 2019	1	154	0	75	0.1%	1.47 [0.06, 35.69]
Colomina 2016	2	44	1	51	0.1%	2.32 [0.22, 24.71]
Cvetanovich 2018	0	52	1	56	0.1%	0.36 [0.01, 8.61]
Ekback 2000	1	20	1	20	0.1%	1.00 [0.07, 14.90]
Elwatidy 2008	0	32	0	32		Not estimable
Emara 2014	6	20	1	20	0.1%	6.00 [0.79, 45.42]
Engel 2001	0	12	0	12		Not estimable
Ezhevskaya 2018	0	80	0	80		Not estimable
Farrokhi 2011	0	38	0	38		Not estimable
Felli 2019	0	40	0	40		Not estimable
Fraval 2017	0	50	0	51		Not estimable
Garg 2012	0	26	0	26		Not estimable
Garneti 2004	1	25	0	25	0.1%	3.00 [0.13, 70.30]
Goobie 2018	0	56	0	55		Not estimable
Good 2003	2	27	2	24	0.1%	0.89 [0.14, 5.83]
Helito 2019	0	30	1	30	0.1%	0.33 [0.01, 7.87]
Hiippala 1995	1	15	2	13	0.1%	0.43 [0.04, 4.25]
Hiippala 1997	2	39	3	38	0.2%	0.65 [0.11, 3.67]
Hsu 2015	0	30	0	30		Not estimable
Husted 2003	0	20	0	20		Not estimable
Jansen 1999	0	21	1	21	0.1%	0.33 [0.01, 7.74]
Jaszczyk 2015	1	61	0	63	0.1%	3.10 [0.13, 74.58]
Johansson 2005	0	47	0	53		Not estimable
Kakar 2009	0	25	0	25		Not estimable
Karaaslan 2015	0	53	0	52		Not estimable
Kazemi 2010	0	32	1	32	0.1%	0.33 [0.01, 7.89]
Keyhani 2016	0	40	0	40		Not estimable
Kim 2014	0	90	0	90		Not estimable
Kim 2017	0	48	2	24	0.1%	0.10 [0.01, 2.05]
Kimura 2019	0	128	0	128		Not estimable
Kundu 2015	3	30	2	30	0.2%	1.50 [0.27, 8.34]
Lack 2017	1	42	0	46	0.1%	3.28 [0.14, 78.36]
Lee 2012	3	36	4	36	0.3%	0.75 [0.18, 3.11]
Lee 2013	0	34	0	34		Not estimable
Lei 2020	0	150	0	50		Not estimable
Lemay 2004	0	20	0	19		Not estimable
Lin 2012	1	101	0	50	0.1%	1.50 [0.06, 36.18]
Liu 2018	23	150	15	74	1.5%	0.76 [0.42, 1.36]
Liu 2020	0	37	0	35		Not estimable
Luo 2019	1	44	4	46	0.1%	0.26 [0.03, 2.25]
Ma 2019	1	62	0	62	0.1%	3.00 [0.12, 72.25]
MacGillivray 2011	2	40	0	20	0.1%	2.56 [0.13, 50.95]
Mahmood 2017	0	100	0	73		Not estimable
Maniar 2012	0	160	0	40		Not estimable
Molloy 2005	0	50	0	50		Not estimable
Motififard 2015	0	45	0	45		Not estimable
Mu 2019	0	45	0	42		Not estimable
Na 2016	0	29	0	26		Not estimable
Nagabhushan 2018	0	25	0	25		Not estimable
Neilipovitz 2001	0	22	0	18		Not estimable
Niskanen 2005	0	19	0	20		Not estimable
Nugent 2019	0	18	0	23		Not estimable

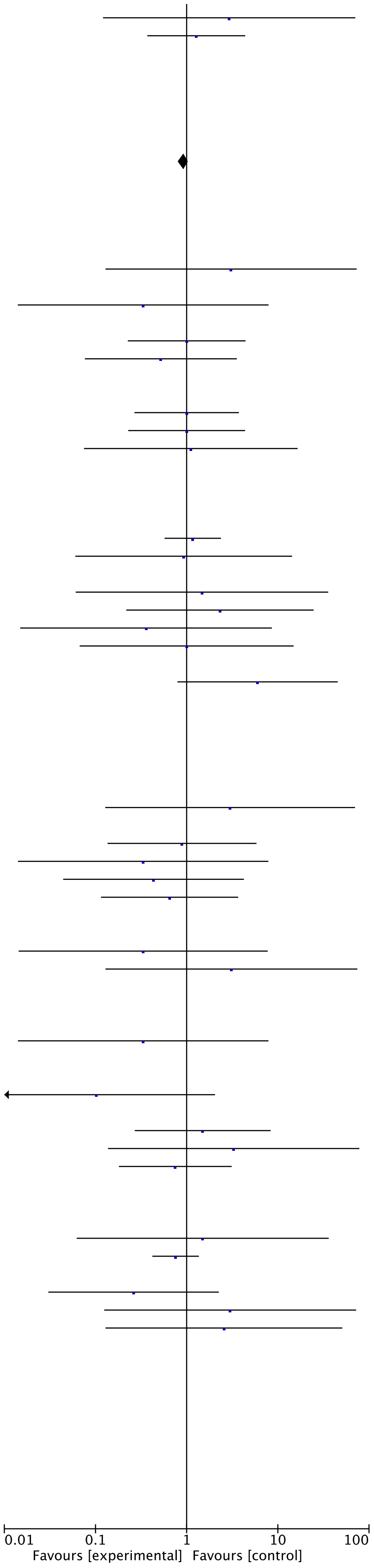

Oremus 2014	0	49	2	49	0.1%	0.20 [0.01, 4.06]
Orpen 2006	0	15	0	14		Not estimable
Painter 2018	2	71	4	69	0.2%	0.49 [0.09, 2.57]
Palija 2020	0	40	0	40		Not estimable
Peters 2015	1	19	0	13	0.1%	2.10 [0.09, 47.89]
Prakash 2017	0	50	1	50	0.1%	0.33 [0.01, 7.99]
Raksakietisak 2015	0	39	0	39		Not estimable
Sankar 2012	0	25	0	25		Not estimable
Seddighi 2019	0	20	0	20		Not estimable
Seo 2013	0	50	2	50	0.1%	0.20 [0.01, 4.06]
Seol 2016	0	50	0	50		Not estimable
Seviciu 2016	0	29	0	32		Not estimable
Shen 2015	4	46	4	46	0.3%	1.00 [0.27, 3.76]
Shi 2017	0	50	0	46		Not estimable
Spitler 2019	2	47	1	46	0.1%	1.96 [0.18, 20.85]
Stowers 2017	1	51	0	23	0.1%	1.38 [0.06, 32.76]
Sun 2017	4	135	0	45	0.1%	3.04 [0.17, 55.47]
Tengberg 2016	0	35	2	40	0.1%	0.23 [0.01, 4.59]
Thipparampall 2020	0	39	0	20		Not estimable
Tian 2018	0	50	0	50		Not estimable
Tsutsumimoto 2011	0	35	2	40	0.1%	0.23 [0.01, 4.59]
Vara 2017	3	50	2	50	0.2%	1.50 [0.26, 8.60]
Veien 2002	0	15	0	15		Not estimable
Vela 2012	3	22	3	22	0.2%	1.00 [0.23, 4.42]
Verma 2014	0	36	0	47		Not estimable
Vijay 2013	0	45	0	45		Not estimable
Volquind 2014	0	32	0	30		Not estimable
Wang 2013	0	30	0	30		Not estimable
Wang 2016	1	81	0	38	0.1%	1.43 [0.06, 34.24]
Wang 2018	15	39	16	41	1.7%	0.99 [0.57, 1.71]
Wang, J 2016	10	100	10	98	0.8%	0.98 [0.43, 2.25]
Watts 2017	5	69	6	69	0.4%	0.83 [0.27, 2.60]
Wong 2008	1	73	1	73	0.1%	1.00 [0.06, 15.69]
Xie 2015	2	45	1	45	0.1%	2.00 [0.19, 21.28]
Xu 2012	0	20	0	20		Not estimable
Xu 2019	1	68	0	67	0.1%	2.96 [0.12, 71.31]
Yamasaki 2004	0	20	0	20		Not estimable
Yamasaki 2005	0	21	0	21		Not estimable
Ye 2019	20	160	2	40	0.3%	2.50 [0.61, 10.26]
Yen 2017	0	33	0	32		Not estimable
Yi 2016	2	50	1	50	0.1%	2.00 [0.19, 21.36]
Yuan 2017	2	140	1	140	0.1%	2.00 [0.18, 21.81]
Zekcer 2016	0	30	4	30	0.1%	0.11 [0.01, 1.98]
Zhang 2016	1	25	2	25	0.1%	0.50 [0.05, 5.17]
Zhang 2020	3	61	1	61	0.1%	3.00 [0.32, 28.04]
Zhang 2021	6	106	2	59	0.2%	1.67 [0.35, 8.01]
Zhang S 2020	0	32	2	32	0.1%	0.20 [0.01, 4.01]
Zhao 2018	0	40	0	40		Not estimable
Zhou 2018	0	58	0	58		Not estimable
Zhou 2019	3	50	7	50	0.3%	0.43 [0.12, 1.56]
Zhu 2020	11	100	4	50	0.4%	1.38 [0.46, 4.10]
Zohar 2004	0	40	0	20		Not estimable
Zufferey 2010	8	57	3	53	0.3%	2.48 [0.69, 8.86]
**Subtotal (95% CI)**		**6123**		**5191**	**12.3%**	**0.98 [0.80, 1.21]**
Total events	192		154			
Heterogeneity: $Tau^2 = 0.00$; $Chi^2 = 33.74$, df = 61 (P = 1.00); $I^2 = 0\%$						
Test for overall effect: Z = 0.16 (P = 0.87)						
**1.1.5 Intracranial haemorrhage**						
Chandra 1978	0	20	0	19		Not estimable
Fodstad 1981	16	30	6	29	0.8%	2.58 [1.17, 5.66]
Hillman 2002	45	254	33	251	3.1%	1.35 [0.89, 2.04]
Kaste 1979	0	32	0	32		Not estimable
Ross 2000	83	229	84	233	9.0%	1.01 [0.79, 1.28]
Sprigg 2014	1	16	0	8	0.1%	1.59 [0.07, 35.15]
Sprigg 2018	66	1161	54	1164	4.3%	1.23 [0.86, 1.74]
Tsementzis 1990	24	50	13	50	1.7%	1.85 [1.07, 3.20]
Vermeulen 1984	79	241	54	238	6.0%	1.44 [1.07, 1.94]
**Subtotal (95% CI)**		**2033**		**2024**	**25.0%**	**1.33 [1.09, 1.63]**
Total events	314		244			
Heterogeneity: $Tau^2 = 0.02$; $Chi^2 = 9.35$, df = 6 (P = 0.15); $I^2 = 36\%$						
Test for overall effect: Z = 2.80 (P = 0.005)						
**1.1.6 Gastrointestinal haemorrhage**						
Engqvist 1979	4	76	2	73	0.2%	1.92 [0.36, 10.17]
Roberts 2020	86	5994	72	6015	5.4%	1.20 [0.88, 1.64]
von Holstein 1987	1	72	2	82	0.1%	0.57 [0.05, 6.15]
**Subtotal (95% CI)**		**6142**		**6170**	**5.7%**	**1.20 [0.89, 1.63]**
Total events	91		76			
Heterogeneity: $Tau^2 = 0.00$; $Chi^2 = 0.68$, df = 2 (P = 0.71); $I^2 = 0\%$						
Test for overall effect: Z = 1.19 (P = 0.23)						

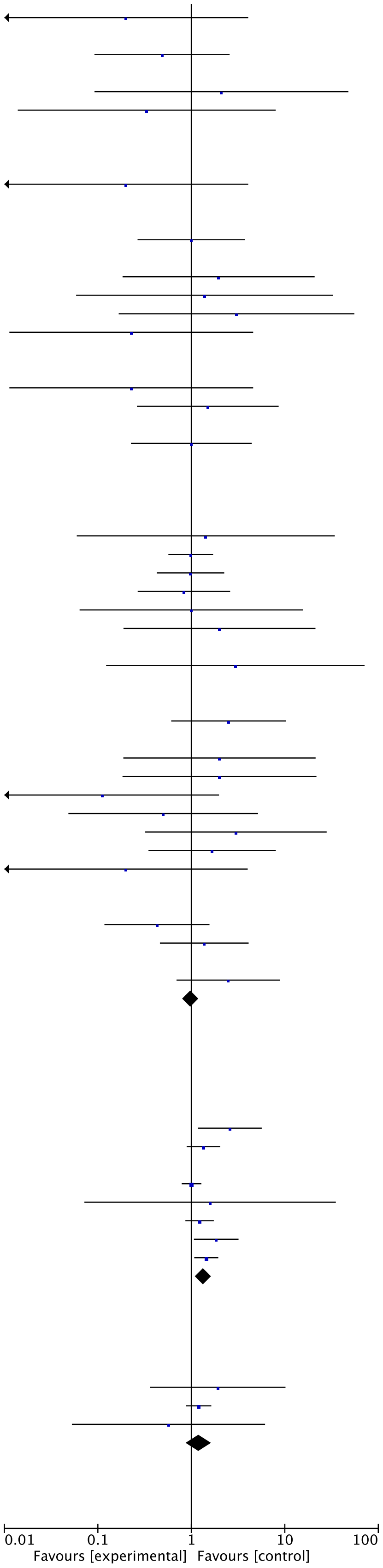

**1.1.7 Others**						
Alimian 2011	0	42	0	42		Not estimable
Apipan 2018	0	60	0	20		Not estimable
Auvinen 1987	0	39	0	37		Not estimable
Avvisati 1989	0	6	0	6		Not estimable
Boylan 1996	0	25	0	20		Not estimable
Cansancao 2018	0	10	0	10		Not estimable
Choi 2009	0	32	0	29		Not estimable
Christabel 2014	0	25	0	24		Not estimable
Cohen 2021	0	27	0	17		Not estimable
Crescenti 2011	2	100	5	100	0.2%	0.40 [0.08, 2.01]
Dadure 2011	0	19	0	20		Not estimable
Eldaba 2013	0	50	0	50		Not estimable
Fenger-Eriksen 2019	0	15	0	15		Not estimable
Goobie 2011	0	23	0	20		Not estimable
Kulkarni 2016	0	120	0	120		Not estimable
Meng 2019	0	30	0	30		Not estimable
Prasad 2018	0	40	0	20		Not estimable
Ramezani 2005	0	32	0	30		Not estimable
Vel 2015	0	50	0	50		Not estimable
Wu 2006	0	108	0	106		Not estimable
Zaman 2019	0	88	0	88		Not estimable
**Subtotal (95% CI)**		**941**		**854**	**0.2%**	**0.40 [0.08, 2.01]**
Total events	2		5			
Heterogeneity: Not applicable						
Test for overall effect: Z = 1.11 (P = 0.27)						
**Total (95% CI)**		**52103**		**50211**	**100.0%**	**1.00 [0.93, 1.08]**
Total events	1342		1278			

Heterogeneity: $Tau^2 = 0.00$; $Chi^2 = 81.71$, df = 106 (P = 0.96); $I^2 = 0\%$
Test for overall effect: Z = 0.11 (P = 0.91)
Test for subgroup differences: $Chi^2 = 13.60$, df = 6 (P = 0.03), $I^2 = 55.9\%$

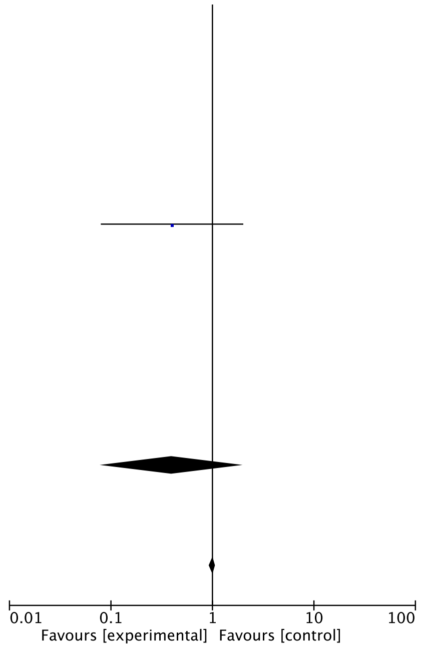

Figure S6. Forest plot of the thrombotic events: subgroup analysis by TXA (tranexamic acid) dose.

Study or Subgroup	Experimental Events	Experimental Total	Control Events	Control Total	Weight	Risk Ratio M-H, Random, 95% CI
**2.1.1 TXA dose >2g**						
Abdul 2019	0	40	0	40		Not estimable
Andreasen 2004	0	21	0	23		Not estimable
Armellin 2001	1	143	0	140	0.1%	2.94 [0.12, 71.50]
Boylan 1996	0	25	0	20		Not estimable
Bulutcu 2005	0	25	0	25		Not estimable
Chandra 1978	0	20	0	19		Not estimable
Clave 2019	1	78	0	75	0.1%	2.89 [0.12, 69.75]
Diprose 2005	0	62	0	61		Not estimable
Ducloy-Bouthors 2011	2	72	1	72	0.1%	2.00 [0.19, 21.57]
Engqvist 1979	4	76	2	73	0.2%	1.92 [0.36, 10.17]
Fodstad 1981	16	30	6	29	0.8%	2.58 [1.17, 5.66]
Goobie 2011	0	23	0	20		Not estimable
Goobie 2018	0	56	0	55		Not estimable
Hardy 1998	2	43	2	45	0.1%	1.05 [0.15, 7.10]
Hillman 2002	45	254	33	251	3.1%	1.35 [0.89, 2.04]
Horrow 1995	0	27	0	27		Not estimable
Karski 2005	3	147	4	165	0.2%	0.84 [0.19, 3.70]
Kaste 1979	0	32	0	32		Not estimable
Katsaros 1996	3	104	4	106	0.2%	0.76 [0.18, 3.33]
Kuitunen 2005	1	20	1	20	0.1%	1.00 [0.07, 14.90]
Later 2009	1	99	9	103	0.1%	0.12 [0.01, 0.90]
Lei 2020	0	150	0	50		Not estimable
Myles 2016	316	2311	350	2320	26.6%	0.91 [0.79, 1.04]
Reid 1997	0	20	0	21		Not estimable
Roberts 2020	86	5994	72	6015	5.4%	1.20 [0.88, 1.64]
Ross 2000	83	229	84	233	9.0%	1.01 [0.79, 1.28]
Shimizu 2011	1	81	0	79	0.1%	2.93 [0.12, 70.79]
Tengberg 2016	0	35	2	40	0.1%	0.23 [0.01, 4.59]
Tsementzis 1990	24	50	13	50	1.7%	1.85 [1.07, 3.20]
Vermeulen 1984	79	241	54	238	6.0%	1.44 [1.07, 1.94]
Volquind 2014	0	32	0	30		Not estimable
von Holstein 1987	1	72	2	82	0.1%	0.57 [0.05, 6.15]
Xu 2012	0	20	0	20		Not estimable
Yuan 2017	2	140	1	140	0.1%	2.00 [0.18, 21.81]
Zhang 2018	0	105	0	105		Not estimable
Zhang S 2020	0	64	2	32	0.1%	0.10 [0.01, 2.05]
**Subtotal (95% CI)**		**10941**		**10856**	**54.2%**	**1.18 [0.98, 1.43]**
Total events	671		642			
Heterogeneity: Tau² = 0.04; Chi² = 30.49, df = 20 (P = 0.06); I² = 34%						
Test for overall effect: Z = 1.79 (P = 0.07)						
**2.1.2 TXA dose ≤2g**						
Abdel-Aleem 2013	0	373	0	367		Not estimable
Aguilera 2013	1	41	0	42	0.1%	3.07 [0.13, 73.29]
Aguilera 2015	0	50	0	50		Not estimable
Ahmed 2015	0	62	0	62		Not estimable
Ahn 2012	1	38	0	38	0.1%	3.00 [0.13, 71.40]
Akgul 2016	0	35	1	35	0.1%	0.33 [0.01, 7.91]
Alimian 2011	0	42	0	42		Not estimable
Alizadeh Ghavidel 2015	8	100	6	100	0.5%	1.33 [0.48, 3.70]
Altun 2017	0	34	0	20		Not estimable
Alvarez 2008	0	46	0	49		Not estimable
Alvarez 2019	3	22	3	22	0.2%	1.00 [0.23, 4.42]
Apipan 2018	0	60	0	20		Not estimable
Auvinen 1987	0	39	0	37		Not estimable
Avvisati 1989	0	6	0	6		Not estimable
Barrachina 2016	2	71	2	37	0.1%	0.52 [0.08, 3.55]
Baruah 2016	0	30	0	30		Not estimable
Batibay 2018	0	35	0	35		Not estimable
Benoni 1996	4	43	4	43	0.3%	1.00 [0.27, 3.74]
Benoni 2000	3	20	3	20	0.2%	1.00 [0.23, 4.37]
Benoni 2001	1	18	1	20	0.1%	1.11 [0.07, 16.49]
Bidolegui 2014	0	25	0	25		Not estimable
Brown 1997	0	60	0	30		Not estimable
Caglar 2008	0	50	0	50		Not estimable
Cansancao 2018	0	10	0	10		Not estimable
Casati 2001	6	341	3	169	0.3%	0.99 [0.25, 3.91]
Casati 2002	2	30	3	30	0.2%	0.67 [0.12, 3.71]
Casati 2004	0	52	0	50		Not estimable
Chakravarthy 2012	0	25	0	25		Not estimable
Chakroun-Walha 2019	14	96	5	84	0.6%	2.45 [0.92, 6.52]
Chareancholvanich 2012	0	120	0	120		Not estimable
Charoencholvanich 2011	0	50	0	50		Not estimable
Chauhan 2003	0	96	0	24		Not estimable
Chen 2016	0	60	0	60		Not estimable
Chen 2019	14	88	12	88	1.0%	1.17 [0.57, 2.38]

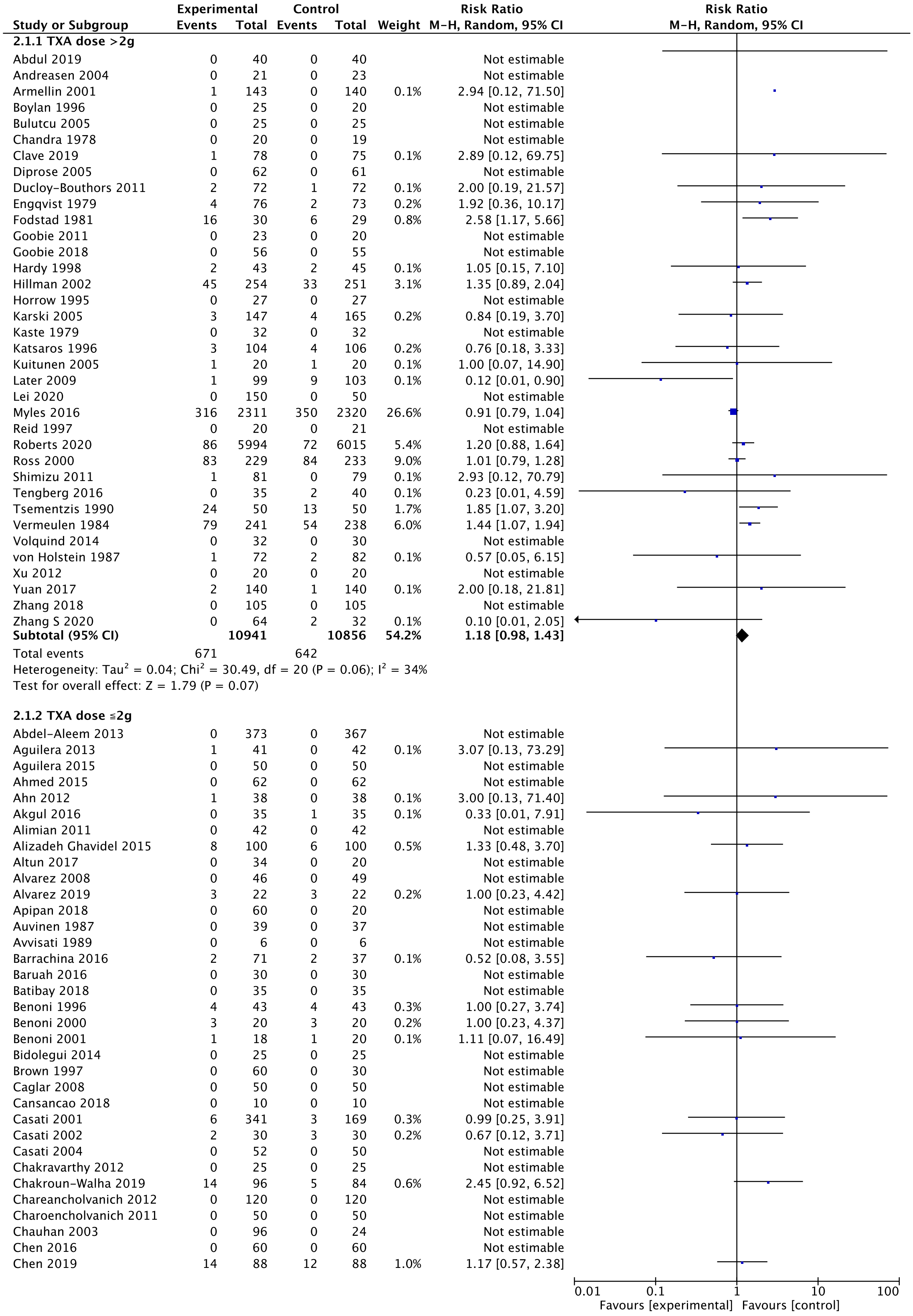

Chin 2020	1	42	1	39	0.1%	0.93 [0.06, 14.34]
Choi 2009	0	32	0	29		Not estimable
Christabel 2014	0	25	0	24		Not estimable
Claeys 2007	0	20	0	20		Not estimable
Clave 2019	0	76	0	75		Not estimable
Cohen 2021	0	27	0	17		Not estimable
Colomina 2016	2	44	1	51	0.1%	2.32 [0.22, 24.71]
Crescenti 2011	2	100	5	100	0.2%	0.40 [0.08, 2.01]
Cvetanovich 2018	0	52	1	56	0.1%	0.36 [0.01, 8.61]
Dadure 2011	0	19	0	20		Not estimable
Ebrahimi 2019	0	40	0	40		Not estimable
Ekback 2000	1	20	1	20	0.1%	1.00 [0.07, 14.90]
Eldaba 2013	0	50	0	50		Not estimable
Elwatidy 2008	0	32	0	32		Not estimable
Emara 2014	6	20	1	20	0.1%	6.00 [0.79, 45.42]
Engel 2001	0	12	0	12		Not estimable
Esfandiari 2012	7	75	6	75	0.5%	1.17 [0.41, 3.31]
Ezhevskaya 2018	0	80	0	80		Not estimable
Faraoni 2014	0	24	0	12		Not estimable
Farrokhi 2011	0	38	0	38		Not estimable
Felli 2019	0	40	0	40		Not estimable
Fenger-Eriksen 2019	0	15	0	15		Not estimable
Fraval 2017	0	50	0	51		Not estimable
Gai 2004	0	91	0	89		Not estimable
Garg 2012	0	26	0	26		Not estimable
Garneti 2004	1	25	0	25	0.1%	3.00 [0.13, 70.30]
Good 2003	2	27	2	24	0.1%	0.89 [0.14, 5.83]
Goswami 2013	0	60	0	30		Not estimable
Greiff 2012	3	30	4	33	0.3%	0.82 [0.20, 3.39]
Gungorduk 2011	0	330	0	330		Not estimable
Gungorduk 2013	0	220	0	219		Not estimable
Gupta 2012	0	30	0	30		Not estimable
Helito 2019	0	30	1	30	0.1%	0.33 [0.01, 7.87]
Hiippala 1995	1	15	2	13	0.1%	0.43 [0.04, 4.25]
Hiippala 1997	2	39	3	38	0.2%	0.65 [0.11, 3.67]
Horrow 1991	2	37	1	44	0.1%	2.38 [0.22, 25.20]
Horrow 1995	0	43	0	27		Not estimable
Hsu 2015	0	30	0	30		Not estimable
Husted 2003	0	20	0	20		Not estimable
Jansen 1999	0	21	1	21	0.1%	0.33 [0.01, 7.74]
Jares 2003	1	22	1	25	0.1%	1.14 [0.08, 17.11]
Jaszczyk 2015	1	61	0	63	0.1%	3.10 [0.13, 74.58]
Johansson 2005	0	47	0	53		Not estimable
Jokar 2017	0	40	0	40		Not estimable
Kakar 2009	0	25	0	25		Not estimable
Karaaslan 2015	0	53	0	52		Not estimable
Karski 1995	3	99	0	48	0.1%	3.43 [0.18, 65.10]
Kazemi 2010	0	32	1	32	0.1%	0.33 [0.01, 7.89]
Keyhani 2016	0	40	0	40		Not estimable
Kim 2014	0	90	0	90		Not estimable
Kim 2017	0	48	2	24	0.1%	0.10 [0.01, 2.05]
Kimura 2019	0	128	0	128		Not estimable
Kulkarni 2016	0	120	0	120		Not estimable
Kundu 2015	3	30	2	30	0.2%	1.50 [0.27, 8.34]
Lack 2017	1	42	0	46	0.1%	3.28 [0.14, 78.36]
Lee 2012	3	36	4	36	0.3%	0.75 [0.18, 3.11]
Lee 2013	0	34	0	34		Not estimable
Lemay 2004	0	20	0	19		Not estimable
Lin 2012	1	101	0	50	0.1%	1.50 [0.06, 36.18]
Liu 2018	23	150	15	74	1.5%	0.76 [0.42, 1.36]
Liu 2020	0	37	0	35		Not estimable
Lundin 2014	2	50	5	50	0.2%	0.40 [0.08, 1.97]
Luo 2019	1	44	4	46	0.1%	0.26 [0.03, 2.25]
Ma 2019	1	62	0	62	0.1%	3.00 [0.12, 72.25]
MacGillivray 2011	2	40	0	20	0.1%	2.56 [0.13, 50.95]
Maddali 2007	0	111	0	111		Not estimable
Maged 2015	0	100	0	100		Not estimable
Mahmood 2017	0	100	0	73		Not estimable
Maniar 2012	0	160	0	40		Not estimable
Mansouri 2012	0	30	0	30		Not estimable
Mehr-Aein 2007	0	33	0	33		Not estimable
Meng 2019	0	30	0	30		Not estimable
Misfeld 1998	0	14	0	14		Not estimable
Molloy 2005	0	50	0	50		Not estimable
Monaco 2020	2	50	2	50	0.1%	1.00 [0.15, 6.82]
Motififard 2015	0	45	0	45		Not estimable
Mu 2019	0	45	0	42		Not estimable
Na 2016	0	29	0	26		Not estimable
Nagabhushan 2018	0	25	0	25		Not estimable
Neilipovitz 2001	0	22	0	18		Not estimable
Nejad 2011	1	50	0	50	0.1%	3.00 [0.13, 71.92]
Niskanen 2005	0	19	0	20		Not estimable
Nugent 2019	0	18	0	23		Not estimable
Oremus 2014	0	49	2	49	0.1%	0.20 [0.01, 4.06]
Orpen 2006	0	15	0	14		Not estimable

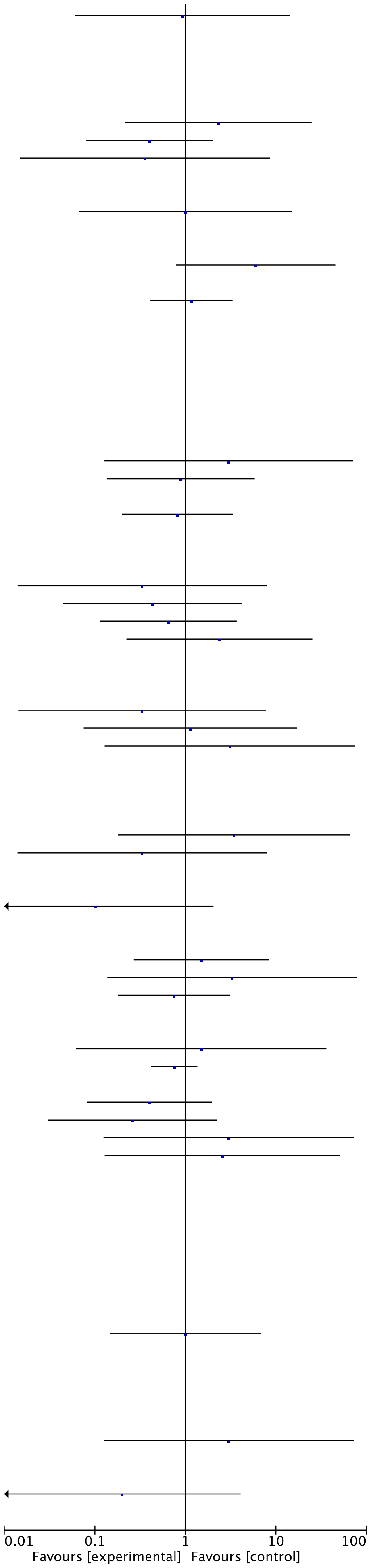

Painter 2018	2	71	4	69	0.2%	0.49 [0.09, 2.57]
Palija 2020	0	40	0	40		Not estimable
Peters 2015	1	19	0	13	0.1%	2.10 [0.09, 47.89]
Pleym 2003	0	40	1	40	0.1%	0.33 [0.01, 7.95]
Prakash 2017	0	50	1	50	0.1%	0.33 [0.01, 7.99]
Prasad 2018	0	40	0	20		Not estimable
Raksakietisak 2015	0	39	0	39		Not estimable
Ramezani 2005	0	32	0	30		Not estimable
Ray 2016	0	50	0	50		Not estimable
Roberts 2019	101	6406	102	6331	7.1%	0.98 [0.74, 1.29]
Rowell 2020	44	657	30	309	2.7%	0.69 [0.44, 1.08]
Sahu 2019	0	50	0	50		Not estimable
Sallam 2019	0	43	0	43		Not estimable
Sankar 2012	0	25	0	25		Not estimable
Santos 2006	2	29	2	31	0.1%	1.07 [0.16, 7.10]
Seddighi 2019	0	20	0	20		Not estimable
Sentilhes 2018	1	1945	4	1946	0.1%	0.25 [0.03, 2.24]
Senturk 2013	0	101	0	122		Not estimable
Seo 2013	0	50	2	50	0.1%	0.20 [0.01, 4.06]
Seol 2016	0	50	0	50		Not estimable
Seviciu 2016	0	29	0	32		Not estimable
Shaaban 2016	0	66	0	66		Not estimable
Shahid 2013	0	38	0	36		Not estimable
Shakur 2010	168	10093	201	10114	12.8%	0.84 [0.68, 1.03]
Shakur 2017	30	10051	34	10009	2.2%	0.88 [0.54, 1.43]
Shen 2015	4	46	4	46	0.3%	1.00 [0.27, 3.76]
Shi 2013	5	274	7	278	0.4%	0.72 [0.23, 2.26]
Shi 2017	0	50	0	46		Not estimable
Shi, J 2013	3	58	3	59	0.2%	1.02 [0.21, 4.83]
Shore-Lesserson 1996	5	17	3	13	0.3%	1.27 [0.37, 4.39]
Spitler 2019	2	47	1	46	0.1%	1.96 [0.18, 20.85]
Sprigg 2014	1	16	0	8	0.1%	1.59 [0.07, 35.15]
Sprigg 2018	66	1161	54	1164	4.3%	1.23 [0.86, 1.74]
Stowers 2017	1	51	0	23	0.1%	1.38 [0.06, 32.76]
Sun 2017	4	135	0	45	0.1%	3.04 [0.17, 55.47]
Taghaddomi 2009	0	50	0	50		Not estimable
Thipparampall 2020	0	39	0	20		Not estimable
Tian 2018	0	50	0	50		Not estimable
Topsoee 2016	0	165	0	167		Not estimable
Tsutsumimoto 2011	0	35	2	40	0.1%	0.23 [0.01, 4.59]
Vanek 2005	0	39	0	20		Not estimable
Vara 2017	3	50	2	50	0.2%	1.50 [0.26, 8.60]
Veien 2002	0	15	0	15		Not estimable
Vel 2015	0	50	0	50		Not estimable
Vela 2012	3	22	3	22	0.2%	1.00 [0.23, 4.42]
Verma 2014	0	36	0	47		Not estimable
Vijay 2013	0	45	0	45		Not estimable
Wang 2012	0	116	0	115		Not estimable
Wang 2013	0	30	0	30		Not estimable
Wang 2016	1	81	0	38	0.1%	1.43 [0.06, 34.24]
Wang 2018	15	39	16	41	1.7%	0.99 [0.57, 1.71]
Wang, J 2016	10	100	10	98	0.8%	0.98 [0.43, 2.25]
Watts 2017	5	69	6	69	0.4%	0.83 [0.27, 2.60]
Wei 2006	0	36	0	40		Not estimable
Wong 2008	1	73	1	73	0.1%	1.00 [0.06, 15.69]
Wu 2006	0	108	0	106		Not estimable
Xie 2015	2	45	1	45	0.1%	2.00 [0.19, 21.28]
Xu 2013	2	88	2	86	0.1%	0.98 [0.14, 6.78]
Xu 2019	1	68	0	67	0.1%	2.96 [0.12, 71.31]
Yamasaki 2004	0	20	0	20		Not estimable
Yamasaki 2005	0	21	0	21		Not estimable
Ye 2019	20	160	2	40	0.3%	2.50 [0.61, 10.26]
Yen 2017	0	33	0	32		Not estimable
Yi 2016	2	50	1	50	0.1%	2.00 [0.19, 21.36]
Yutthakasemsunt 2013	0	120	3	118	0.1%	0.14 [0.01, 2.69]
Zabeeda 2002	0	25	0	25		Not estimable
Zaman 2019	0	88	0	88		Not estimable
Zekcer 2016	0	30	4	30	0.1%	0.11 [0.01, 1.98]
Zhang 2016	1	25	2	25	0.1%	0.50 [0.05, 5.17]
Zhang 2020	3	61	1	61	0.1%	3.00 [0.32, 28.04]
Zhang 2021	6	106	2	59	0.2%	1.67 [0.35, 8.01]
Zhang S 2020	0	32	2	32	0.1%	0.20 [0.01, 4.01]
Zhao 2018	0	40	0	40		Not estimable
Zhou 2018	0	58	0	58		Not estimable
Zhou 2019	3	50	7	50	0.3%	0.43 [0.12, 1.56]
Zhu 2020	11	100	4	50	0.4%	1.38 [0.46, 4.10]
Zohar 2004	0	40	0	20		Not estimable
Zufferey 2010	8	57	3	53	0.3%	2.48 [0.69, 8.86]
**Subtotal (95% CI)**		**41175**		**39489**	**45.8%**	**0.94 [0.84, 1.05]**
Total events	671		638			

Heterogeneity: $Tau^2 = 0.00$; $Chi^2 = 51.10$, df = 86 (P = 1.00); $I^2 = 0\%$

Test for overall effect: Z = 1.15 (P = 0.25)

**Total (95% CI)**		**52116**		**50345**	**100.0%**	**1.00 [0.93, 1.08]**
Total events	1342		1280			

Heterogeneity: $Tau^2 = 0.00$; $Chi^2 = 84.32$, df = 107 (P = 0.95); $I^2 = 0\%$

Test for overall effect: Z = 0.09 (P = 0.93)

Test for subgroup differences: $Chi^2 = 4.50$, df = 1 (P = 0.03), $I^2 = 77.8\%$

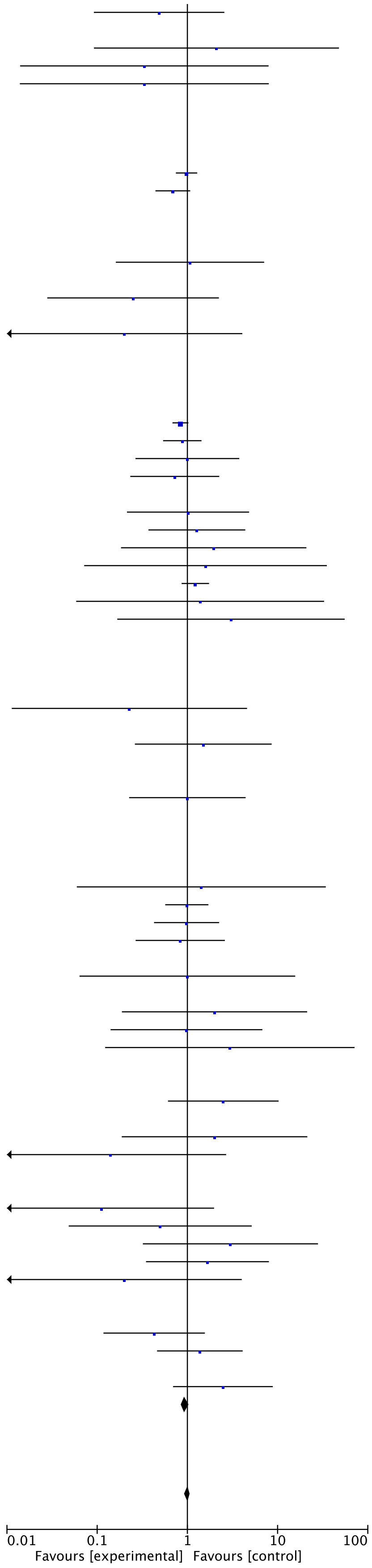

Figure S7. Forest plot of the thrombotic events: subgroup analysis in children.

Study or Subgroup	Experimental Events	Experimental Total	Control Events	Control Total	Weight	Risk Ratio M-H, Random, 95% CI
**18.1.1 Children**						
Bulutcu 2005	0	25	0	25		Not estimable
Chauhan 2003	0	96	0	24		Not estimable
Dadure 2011	0	19	0	20		Not estimable
Eldaba 2013	0	50	0	50		Not estimable
Goobie 2011	0	23	0	20		Not estimable
Goobie 2018	0	56	0	55		Not estimable
Reid 1997	0	20	0	21		Not estimable
Shimizu 2011	1	81	0	79	0.1%	2.93 [0.12, 70.79]
**Subtotal (95% CI)**		**370**		**294**	**0.1%**	**2.93 [0.12, 70.79]**
Total events	1		0			

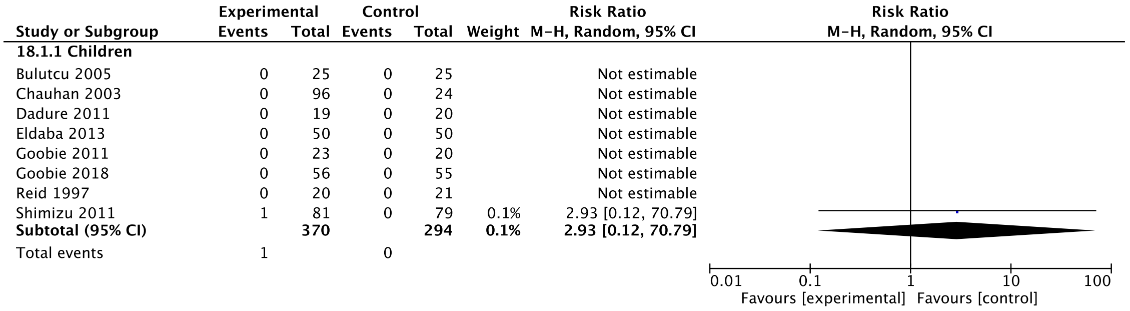

Figure S8. Forest plot of the seizures: subgroup analysis by underlying disease

Study or Subgroup	Experimental Events	Experimental Total	Control Events	Control Total	Weight	Risk Ratio M-H, Random, 95% CI
**5.1.2 Trauma**						
Chakroun-Walha 2019	4	96	1	84	1.4%	3.50 [0.40, 30.70]
Roberts 2019	206	6406	186	6311	28.3%	1.09 [0.90, 1.33]
Rowell 2020	22	657	7	309	7.4%	1.48 [0.64, 3.42]
**Subtotal (95% CI)**		**7159**		**6704**	**37.1%**	**1.12 [0.93, 1.35]**
Total events	232		194			
Heterogeneity: $Tau^2 = 0.00$; $Chi^2 = 1.55$, df = 2 (P = 0.46); $I^2 = 0\%$						
Test for overall effect: Z = 1.15 (P = 0.25)						
**5.1.3 Obstetric and gynecology**						
Ducloy-Bouthors 2011	0	72	0	72		Not estimable
Sentilhes 2018	0	1945	1	1946	0.6%	0.33 [0.01, 8.18]
Shakur 2017	33	10051	43	10009	16.6%	0.76 [0.49, 1.20]
**Subtotal (95% CI)**		**12068**		**12027**	**17.2%**	**0.75 [0.48, 1.18]**
Total events	33		44			
Heterogeneity: $Tau^2 = 0.00$; $Chi^2 = 0.25$, df = 1 (P = 0.61); $I^2 = 0\%$						
Test for overall effect: Z = 1.25 (P = 0.21)						
**5.1.4 Cardiac surgery**						
Chakravarthy 2012	0	25	0	25		Not estimable
Chauhan 2004	0	50	0	50		Not estimable
Esfandiari 2012	0	75	0	75		Not estimable
Faraoni 2014	0	24	0	12		Not estimable
Greiff 2012	0	30	0	33		Not estimable
Maddali 2007	0	111	0	111		Not estimable
Mansouri 2012	0	30	0	30		Not estimable
Mehr-Aein 2007	0	33	0	33		Not estimable
Monaco 2020	0	50	0	50		Not estimable
Myles 2016	15	2311	2	2320	2.8%	7.53 [1.72, 32.89]
Shi 2013	4	274	2	278	2.2%	2.03 [0.37, 10.99]
Shi, J 2013	1	58	0	59	0.7%	3.05 [0.13, 73.39]
Shimizu 2011	0	81	0	79		Not estimable
Yanartas 2015	0	34	0	32		Not estimable
Zhang 2018	2	105	0	105	0.7%	5.00 [0.24, 102.91]
**Subtotal (95% CI)**		**3291**		**3292**	**6.4%**	**4.20 [1.56, 11.32]**
Total events	22		4			
Heterogeneity: $Tau^2 = 0.00$; $Chi^2 = 1.40$, df = 3 (P = 0.70); $I^2 = 0\%$						
Test for overall effect: Z = 2.84 (P = 0.004)						
**5.1.5 Orthopaedic surgery**						
Alvarez 2019	0	22	0	22		Not estimable
Benoni 2000	0	20	0	20		Not estimable
Carabini 2017	0	31	0	30		Not estimable
Colomina 2016	0	44	0	51		Not estimable
Goobie 2018	0	23	0	20		Not estimable
Ma 2019	0	62	0	62		Not estimable
Oremus 2014	0	49	0	49		Not estimable
Xie 2015	0	45	0	45		Not estimable
Zufferey 2010	1	57	0	53	0.7%	2.79 [0.12, 67.10]
**Subtotal (95% CI)**		**353**		**352**	**0.7%**	**2.79 [0.12, 67.10]**
Total events	1		0			
Heterogeneity: Not applicable						
Test for overall effect: Z = 0.63 (P = 0.53)						
**5.1.6 Intracranial surgery**						
Sprigg 2018	77	1161	85	1164	23.3%	0.91 [0.67, 1.22]
**Subtotal (95% CI)**		**1161**		**1164**	**23.3%**	**0.91 [0.67, 1.22]**
Total events	77		85			
Heterogeneity: Not applicable						
Test for overall effect: Z = 0.63 (P = 0.53)						
**5.1.7 Gastrointestinal surgery**						
Roberts 2020	38	5994	22	6015	14.2%	1.73 [1.03, 2.93]
**Subtotal (95% CI)**		**5994**		**6015**	**14.2%**	**1.73 [1.03, 2.93]**
Total events	38		22			
Heterogeneity: Not applicable						
Test for overall effect: Z = 2.06 (P = 0.04)						
**5.1.8 Others**						
Cohen 2021	0	27	0	17		Not estimable
Dadure 2011	0	19	0	20		Not estimable
Eldaba 2013	0	50	0	50		Not estimable
Fenger-Eriksen 2019	0	15	0	15		Not estimable
Goobie 2011	0	56	0	55		Not estimable
Hooda 2015	1	30	2	30	1.2%	0.50 [0.05, 5.22]
Prasad 2018	0	40	0	20		Not estimable
Xu 2013	0	88	0	86		Not estimable
**Subtotal (95% CI)**		**325**		**293**	**1.2%**	**0.50 [0.05, 5.22]**
Total events	1		2			

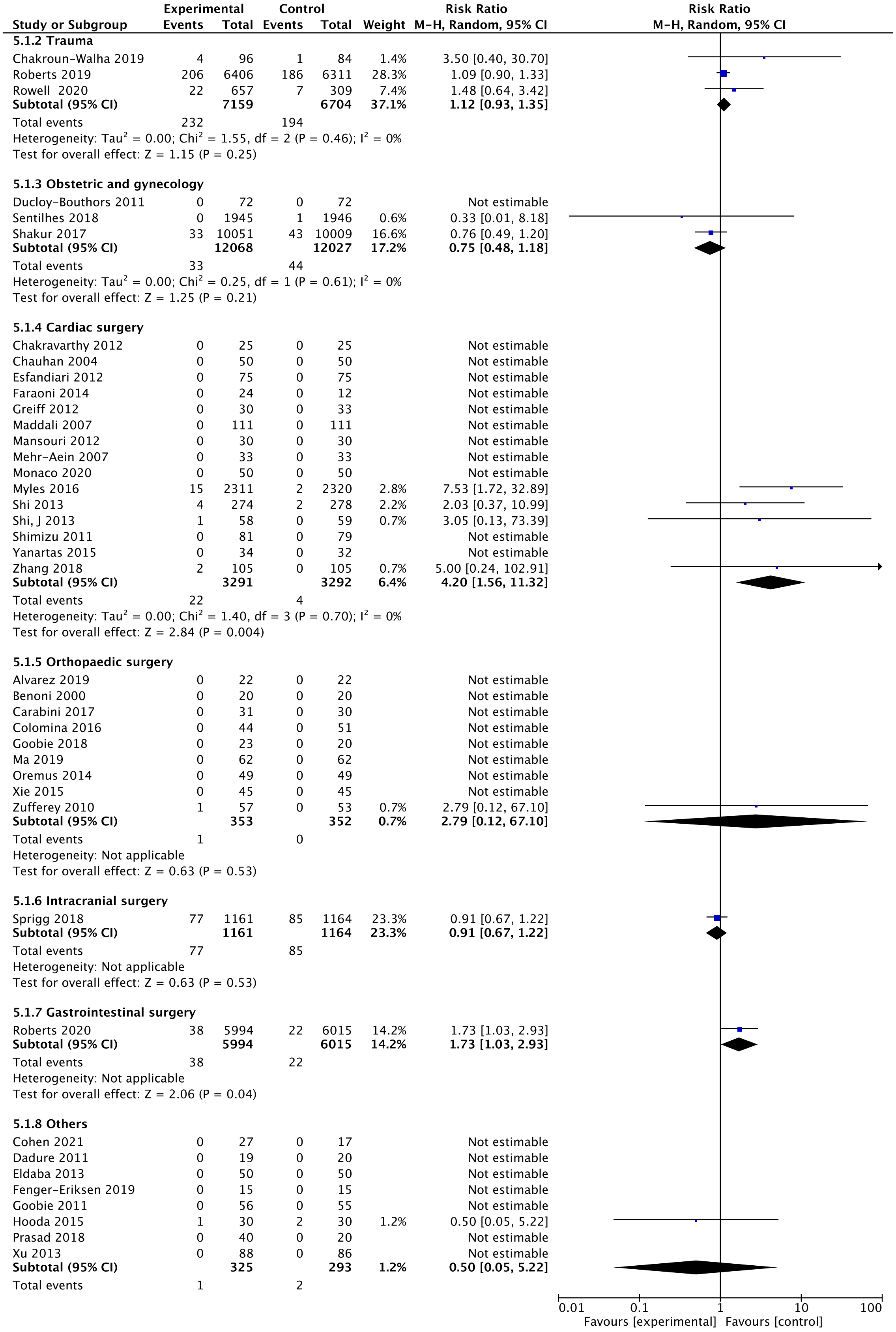

Heterogeneity: Not applicable
Test for overall effect: Z = 0.58 (P = 0.56)

Total (95% CI) 30351 29847 100.0% 1.18 [0.91, 1.53]
Total events 404 351
Heterogeneity: $Tau^2 = 0.05$; $Chi^2 = 18.31$, df = 12 (P = 0.11); $I^2 = 34\%$
Test for overall effect: Z = 1.24 (P = 0.21)
Test for subgroup differences: $Chi^2 = 15.05$, df = 6 (P = 0.02), $I^2 = 60.1\%$

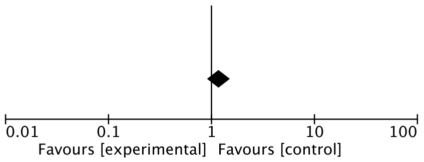

Figure S9. Forest plot of the seizures: subgroup analysis by TXA (tranexamic acid) dose.

Study or Subgroup	Experimental Events	Experimental Total	Control Events	Control Total	Weight	Risk Ratio M-H, Random, 95% CI
**6.1.1 TXA dose >2g**						
Ducloy-Bouthors 2011	0	72	0	72		Not estimable
Goobie 2011	0	56	0	55		Not estimable
Goobie 2018	0	23	0	20		Not estimable
Myles 2016	15	2311	2	2320	2.8%	7.53 [1.72, 32.89]
Roberts 2020	38	5994	22	6015	14.2%	1.73 [1.03, 2.93]
Shimizu 2011	0	81	0	79		Not estimable
Zhang 2018	2	105	0	105	0.7%	5.00 [0.24, 102.91]
**Subtotal (95% CI)**		**8642**		**8666**	**17.7%**	**3.05 [1.01, 9.20]**
Total events	55		24			
Heterogeneity: Tau^2 = 0.47; Chi^2 = 3.82, df = 2 (P = 0.15); I^2 = 48%						
Test for overall effect: Z = 1.98 (P = 0.05)						
**6.1.2 TXA dose ≤2g**						
Alvarez 2019	0	22	0	22		Not estimable
Benoni 2000	0	20	0	20		Not estimable
Carabini 2017	0	31	0	30		Not estimable
Chakravarthy 2012	0	25	0	25		Not estimable
Chakroun-Walha 2019	4	96	1	84	1.4%	3.50 [0.40, 30.70]
Chauhan 2004	0	50	0	50		Not estimable
Cohen 2021	0	27	0	17		Not estimable
Colomina 2016	0	44	0	51		Not estimable
Dadure 2011	0	19	0	20		Not estimable
Eldaba 2013	0	50	0	50		Not estimable
Esfandiari 2012	0	75	0	75		Not estimable
Faraoni 2014	0	24	0	12		Not estimable
Fenger-Eriksen 2019	0	15	0	15		Not estimable
Greiff 2012	0	30	0	33		Not estimable
Hooda 2015	1	30	2	30	1.2%	0.50 [0.05, 5.22]
Ma 2019	0	62	0	62		Not estimable
Maddali 2007	0	111	0	111		Not estimable
Mansouri 2012	0	30	0	30		Not estimable
Mehr-Aein 2007	0	33	0	33		Not estimable
Monaco 2020	0	50	0	50		Not estimable
Oremus 2014	0	49	0	49		Not estimable
Prasad 2018	0	40	0	20		Not estimable
Roberts 2019	206	6406	186	6311	28.3%	1.09 [0.90, 1.33]
Rowell 2020	22	657	7	309	7.4%	1.48 [0.64, 3.42]
Sentilhes 2018	0	1945	1	1946	0.6%	0.33 [0.01, 8.18]
Shakur 2017	33	10051	43	10009	16.6%	0.76 [0.49, 1.20]
Shi 2013	4	274	2	278	2.2%	2.03 [0.37, 10.99]
Shi, J 2013	1	58	0	59	0.7%	3.05 [0.13, 73.39]
Sprigg 2018	77	1161	85	1164	23.3%	0.91 [0.67, 1.22]
Xie 2015	0	45	0	45		Not estimable
Xu 2013	0	88	0	86		Not estimable
Yanartas 2015	0	34	0	32		Not estimable
Zufferey 2010	1	57	0	53	0.7%	2.79 [0.12, 67.10]
**Subtotal (95% CI)**		**21709**		**21181**	**82.3%**	**1.02 [0.88, 1.19]**
Total events	349		327			
Heterogeneity: Tau^2 = 0.00; Chi^2 = 6.90, df = 9 (P = 0.65); I^2 = 0%						
Test for overall effect: Z = 0.27 (P = 0.78)						
**Total (95% CI)**		**30351**		**29847**	**100.0%**	**1.18 [0.91, 1.53]**
Total events	404		351			
Heterogeneity: Tau^2 = 0.05; Chi^2 = 18.31, df = 12 (P = 0.11); I^2 = 34%						
Test for overall effect: Z = 1.24 (P = 0.21)						
Test for subgroup differences: Chi^2 = 3.72, df = 1 (P = 0.05), I^2 = 73.1%						

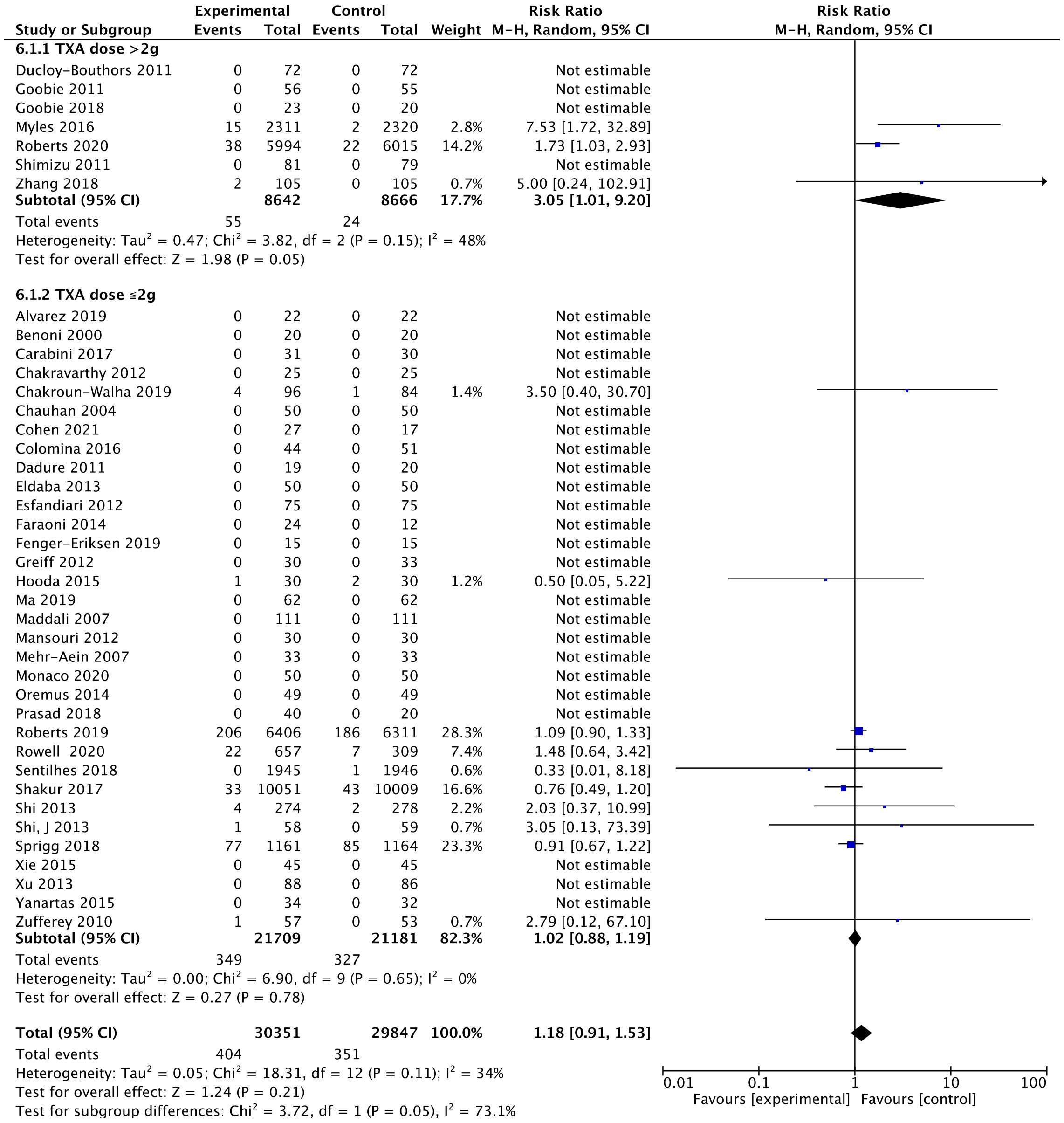

Figure S10. Forest plot of the seizures: subgroup analysis in children.

Study or Subgroup	Experimental Events	Experimental Total	Control Events	Control Total	Weight	Risk Ratio M-H, Random, 95% CI	Risk Ratio M-H, Random, 95% CI
**19.1.1 Children**							
Chauhan 2004	0	50	0	50		Not estimable	
Dadure 2011	0	19	0	20		Not estimable	
Eldaba 2013	0	50	0	50		Not estimable	
Goobie 2011	0	56	0	55		Not estimable	
Goobie 2018	0	23	0	20		Not estimable	
**Subtotal (95% CI)**		**198**		**195**		**Not estimable**	
Total events	0		0				

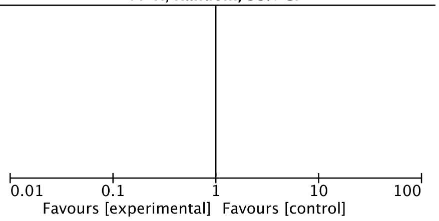

Figure S11. Forest plot of the venous thromboembolism: subgroup analysis by underlying disease.

Study or Subgroup	Experimental Events	Experimental Total	Control Events	Control Total	Weight	Risk Ratio M-H, Random, 95% CI
**12.1.1 Trauma**						
Chakroun-Walha 2019	14	96	5	84	1.6%	2.45 [0.92, 6.52]
Ebrahimi 2019	0	40	0	40		Not estimable
Jokar 2017	0	40	0	40		Not estimable
Roberts 2019	43	6406	48	6331	9.1%	0.89 [0.59, 1.33]
Rowell 2020	22	657	14	309	3.6%	0.74 [0.38, 1.42]
Shakur 2010	112	10093	112	10114	22.7%	1.00 [0.77, 1.30]
Yutthakasemsunt 2013	0	120	0	118		Not estimable
**Subtotal (95% CI)**		**17452**		**17036**	**37.0%**	**0.99 [0.74, 1.31]**
Total events	191		179			
Heterogeneity: $Tau^2 = 0.03$; $Chi^2 = 4.35$, df = 3 (P = 0.23); $I^2 = 31\%$						
Test for overall effect: Z = 0.09 (P = 0.93)						
**12.1.2 Obstetric and gynecology**						
Abdel-Aleem 2013	0	373	0	367		Not estimable
Abdul 2019	0	40	0	40		Not estimable
Ahmed 2015	0	62	0	62		Not estimable
Caglar 2008	0	50	0	50		Not estimable
Ducloy-Bouthors 2011	2	72	1	72	0.3%	2.00 [0.19, 21.57]
Gai 2004	0	91	0	89		Not estimable
Goswami 2013	0	60	0	30		Not estimable
Gungorduk 2011	0	330	0	330		Not estimable
Gungorduk 2013	0	220	0	219		Not estimable
Gupta 2012	0	30	0	30		Not estimable
Lundin 2014	2	50	4	50	0.6%	0.50 [0.10, 2.61]
Maged 2015	0	100	0	100		Not estimable
Ray 2016	0	50	0	50		Not estimable
Sahu 2019	0	50	0	50		Not estimable
Sallam 2019	0	43	0	43		Not estimable
Sentilhes 2018	0	1945	1	1946	0.2%	0.33 [0.01, 8.18]
Senturk 2013	0	101	0	122		Not estimable
Shaaban 2016	0	66	0	66		Not estimable
Shahid 2013	0	38	0	36		Not estimable
Shakur 2017	20	10051	25	10009	4.5%	0.80 [0.44, 1.43]
Topsoee 2016	0	165	0	167		Not estimable
Xu 2013	2	88	2	86	0.4%	0.98 [0.14, 6.78]
**Subtotal (95% CI)**		**14075**		**14014**	**5.9%**	**0.79 [0.47, 1.32]**
Total events	26		33			
Heterogeneity: $Tau^2 = 0.00$; $Chi^2 = 1.21$, df = 4 (P = 0.88); $I^2 = 0\%$						
Test for overall effect: Z = 0.91 (P = 0.36)						
**12.1.3 Cardiac surgery**						
Ahn 2012	0	38	0	38		Not estimable
Alizadeh Ghavidel 2015	0	100	0	100		Not estimable
Altun 2017	0	34	0	20		Not estimable
Armellin 2001	0	143	0	140		Not estimable
Bulutcu 2005	0	25	0	25		Not estimable
Casati 2002	0	30	0	30		Not estimable
Casati 2004	0	52	0	50		Not estimable
Faraoni 2014	0	24	0	12		Not estimable
Horrow 1991	0	37	1	44	0.2%	0.39 [0.02, 9.41]
Horrow 1995	0	121	0	27		Not estimable
Jares 2003	0	22	0	25		Not estimable
Katsaros 1996	0	104	2	106	0.2%	0.20 [0.01, 4.19]
Mansouri 2012	0	30	0	30		Not estimable
Mehr-Aein 2007	0	33	0	33		Not estimable
Misfeld 1998	0	14	0	14		Not estimable
Monaco 2020	0	50	0	50		Not estimable
Myles 2016	15	2311	15	2320	3.0%	1.00 [0.49, 2.05]
Pleym 2003	0	40	1	40	0.2%	0.33 [0.01, 7.95]
Reid 1997	0	20	0	20		Not estimable
Shore-Lesserson 1996	2	17	0	13	0.2%	3.89 [0.20, 74.67]
Taghaddomi 2009	0	50	0	50		Not estimable
Vanek 2005	0	32	0	30		Not estimable
Wang 2012	0	116	0	115		Not estimable
Wei 2006	0	36	0	40		Not estimable
Zhang 2018	0	105	0	105		Not estimable
**Subtotal (95% CI)**		**3584**		**3477**	**3.7%**	**0.91 [0.48, 1.75]**
Total events	17		19			
Heterogeneity: $Tau^2 = 0.00$; $Chi^2 = 2.60$, df = 4 (P = 0.63); $I^2 = 0\%$						
Test for overall effect: Z = 0.27 (P = 0.79)						
**12.1.4 Orthopaedic surgery**						
Aguilera 2013	0	41	0	42		Not estimable
Aguilera 2015	0	50	0	50		Not estimable
Akgul 2016	0	35	1	35	0.2%	0.33 [0.01, 7.91]
Alvarez 2008	0	46	0	49		Not estimable

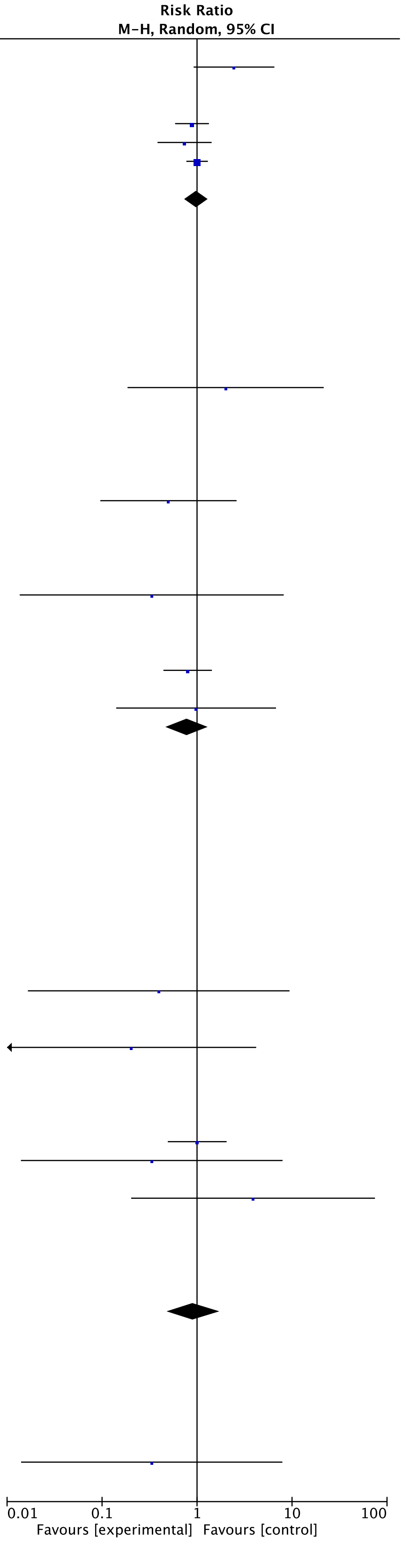

Alvarez 2019	3	22	3	22	0.7%	1.00 [0.23, 4.42]
Barrachina 2016	0	71	0	37		Not estimable
Baruah 2016	0	30	0	30		Not estimable
Batibay 2018	0	35	0	35		Not estimable
Benoni 1996	4	43	4	43	0.9%	1.00 [0.27, 3.74]
Benoni 2000	3	20	3	20	0.7%	1.00 [0.23, 4.37]
Benoni 2001	1	18	1	20	0.2%	1.11 [0.07, 16.49]
Bidolegui 2014	0	25	0	25		Not estimable
Chareancholvanich 2012	0	50	0	50		Not estimable
Charoencholvanich 2011	0	120	0	120		Not estimable
Chen 2016	0	60	0	60		Not estimable
Chen 2019	12	88	12	88	2.8%	1.00 [0.48, 2.10]
Chin 2020	1	42	0	39	0.2%	2.79 [0.12, 66.54]
Claeys 2007	0	20	0	20		Not estimable
Colomina 2016	2	44	1	51	0.3%	2.32 [0.22, 24.71]
Cvetanovich 2018	0	52	1	56	0.2%	0.36 [0.01, 8.61]
Ekback 2000	1	20	1	20	0.2%	1.00 [0.07, 14.90]
Elwatidy 2008	0	32	0	32		Not estimable
Emara 2014	5	20	1	20	0.4%	5.00 [0.64, 39.06]
Engel 2001	3	12	0	12	0.2%	7.00 [0.40, 122.44]
Ezhevskaya 2018	0	80	0	80		Not estimable
Farrokhi 2011	0	38	0	38		Not estimable
Felli 2019	0	40	0	40		Not estimable
Fraval 2017	0	50	0	51		Not estimable
Garg 2012	0	26	0	26		Not estimable
Garneti 2004	1	25	0	25	0.2%	3.00 [0.13, 70.30]
Goobie 2018	0	56	0	55		Not estimable
Good 2003	2	27	2	24	0.4%	0.89 [0.14, 5.83]
Helito 2019	0	30	1	30	0.2%	0.33 [0.01, 7.87]
Hiippala 1995	0	15	2	13	0.2%	0.17 [0.01, 3.34]
Hiippala 1997	2	39	3	38	0.5%	0.65 [0.11, 3.67]
Hsu 2015	0	30	0	30		Not estimable
Husted 2003	0	20	0	20		Not estimable
Jansen 1999	0	21	0	21		Not estimable
Jaszczyk 2015	1	61	0	63	0.2%	3.10 [0.13, 74.58]
Johansson 2005	0	47	0	53		Not estimable
Kakar 2009	0	25	0	25		Not estimable
Karaaslan 2015	0	53	0	52		Not estimable
Kazemi 2010	0	32	1	32	0.2%	0.33 [0.01, 7.89]
Keyhani 2016	0	40	0	40		Not estimable
Kim 2014	0	90	0	90		Not estimable
Kim 2017	0	48	2	24	0.2%	0.10 [0.01, 2.05]
Kimura 2019	0	128	0	128		Not estimable
Kundu 2015	3	30	2	30	0.5%	1.50 [0.27, 8.34]
Lack 2017	1	42	0	46	0.2%	3.28 [0.14, 78.36]
Lee 2012	3	36	4	36	0.8%	0.75 [0.18, 3.11]
Lee 2013	0	34	0	34		Not estimable
Lei 2020	0	150	0	50		Not estimable
Lemay 2004	0	20	0	19		Not estimable
Lin 2012	1	101	0	50	0.2%	1.50 [0.06, 36.18]
Liu 2018	23	150	15	74	4.4%	0.76 [0.42, 1.36]
Liu 2020	0	37	0	35		Not estimable
Luo 2019	0	44	1	46	0.2%	0.35 [0.01, 8.33]
Ma 2019	1	62	0	62	0.2%	3.00 [0.12, 72.25]
MacGillivray 2011	2	40	0	20	0.2%	2.56 [0.13, 50.95]
Mahmood 2017	0	100	0	73		Not estimable
Maniar 2012	0	160	0	40		Not estimable
Molloy 2005	0	50	0	50		Not estimable
Motififard 2015	0	45	0	45		Not estimable
Mu 2019	0	45	0	42		Not estimable
Na 2016	0	29	0	26		Not estimable
Nagabhushan 2018	0	25	0	25		Not estimable
Neilipovitz 2001	0	22	0	18		Not estimable
Niskanen 2005	0	19	0	20		Not estimable
Nugent 2019	0	18	0	23		Not estimable
Oremus 2014	0	49	2	48	0.2%	0.20 [0.01, 3.98]
Orpen 2006	0	15	0	14		Not estimable
Painter 2018	1	71	2	69	0.3%	0.49 [0.05, 5.24]
Palija 2020	0	40	0	40		Not estimable
Peters 2015	1	19	0	13	0.2%	2.10 [0.09, 47.89]
Prakash 2017	0	50	1	50	0.2%	0.33 [0.01, 7.99]
Raksakietisak 2015	0	39	0	39		Not estimable
Sankar 2012	0	25	0	25		Not estimable
Seddighi 2019	0	20	0	20		Not estimable
Seo 2013	0	50	2	50	0.2%	0.20 [0.01, 4.06]
Seol 2016	0	50	0	50		Not estimable
Seviciu 2016	0	29	0	32		Not estimable
Shen 2015	4	46	4	46	0.9%	1.00 [0.27, 3.76]
Shi 2017	0	50	0	46		Not estimable
Stowers 2017	1	51	0	23	0.2%	1.38 [0.06, 32.76]
Sun 2017	4	135	0	45	0.2%	3.04 [0.17, 55.47]
Tengberg 2016	0	35	0	40		Not estimable
Thipparampall 2020	0	39	0	20		Not estimable
Tian 2018	2	50	2	50	0.4%	1.00 [0.15, 6.82]

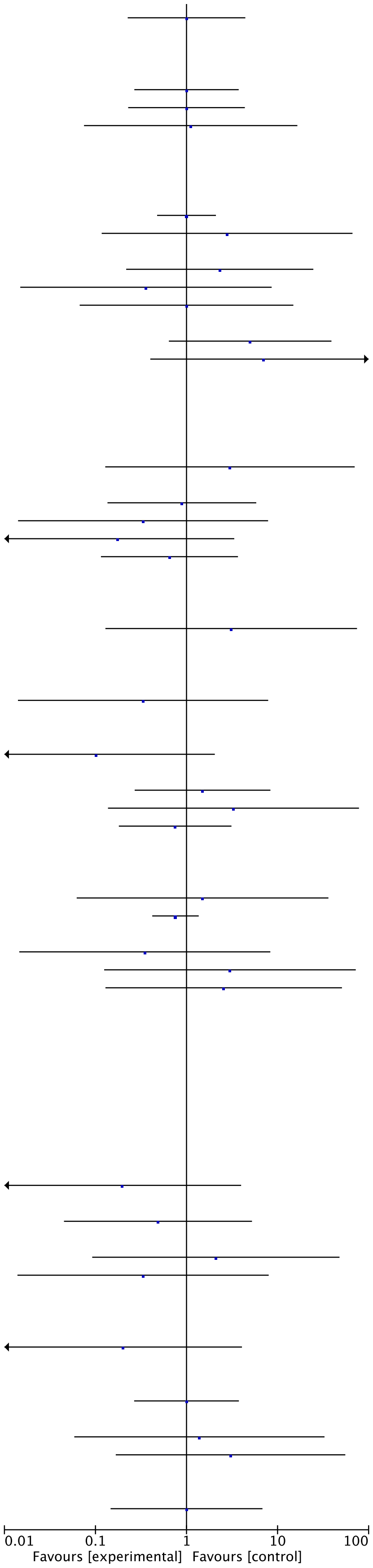

Tsutsumimoto 2011	0	20	0	20		Not estimable
Vara 2017	0	53	0	49		Not estimable
Veien 2002	0	15	0	15		Not estimable
Vela 2012	3	22	3	22	0.7%	1.00 [0.23, 4.42]
Verma 2014	0	36	0	47		Not estimable
Vijay 2013	0	45	0	45		Not estimable
Volquind 2014	0	32	0	30		Not estimable
Wang 2013	0	30	0	30		Not estimable
Wang 2016	1	81	0	38	0.2%	1.43 [0.06, 34.24]
Wang 2018	15	39	16	41	5.1%	0.99 [0.57, 1.71]
Wang, J 2016	10	100	10	98	2.2%	0.98 [0.43, 2.25]
Watts 2017	2	69	3	69	0.5%	0.67 [0.11, 3.87]
Wong 2008	0	73	1	73	0.2%	0.33 [0.01, 8.05]
Xie 2015	1	45	1	45	0.2%	1.00 [0.06, 15.50]
Xu 2012	0	20	0	20		Not estimable
Xu 2019	1	68	0	67	0.2%	2.96 [0.12, 71.31]
Yamasaki 2004	0	20	0	20		Not estimable
Yamasaki 2005	0	21	0	21		Not estimable
Ye 2019	20	160	2	40	0.8%	2.50 [0.61, 10.26]
Yen 2017	0	33	0	32		Not estimable
Yi 2016	2	50	1	50	0.3%	2.00 [0.19, 21.36]
Yuan 2017	2	140	1	140	0.3%	2.00 [0.18, 21.81]
Zekcer 2016	0	30	4	30	0.2%	0.11 [0.01, 1.98]
Zhang 2016	1	25	2	25	0.3%	0.50 [0.05, 5.17]
Zhang 2020	3	61	1	61	0.3%	3.00 [0.32, 28.04]
Zhang 2021	6	106	2	59	0.6%	1.67 [0.35, 8.01]
Zhang S 2020	0	32	2	32	0.2%	0.20 [0.01, 4.01]
Zhao 2018	0	40	0	40		Not estimable
Zhou 2018	0	58	0	58		Not estimable
Zhou 2019	2	50	3	50	0.5%	0.67 [0.12, 3.82]
Zhu 2020	11	100	4	50	1.3%	1.38 [0.46, 4.10]
Zohar 2004	0	40	0	20		Not estimable
Zufferey 2010	5	57	3	53	0.8%	1.55 [0.39, 6.17]
**Subtotal (95% CI)**		**5910**		**5048**	**32.3%**	**1.00 [0.80, 1.24]**
Total events	173		133			

Heterogeneity: $Tau^2 = 0.00$; $Chi^2 = 27.91$, df = 55 (P = 1.00); $I^2 = 0\%$
Test for overall effect: Z = 0.04 (P = 0.97)

12.1.5 Intracranial haemorrhage

Chandra 1978	0	20	0	19		Not estimable
Fodstad 1981	6	30	3	29	0.9%	1.93 [0.53, 7.01]
Kaste 1979	0	32	0	32		Not estimable
Ross 2000	4	229	0	233	0.2%	9.16 [0.50, 169.11]
Sprigg 2014	1	16	0	8	0.2%	1.59 [0.07, 35.15]
Sprigg 2018	39	1161	37	1164	7.9%	1.06 [0.68, 1.64]
Tsementzis 1990	0	50	0	50		Not estimable
Vermeulen 1984	20	241	18	238	4.1%	1.10 [0.60, 2.02]
**Subtotal (95% CI)**		**1779**		**1773**	**13.2%**	**1.15 [0.82, 1.62]**
Total events	70		58			

Heterogeneity: $Tau^2 = 0.00$; $Chi^2 = 2.80$, df = 4 (P = 0.59); $I^2 = 0\%$
Test for overall effect: Z = 0.83 (P = 0.41)

12.1.6 Gastrointestinal haemorrhage

Engqvist 1979	2	76	0	73	0.2%	4.81 [0.23, 98.42]
Roberts 2020	51	5994	28	6015	7.3%	1.83 [1.15, 2.89]
von Holstein 1987	1	72	0	82	0.2%	3.41 [0.14, 82.44]
**Subtotal (95% CI)**		**6142**		**6170**	**7.6%**	**1.89 [1.21, 2.96]**
Total events	54		28			

Heterogeneity: $Tau^2 = 0.00$; $Chi^2 = 0.52$, df = 2 (P = 0.77); $I^2 = 0\%$
Test for overall effect: Z = 2.78 (P = 0.006)

12.1.7 Others

Alimian 2011	0	42	0	42		Not estimable
Apipan 2018	0	60	0	20		Not estimable
Auvinen 1987	0	39	0	37		Not estimable
Avvisati 1989	0	6	0	6		Not estimable
Boylan 1996	0	25	0	20		Not estimable
Cansancao 2018	0	10	0	10		Not estimable
Choi 2009	0	32	0	29		Not estimable
Christabel 2014	0	25	0	24		Not estimable
Clave 2019	0	154	0	75		Not estimable
Cohen 2021	0	27	0	17		Not estimable
Crescenti 2011	1	100	4	100	0.3%	0.25 [0.03, 2.20]
Dadure 2011	0	19	0	20		Not estimable
Eldaba 2013	0	50	0	50		Not estimable
Fenger-Eriksen 2019	0	15	0	15		Not estimable
Goobie 2011	0	23	0	20		Not estimable
Kulkarni 2016	0	120	0	120		Not estimable
Meng 2019	0	30	0	30		Not estimable
Prasad 2018	0	40	0	20		Not estimable
Ramezani 2005	0	32	0	30		Not estimable
Vel 2015	0	50	0	50		Not estimable
Wu 2006	0	108	0	106		Not estimable
Zaman 2019	0	88	0	88		Not estimable
**Subtotal (95% CI)**		**1095**		**929**	**0.3%**	**0.25 [0.03, 2.20]**
Total events	1		4			

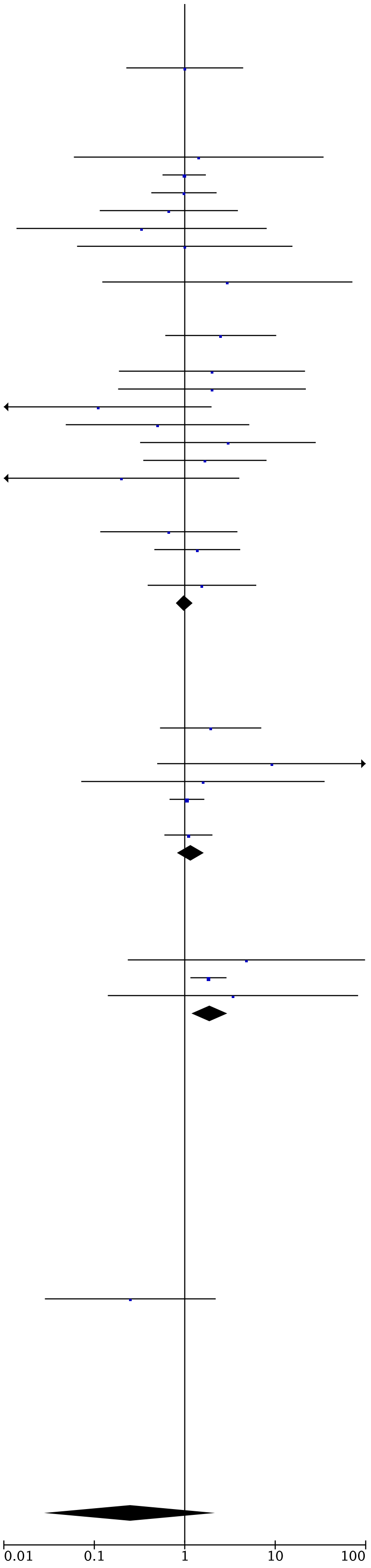

Heterogeneity: Not applicable
Test for overall effect: Z = 1.25 (P = 0.21)

**Total (95% CI)**	**50037**	**48447**	**100.0%**	**1.04 [0.92, 1.17]**
Total events	532	454		

Heterogeneity: $Tau^2 = 0.00$; $Chi^2 = 49.90$, df = 78 (P = 0.99); $I^2 = 0\%$
Test for overall effect: Z = 0.59 (P = 0.56)
Test for subgroup differences: $Chi^2 = 10.33$, df = 6 (P = 0.11), $I^2 = 41.9\%$

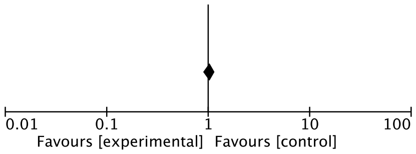

Figure S12. Forest plot of the acute coronary syndrome: subgroup analysis by underlying disease.

Study or Subgroup	Experimental Events	Experimental Total	Control Events	Control Total	Weight	Risk Ratio M-H, Random, 95% CI
**13.1.1 Trauma**						
Ebrahimi 2019	0	40	0	40		Not estimable
Jokar 2017	0	40	0	40		Not estimable
Roberts 2019	18	6406	20	6331	3.9%	0.89 [0.47, 1.68]
Rowell 2020	5	657	1	309	0.3%	2.35 [0.28, 20.04]
Shakur 2010	35	10093	55	10114	8.9%	0.64 [0.42, 0.97]
**Subtotal (95% CI)**		**17236**		**16834**	**13.2%**	**0.73 [0.51, 1.03]**
Total events	58		76			
Heterogeneity: Tau² = 0.00; Chi² = 1.91, df = 2 (P = 0.38); I² = 0%						
Test for overall effect: Z = 1.78 (P = 0.07)						
**13.1.2 Obstetric and gynecology**						
Abdul 2019	0	40	0	40		Not estimable
Goswami 2013	0	60	0	30		Not estimable
Gungorduk 2011	0	330	0	330		Not estimable
Maged 2015	0	100	0	100		Not estimable
Sahu 2019	0	50	0	50		Not estimable
Sallam 2019	0	43	0	43		Not estimable
Shakur 2017	2	10051	3	10009	0.5%	0.66 [0.11, 3.97]
Topsoee 2016	0	165	0	167		Not estimable
**Subtotal (95% CI)**		**10839**		**10769**	**0.5%**	**0.66 [0.11, 3.97]**
Total events	2		3			
Heterogeneity: Not applicable						
Test for overall effect: Z = 0.45 (P = 0.65)						
**13.1.3 Cardiac surgery**						
Ahn 2012	1	38	0	38	0.2%	3.00 [0.13, 71.40]
Alizadeh Ghavidel 2015	8	100	6	100	1.5%	1.33 [0.48, 3.70]
Altun 2017	0	34	0	20		Not estimable
Andreasen 2004	0	21	0	23		Not estimable
Armellin 2001	1	143	0	140	0.2%	2.94 [0.12, 71.50]
Brown 1997	0	60	0	30		Not estimable
Bulutcu 2005	0	25	0	25		Not estimable
Casati 2001	6	341	3	169	0.8%	0.99 [0.25, 3.91]
Casati 2002	1	30	1	30	0.2%	1.00 [0.07, 15.26]
Chakravarthy 2012	0	25	0	25		Not estimable
Diprose 2005	5	62	4	61	1.0%	1.23 [0.35, 4.36]
Esfandiari 2012	4	75	1	75	0.3%	4.00 [0.46, 34.96]
Faraoni 2014	0	24	0	12		Not estimable
Greiff 2012	3	30	4	33	0.8%	0.82 [0.20, 3.39]
Hardy 1998	1	43	2	45	0.3%	0.52 [0.05, 5.56]
Horrow 1991	1	37	0	44	0.2%	3.55 [0.15, 84.69]
Horrow 1995	0	121	0	27		Not estimable
Jares 2003	1	22	1	25	0.2%	1.14 [0.08, 17.11]
Karski 1995	0	99	0	48		Not estimable
Karski 2005	2	147	3	165	0.5%	0.75 [0.13, 4.42]
Katsaros 1996	0	104	0	106		Not estimable
Kuitunen 2005	1	20	1	20	0.2%	1.00 [0.07, 14.90]
Later 2009	0	99	8	103	0.2%	0.06 [0.00, 1.05]
Maddali 2007	0	111	0	111		Not estimable
Mansouri 2012	0	30	0	30		Not estimable
Mehr-Aein 2007	0	33	0	33		Not estimable
Misfeld 1998	0	14	0	14		Not estimable
Monaco 2020	2	50	2	50	0.4%	1.00 [0.15, 6.82]
Myles 2016	269	2311	300	2320	67.2%	0.90 [0.77, 1.05]
Nejad 2011	2	50	4	50	0.6%	0.50 [0.10, 2.61]
Pleym 2003	0	40	0	40		Not estimable
Reid 1997	0	20	0	21		Not estimable
Santos 2006	0	29	2	31	0.2%	0.21 [0.01, 4.26]
Shi 2013	3	274	6	278	0.8%	0.51 [0.13, 2.01]
Shi, J 2013	2	58	3	59	0.5%	0.68 [0.12, 3.91]
Shore-Lesserson 1996	1	17	2	13	0.3%	0.38 [0.04, 3.77]
Taghaddomi 2009	0	50	0	50		Not estimable
Vanek 2005	0	32	1	30	0.2%	0.31 [0.01, 7.40]
Wang 2012	0	116	0	115		Not estimable
Wei 2006	0	36	0	40		Not estimable
Zabeeda 2002	0	25	0	25		Not estimable
Zhang 2018	0	105	0	105		Not estimable
**Subtotal (95% CI)**		**5101**		**4779**	**76.8%**	**0.90 [0.78, 1.04]**
Total events	314		354			
Heterogeneity: Tau² = 0.00; Chi² = 11.34, df = 21 (P = 0.96); I² = 0%						
Test for overall effect: Z = 1.48 (P = 0.14)						
**13.1.4 Orthopaedic surgery**						
Aguilera 2013	0	41	0	42		Not estimable
Alvarez 2019	0	22	0	22		Not estimable

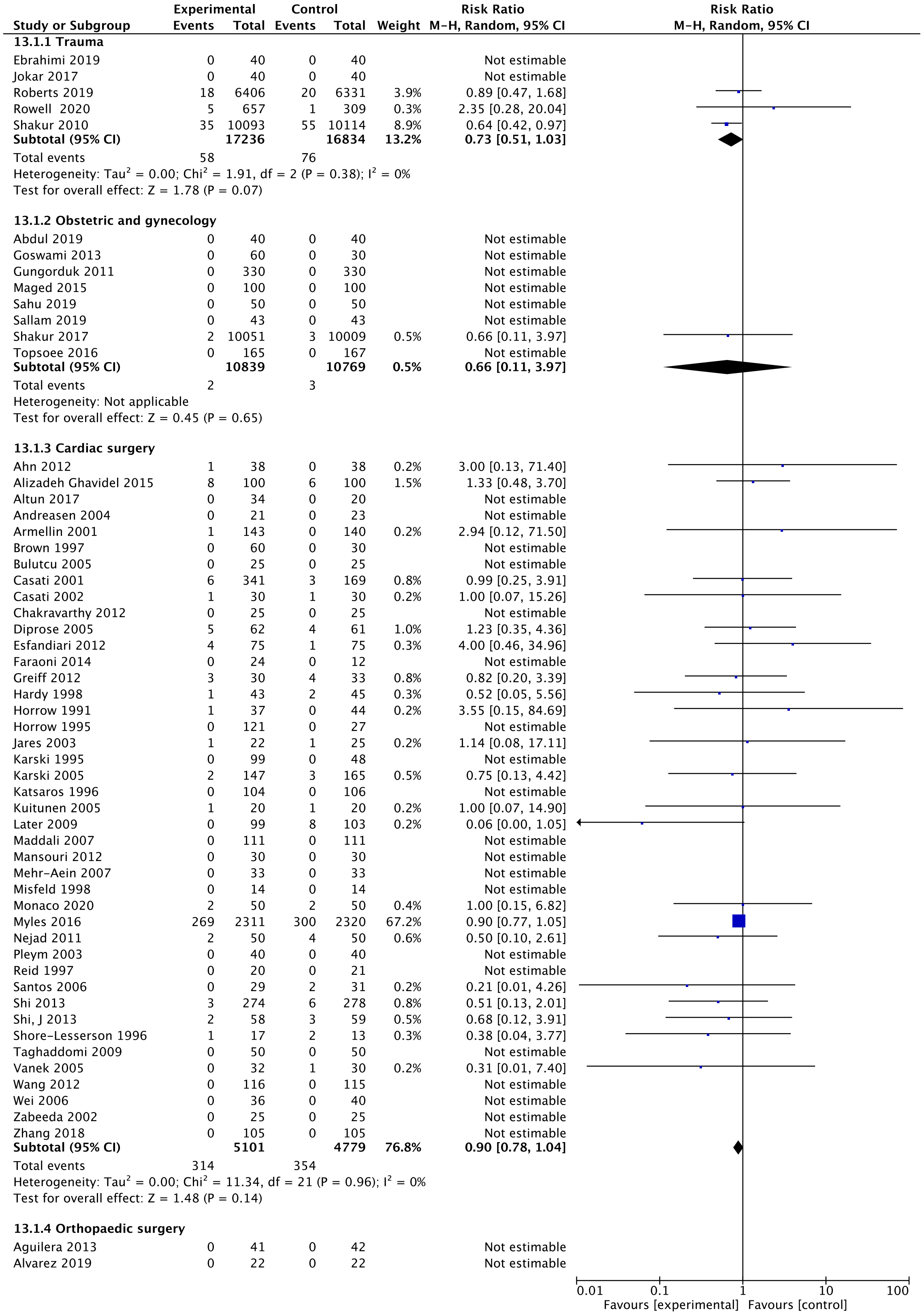

Baruah 2016	0	30	0	30		Not estimable
Benoni 2001	0	18	0	20		Not estimable
Chen 2019	0	88	0	88		Not estimable
Chin 2020	0	42	1	39	0.2%	0.31 [0.01, 7.39]
Clave 2019	1	154	0	75	0.2%	1.47 [0.06, 35.69]
Colomina 2016	0	44	0	51		Not estimable
Farrokhi 2011	0	38	0	38		Not estimable
Goobie 2018	0	56	0	55		Not estimable
Hiippala 1995	1	15	0	13	0.2%	2.63 [0.12, 59.40]
Kazemi 2010	0	32	0	32		Not estimable
Lei 2020	0	150	0	50		Not estimable
Luo 2019	0	44	0	46		Not estimable
Motififard 2015	0	45	0	45		Not estimable
Painter 2018	1	71	1	69	0.2%	0.97 [0.06, 15.23]
Palija 2020	0	40	0	40		Not estimable
Raksakietisak 2015	0	39	0	39		Not estimable
Seddighi 2019	0	20	0	20		Not estimable
Seviciu 2016	0	29	0	32		Not estimable
Shi 2017	0	50	0	46		Not estimable
Tengberg 2016	0	35	0	40		Not estimable
Thipparampall 2020	0	39	0	20		Not estimable
Tsutsumimoto 2011	0	20	0	20		Not estimable
Vara 2017	0	53	1	49	0.2%	0.31 [0.01, 7.40]
Wang 2013	0	30	0	30		Not estimable
Watts 2017	2	69	3	69	0.5%	0.67 [0.11, 3.87]
Wong 2008	1	73	0	73	0.2%	3.00 [0.12, 72.45]
Xie 2015	1	45	0	45	0.2%	3.00 [0.13, 71.74]
Zhang 2020	0	61	0	61		Not estimable
Zhang 2021	0	106	0	59		Not estimable
Zhang S 2020	0	32	0	32		Not estimable
Zhou 2019	0	50	2	50	0.2%	0.20 [0.01, 4.06]
Zhu 2020	0	100	0	50		Not estimable
Zufferey 2010	0	57	0	53		Not estimable
**Subtotal (95% CI)**		**1838**		**1543**	**1.9%**	**0.85 [0.34, 2.14]**
Total events	7		8			
Heterogeneity: Tau² = 0.00; Chi² = 3.57, df = 8 (P = 0.89); I² = 0%						
Test for overall effect: Z = 0.35 (P = 0.73)						
**13.1.5 Intracranial surgery**						
Fodstad 1981	2	30	0	29	0.2%	4.84 [0.24, 96.66]
Kaste 1979	0	32	0	32		Not estimable
Sprigg 2014	0	16	0	8		Not estimable
Sprigg 2018	11	1161	6	1164	1.6%	1.84 [0.68, 4.95]
**Subtotal (95% CI)**		**1239**		**1233**	**1.8%**	**2.02 [0.79, 5.18]**
Total events	13		6			
Heterogeneity: Tau² = 0.00; Chi² = 0.36, df = 1 (P = 0.55); I² = 0%						
Test for overall effect: Z = 1.47 (P = 0.14)						
**13.1.6 Gastrointestinal haemorrhage**						
Engqvist 1979	2	76	0	73	0.2%	4.81 [0.23, 98.42]
Roberts 2020	24	5994	28	6015	5.4%	0.86 [0.50, 1.48]
**Subtotal (95% CI)**		**6070**		**6088**	**5.6%**	**1.05 [0.36, 3.08]**
Total events	26		28			
Heterogeneity: Tau² = 0.26; Chi² = 1.22, df = 1 (P = 0.27); I² = 18%						
Test for overall effect: Z = 0.08 (P = 0.93)						
**13.1.7 Others**						
Alimian 2011	0	42	0	42		Not estimable
Auvinen 1987	0	39	0	37		Not estimable
Boylan 1996	0	25	0	20		Not estimable
Cansancao 2018	0	10	0	10		Not estimable
Cohen 2021	0	27	0	17		Not estimable
Crescenti 2011	1	100	1	100	0.2%	1.00 [0.06, 15.77]
Dadure 2011	0	19	0	20		Not estimable
Fenger-Eriksen 2019	0	15	0	15		Not estimable
Goobie 2011	0	23	0	20		Not estimable
Ramezani 2005	0	32	0	30		Not estimable
Wu 2006	0	108	0	106		Not estimable
**Subtotal (95% CI)**		**440**		**417**	**0.2%**	**1.00 [0.06, 15.77]**
Total events	1		1			
Heterogeneity: Not applicable						
Test for overall effect: Z = 0.00 (P = 1.00)						
**Total (95% CI)**		**42763**		**41663**	**100.0%**	**0.88 [0.78, 1.00]**
Total events	421		476			
Heterogeneity: Tau² = 0.00; Chi² = 22.68, df = 39 (P = 0.98); I² = 0%						
Test for overall effect: Z = 1.91 (P = 0.06)						
Test for subgroup differences: Chi² = 4.40, df = 6 (P = 0.62), I² = 0%						

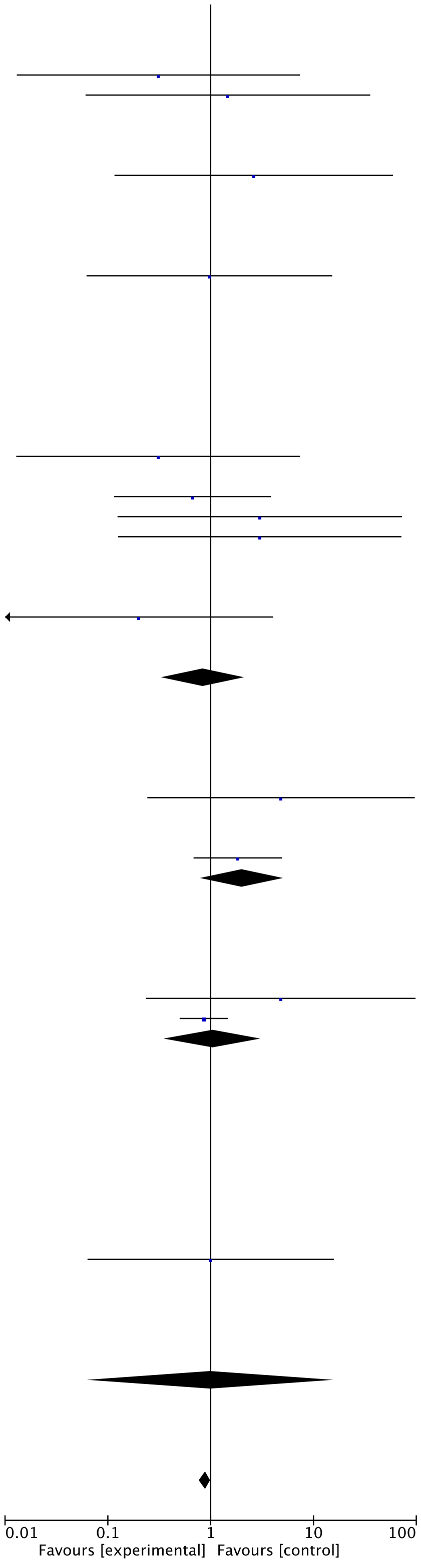

Figure S13. Forest plot of the stroke: subgroup analysis by underlying disease.

Study or Subgroup	Experimental Events	Experimental Total	Control Events	Control Total	Weight	Risk Ratio M-H, Random, 95% CI
**10.1.1 Trauma**						
Ebrahimi 2019	0	40	0	40		Not estimable
Jokar 2017	0	40	0	40		Not estimable
Roberts 2019	46	6406	42	6331	9.2%	1.08 [0.71, 1.64]
Rowell 2020	16	647	10	309	2.6%	0.76 [0.35, 1.66]
Shakur 2010	57	10092	67	10114	12.9%	0.85 [0.60, 1.21]
Yutthakasemsunt 2013	0	120	3	118	0.2%	0.14 [0.01, 2.69]
**Subtotal (95% CI)**		**17345**		**16952**	**25.0%**	**0.91 [0.71, 1.17]**
Total events	119		122			
Heterogeneity: $Tau^2 = 0.00$; $Chi^2 = 2.53$, df = 3 (P = 0.47); $I^2 = 0\%$						
Test for overall effect: Z = 0.74 (P = 0.46)						
**10.1.2 Obstetric and gynecology**						
Abdul 2019	0	40	0	40		Not estimable
Goswami 2013	0	60	0	30		Not estimable
Gungorduk 2011	0	330	0	330		Not estimable
Maged 2015	0	100	0	100		Not estimable
Sahu 2019	0	50	0	50		Not estimable
Sallam 2019	0	43	0	43		Not estimable
Shakur 2017	8	10051	6	10009	1.4%	1.33 [0.46, 3.83]
Topsoee 2016	0	165	0	167		Not estimable
**Subtotal (95% CI)**		**10839**		**10769**	**1.4%**	**1.33 [0.46, 3.83]**
Total events	8		6			
Heterogeneity: Not applicable						
Test for overall effect: Z = 0.53 (P = 0.60)						
**10.1.3 Cardiac surgery**						
Ahn 2012	0	38	0	38		Not estimable
Altun 2017	0	34	0	20		Not estimable
Andreasen 2004	0	21	0	23		Not estimable
Brown 1997	0	60	0	30		Not estimable
Bulutcu 2005	0	25	0	25		Not estimable
Casati 2002	1	30	2	30	0.3%	0.50 [0.05, 5.22]
Casati 2004	0	52	0	50		Not estimable
Chauhan 2003	0	96	0	24		Not estimable
Esfandiari 2012	3	75	5	75	0.8%	0.60 [0.15, 2.42]
Faraoni 2014	0	24	0	12		Not estimable
Greiff 2012	0	30	1	33	0.2%	0.37 [0.02, 8.65]
Hardy 1998	1	43	0	45	0.2%	3.14 [0.13, 74.95]
Horrow 1991	1	37	0	44	0.2%	3.55 [0.15, 84.69]
Horrow 1995	0	121	0	27		Not estimable
Jares 2003	0	22	0	25		Not estimable
Karski 1995	3	99	0	48	0.2%	3.43 [0.18, 65.10]
Karski 2005	1	147	1	165	0.2%	1.12 [0.07, 17.79]
Katsaros 1996	3	104	2	106	0.5%	1.53 [0.26, 8.96]
Kuitunen 2005	0	20	0	20		Not estimable
Later 2009	1	99	1	103	0.2%	1.04 [0.07, 16.41]
Mansouri 2012	0	30	0	30		Not estimable
Mehr-Aein 2007	0	33	0	33		Not estimable
Misfeld 1998	0	14	0	14		Not estimable
Myles 2016	32	2311	35	2320	7.1%	0.92 [0.57, 1.48]
Nejad 2011	0	50	1	50	0.2%	0.33 [0.01, 7.99]
Pleym 2003	0	40	0	40		Not estimable
Reid 1997	0	20	0	21		Not estimable
Santos 2006	2	29	0	31	0.2%	5.33 [0.27, 106.61]
Shi 2013	2	274	1	278	0.3%	2.03 [0.19, 22.25]
Shi, J 2013	1	58	0	59	0.2%	3.05 [0.13, 73.39]
Shimizu 2011	1	81	0	79	0.2%	2.93 [0.12, 70.79]
Shore-Lesserson 1996	2	17	1	13	0.3%	1.53 [0.15, 15.09]
Taghaddomi 2009	0	50	0	50		Not estimable
Vanek 2005	0	32	0	30		Not estimable
Wang 2012	0	116	0	115		Not estimable
Wei 2006	0	36	0	40		Not estimable
Zabeeda 2002	0	25	0	25		Not estimable
Zhang 2018	0	105	0	105		Not estimable
**Subtotal (95% CI)**		**4498**		**4276**	**11.0%**	**1.02 [0.70, 1.50]**
Total events	54		50			
Heterogeneity: $Tau^2 = 0.00$; $Chi^2 = 6.42$, df = 15 (P = 0.97); $I^2 = 0\%$						
Test for overall effect: Z = 0.13 (P = 0.90)						
**10.1.4 Orthopaedic surgery**						
Aguilera 2013	1	41	0	42	0.2%	3.07 [0.13, 73.29]
Alvarez 2019	0	22	0	22		Not estimable
Baruah 2016	0	30	0	30		Not estimable
Benoni 2001	0	18	0	20		Not estimable
Chen 2019	2	88	0	88	0.2%	5.00 [0.24, 102.67]
Chin 2020	0	42	0	39		Not estimable
Colomina 2016	0	44	0	51		Not estimable
Emara 2014	1	20	0	20	0.2%	3.00 [0.13, 69.52]

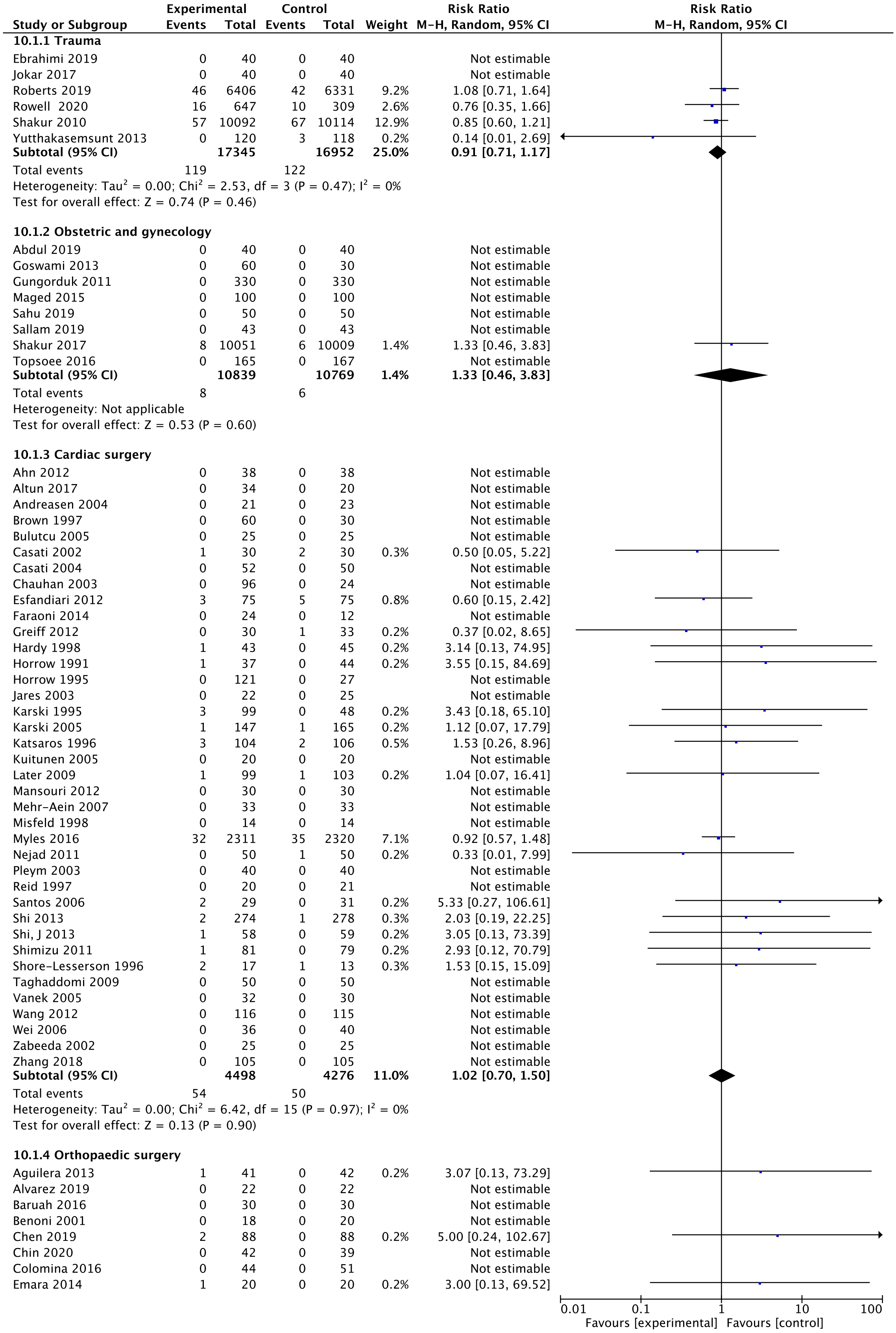

Chin 2020	0	42	0	39		Not estimable
Colomina 2016	0	44	0	51		Not estimable
Emara 2014	1	20	0	20	0.2%	3.00 [0.13, 69.52]
Farrokhi 2011	0	38	0	38		Not estimable
Goobie 2018	0	56	0	55		Not estimable
Hiippala 1995	0	15	0	13		Not estimable
Kazemi 2010	0	32	0	32		Not estimable
Lei 2020	0	150	0	50		Not estimable
Luo 2019	0	44	3	46	0.2%	0.15 [0.01, 2.81]
Motififard 2015	0	45	0	45		Not estimable
Painter 2018	0	71	1	69	0.2%	0.32 [0.01, 7.82]
Palija 2020	0	40	0	40		Not estimable
Raksakietisak 2015	0	39	0	39		Not estimable
Seddighi 2019	0	20	0	20		Not estimable
Seviciu 2016	0	29	0	32		Not estimable
Shi 2017	8	50	6	46	1.7%	1.23 [0.46, 3.27]
Tengberg 2016	0	35	1	40	0.2%	0.38 [0.02, 9.03]
Thipparampall 2020	0	39	0	20		Not estimable
Watts 2017	1	69	0	69	0.2%	3.00 [0.12, 72.39]
Wong 2008	0	73	0	73		Not estimable
Xie 2015	0	45	0	45		Not estimable
Zaman 2019	0	88	0	88		Not estimable
Zhang 2020	0	61	0	61		Not estimable
Zhang 2021	0	106	0	59		Not estimable
Zhang S 2020	0	32	0	32		Not estimable
Zhu 2020	0	100	0	50		Not estimable
Zufferey 2010	3	57	0	53	0.2%	6.52 [0.34, 123.27]
**Subtotal (95% CI)**		**1639**		**1427**	**3.0%**	**1.31 [0.63, 2.72]**
Total events	16		11			
Heterogeneity: $Tau^2 = 0.00$; $Chi^2 = 6.15$, df = 8 (P = 0.63); $I^2 = 0\%$						
Test for overall effect: Z = 0.73 (P = 0.47)						
**10.1.5 Intracranial haemorrhage**						
Fodstad 1981	8	30	3	29	1.1%	2.58 [0.76, 8.77]
Hillman 2002	45	254	33	251	9.4%	1.35 [0.89, 2.04]
Kaste 1979	0	32	0	32		Not estimable
Ross 2000	79	229	84	233	26.2%	0.96 [0.75, 1.23]
Sprigg 2014	0	16	0	8		Not estimable
Sprigg 2018	16	1161	11	1164	2.8%	1.46 [0.68, 3.13]
Tsementzis 1990	22	50	11	50	4.3%	2.00 [1.09, 3.68]
Vermeulen 1984	59	241	36	238	11.5%	1.62 [1.11, 2.35]
**Subtotal (95% CI)**		**2013**		**2005**	**55.2%**	**1.40 [1.05, 1.86]**
Total events	229		178			
Heterogeneity: $Tau^2 = 0.06$; $Chi^2 = 10.38$, df = 5 (P = 0.07); $I^2 = 52\%$						
Test for overall effect: Z = 2.29 (P = 0.02)						
**10.1.6 Gastrointestinal haemorrhae**						
Engqvist 1979	0	76	2	73	0.2%	0.19 [0.01, 3.94]
Roberts 2020	19	5994	18	6015	3.9%	1.06 [0.56, 2.02]
**Subtotal (95% CI)**		**6070**		**6088**	**4.0%**	**0.87 [0.30, 2.54]**
Total events	19		20			
Heterogeneity: $Tau^2 = 0.23$; $Chi^2 = 1.18$, df = 1 (P = 0.28); $I^2 = 16\%$						
Test for overall effect: Z = 0.25 (P = 0.80)						
**10.1.7 Others**						
Alimian 2011	0	42	0	42		Not estimable
Auvinen 1987	0	39	0	37		Not estimable
Boylan 1996	0	25	0	20		Not estimable
Brown 1997	0	30	0	30		Not estimable
Cansancao 2018	0	10	0	10		Not estimable
Cohen 2021	0	27	0	17		Not estimable
Crescenti 2011	0	100	0	100		Not estimable
Dadure 2011	0	19	0	20		Not estimable
Fenger-Eriksen 2019	0	15	0	15		Not estimable
Goobie 2011	0	23	0	20		Not estimable
Ramezani 2005	0	32	0	30		Not estimable
Wu 2006	0	108	0	106		Not estimable
Zhou 2019	1	50	2	50	0.3%	0.50 [0.05, 5.34]
**Subtotal (95% CI)**		**520**		**497**	**0.3%**	**0.50 [0.05, 5.34]**
Total events	1		2			
Heterogeneity: Not applicable						
Test for overall effect: Z = 0.57 (P = 0.57)						
**Total (95% CI)**		**42924**		**42014**	**100.0%**	**1.12 [0.98, 1.27]**
Total events	446		389			
Heterogeneity: $Tau^2 = 0.00$; $Chi^2 = 31.75$, df = 38 (P = 0.75); $I^2 = 0\%$						
Test for overall effect: Z = 1.71 (P = 0.09)						
Test for subgroup differences: $Chi^2 = 5.96$, df = 6 (P = 0.43), $I^2 = 0\%$						

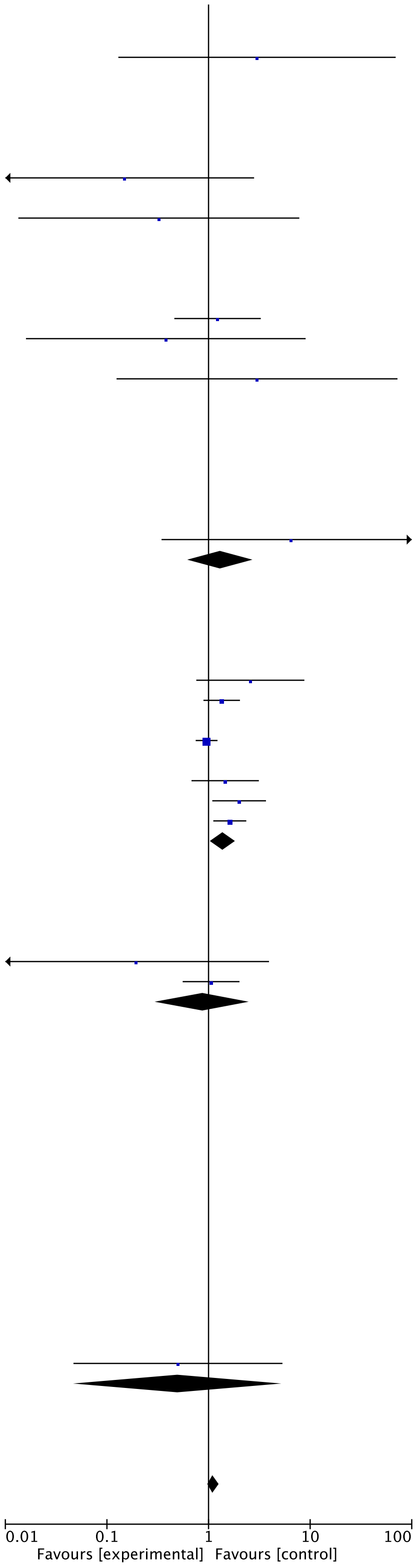

Supplement: Supplementary file 2 — Additional file 2. Figure S1 Risk of bias item presented as percentages. Figure S2 Risk of bias summary and graph. Figure S3 Funnel plot of the thrombotic events. Figure S4 Funnel plot of the seizures. Figure S5 Forest plot of the thrombotic events: subgroup analysis by underlying disease. Figure S6 Forest plot of the thrombotic events: subgroup analysis by TXA dose. Figure S7 Forest plot of the thrombotic events: subgroup analysis in children. Figure S8 Forest plot of the seizures: subgroup analysis by underlying disease. Figure S9 Forest plot of the seizures: subgroup analysis by TXA dose. Figure S10 Forest plot of the seizures: subgroup analysis in children. Figure S11 Forest plot of the venous thromboembolism: subgroup analysis by underlying disease. Figure S12 Forest plot of the acute coronary syndrome: subgroup analysis by underlying disease. Figure S13 Forest plot of the stroke: subgroup analysis by underlying disease severity. [file 13054_2021_3799_MOESM2_ESM.pdf]
